# Biomarker discovery and patient stratification in pancreatic cancer using incomplete multi-omics data

**DOI:** 10.1371/journal.pcbi.1014735

**Published:** 2026-09-10

**Authors:** Alejandra Paja-García, Rafael Romero-Becerra, Tero Aittokallio, Alberto López

**Affiliations:** 1 Department of Cancer Genetics, Institute for Cancer Research, Oslo University Hospital, Oslo, Norway; 2 Oslo Centre for Biostatistics and Epidemiology (OCBE), University of Oslo, Oslo, Norway; 3 Institute for Molecular Medicine Finland (FIMM), HiLIFE, University of Helsinki, Helsinki, Finland; Polytechnic University of Bari, ITALY

## Abstract

Pancreatic ductal adenocarcinoma (PDAC), with a 12% 5-year survival rate, is the most aggressive type of cancer. Early diagnosis for this pathology is rare, and conventional treatments such as surgery, radio- or chemotherapy, have little to no effect on reducing mortality. Machine learning (ML) approaches could be used to identify biomarkers that help clinicians stratify patients and improve treatment outcomes. However, most ML techniques perform poorly with incomplete data, which is usually the case in real-world settings, often forcing researchers to discard valuable information. In this study, unsupervised ML algorithms capable of dealing with missing modalities were applied to incomplete multi-omics data from PDAC patients to identify clinically meaningful patient subgroups. Through a large-scale clustering benchmark including six omics layers, we discovered two novel subgroups with statistically significant differences in survival and recurrence after surgery, particularly within the first two years, when most patient deaths occur, as well as distinct tumor mutational burden. Comprehensive multi-omics analyses revealed substantial molecular differences between patients in both groups, identified three methylation biomarkers to stratify patients, and highlighted dysregulation in key oncogenic pathways. Importantly, the identified groups are different from previous PDAC classifications, both in their patient composition, prognosis, and in the oncogenic gene pathway profiles exhibited. Using an independent cohort, we further demonstrated that both the prognostic value of these subtypes and their underlying biological characteristics are reproducible. These results could lead to better stratified treatment regimens to improve the prognosis of PDAC patients.

## Author summary

Pancreatic cancer has one of the highest mortality rates among all cancer types, and it is difficult to find common molecular characteristics across tumors that can be used to create new treatments for patients. Machine learning algorithms are been used to analyze the molecular data of cancer patients and identify similar groups, for example, patients that share the same mutations in specific genes. This information can then be used to create tailored therapies for individuals and obtain the best patient outcomes. We applied machine learning algorithms to molecular data from pancreatic cancer patients (e.g.,: DNA, mutations, proteins) and identified two novel groups, with one of the groups showing lower survival probability, and an increased risk of cancer recurrence compared with the other. Further analysis revealed key cellular pathways and that three molecules could be used to separate patients into the two groups. This opens avenues for therapies personalized for every individual, to ensure that pancreatic cancer patients receive the best care they can.

## Introduction

Pancreatic ductal adenocarcinoma (PDAC), which accounts for 90% of pancreatic tumors, has a mortality rate closely matching its incidence [1]. This malignancy is rarely detected early due to non-specific symptoms, lack of biomarkers, and challenges in imaging early-stage tumors. PDAC rapidly induces angiogenesis, leading to early metastasis, and shows resistance to chemotherapy, radiotherapy, and other targeted therapies. Consequently, most cases are diagnosed at advanced stages, resulting in a 12% 5-year survival rate. Even among the 15–20% of patients eligible for surgical resection, around 75% of patients develop recurrence within 2 years [2]. Currently, PDAC is the third cause of cancer-related deaths in developed countries and predicted to become the second leading cause of cancer mortality within the next decade [2]. This trend poses a major public health challenge and entails substantial economic costs due to increased healthcare spending [1].

The shift towards analysis of molecular and genomic data in PDAC tumors has provided important insights into the biological processes that contribute towards pancreatic cancer pathophysiology. Whole-exome sequencing studies have confirmed the presence of common mutations in oncogenes, such as KRAS, and the inactivation of tumor-suppressor genes TP53, SMAD4 and CDKN2A, which support PDAC tumor growth and proliferation [3]. Beyond genomic profiling, a holistic understanding of the molecular mechanisms involved in PDAC is urgently needed to improve patient outcomes through diagnostic and therapeutic avenues.

Machine Learning (ML) models are being used to assist clinicians and researchers in early diagnosis, biomarker detection, and prediction of response to treatment of PDAC patients. These computational methods can be applied on various molecular data types, such as genomics, transcriptomics, or proteomics, generally referred to as “omics,” to stratify PDAC patients according to their molecular characteristics. Previous PDAC subtyping has been identified through single-omic analyses of gene expression or DNA methylation data [4–6]. Studies on somatic copy number alterations (CNA) have also shown relationships between the inhibition of the PARP family genes, and its effect on drug response in PDAC patients [7]. These single-omic studies can be used in precision medicine to develop targeted therapies for improved prognosis of patients based on their molecular profiles. Furthermore, various omics layers can be integrated together to create multi-omics models, providing a more complete understanding of the disease processes.

However, most ML models require complete data, which are rarely available due to poor data quality or the high cost of data collection [8]. Although the challenges of missing information in data analysis have been explored in single-modality studies, they have received little attention in multi-modal research. There are several basic approaches to tackle missing information, such as removing samples with incomplete data, or missing value imputation. The former significantly reduces the data available for analysis and might remove samples containing valuable information at one or several omics levels, whilst the latter can lead to underlying biases. Multi-modal algorithms that can deal with incomplete data have recently been developed to overcome these issues [9].

**Fig 1:**
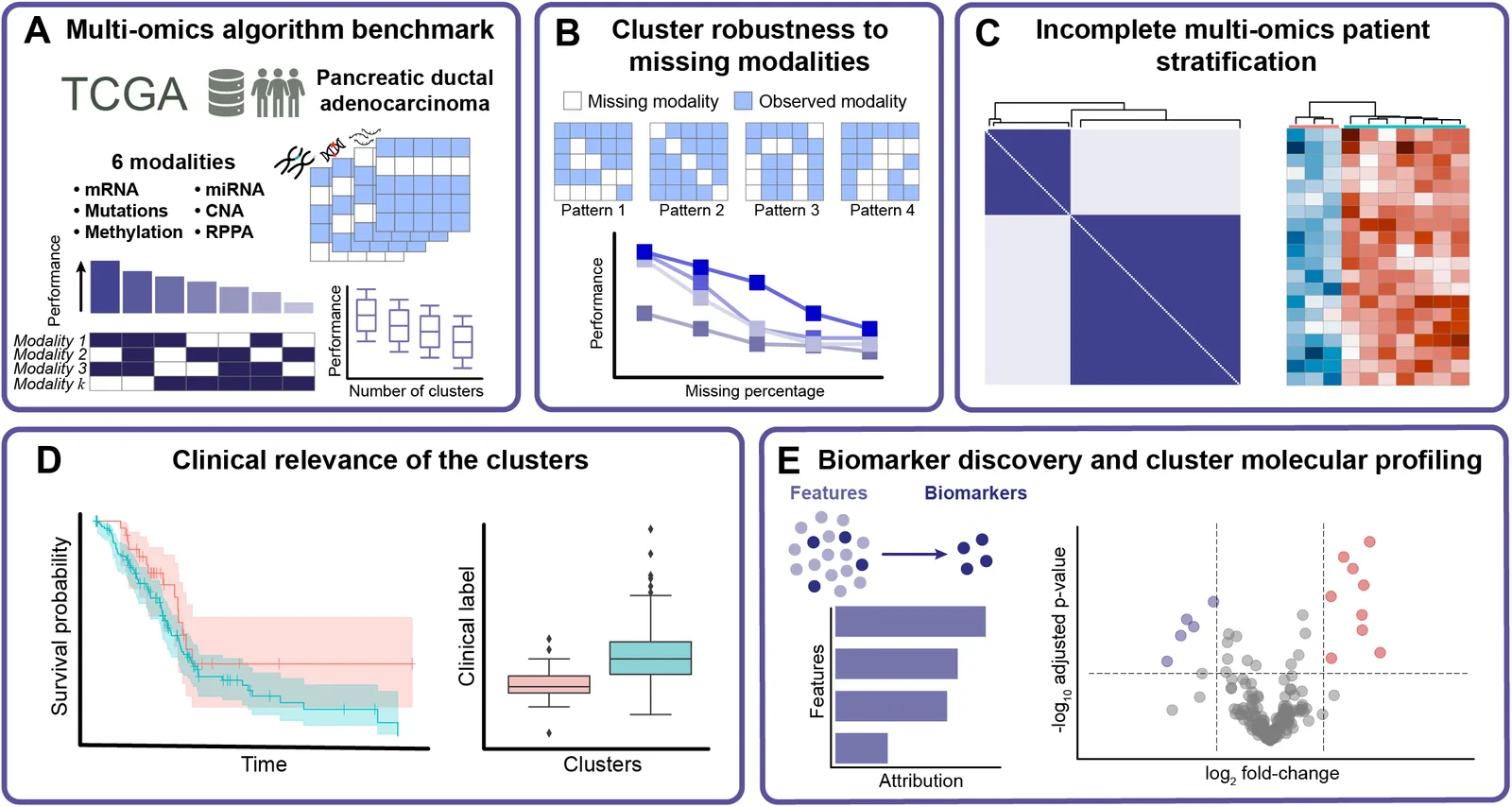
Systematic benchmark of TCGA incomplete multi-omics data from PDAC patients, discovery of clusters and consequent clinical and molecular analysis. **(A)** First, the best combination of modalities is identified by computing performance scores for all possible combinations between mRNA, miRNA, mutations, CNA, methylation and RPPA data. **(B)** Then, the robustness of nine state-of-the-art unsupervised clustering algorithms was evaluated using modality-wise missing data to identify the best method. **(C)** With these parameters, multi-omics patient stratification was performed on incomplete data. **(D, E)** The clinical (D) and molecular (E) relevance of the clusters was finally assessed.

In this work, we analyzed incomplete multi-omics data from PDAC patients obtained from The Cancer Genome Atlas (TCGA). We evaluated both the performance of the algorithms and the novelty, clinical relevance, and molecular characteristics of the resulting clustering solutions (Fig 1). The key contributions of this work are:

- Systematic benchmarking of nine state-of-the-art unsupervised clustering algorithms across six omics data types with modality-wise incomplete data, leading to the identification of the optimal method (NEMO) and most informative modality combination (DNA methylation and somatic copy number alterations). This constitutes the largest benchmark to date using incomplete multi-omics data.
- Discovery of novel subtypes of PDAC patients, where the poor-prognosis group has double the recurrence risk and mortality within the first year after diagnosis, and higher tumor mutational burden (TMB). Subsequent integrated molecular profiling highlight key oncogenic pathways, providing insights into potential therapeutic strategies.
- Identification of three methylation biomarkers with multi-modal feature selection that group patients into the clusters.
- Release of a patient stratification model to facilitate stratification of new samples and clinical translation.

## Results

### Copy number and methylation as the best combination of omic modalities

**Fig 2:**
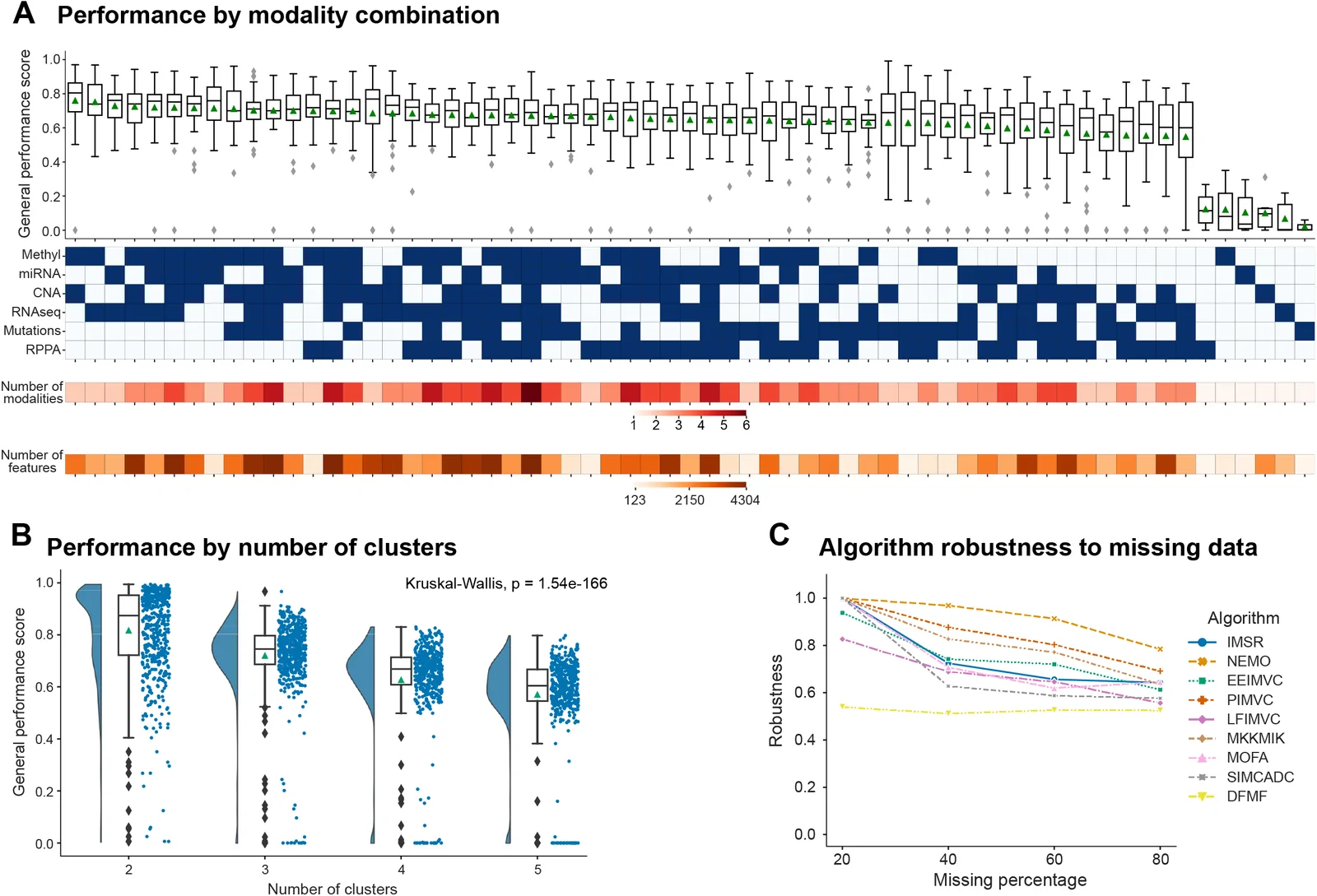
Benchmarking of modality combinations, number of clusters, and algorithm robustness. **(A)** Evaluation of the clustering performance across the different combination of omic modalities. The most informative combination was DNA methylation and copy number alterations. The general performance score was computed as the harmonic mean of silhouette and adjusted mutual information scores, reflecting cluster compactness and stability, respectively. Combinations are ordered by decreasing mean score (green triangle). First heatmap indicates modalities present in combination (blue), second heatmap indicates total number of modalities in each combination, and third heatmap shows total number of features in each combination. **(B)** Evaluation of the clustering performance for different number of clusters. Two-cluster solutions consistently outperformed those with a higher number of clusters. The number of clusters are ordered by decreasing mean general performance score (green triangle). **(C)** Comparison of algorithm robustness to different percentages of missing data. NEMO demonstrated the most robust performance across all levels of missing data, shown as the average across all missingness patterns. Robustness was quantified by comparing clusters obtained with missing data to those found in complete data using the adjusted mutual information score.

We first analyzed the effect of data modalities on the clustering solutions. The combination of CNA and methylation data had the best performance on average (Fig 2A) across all algorithms and numbers of clusters, indicating that the clusters formed when using this combination of modalities were the most stable and compact. This combination also had the best performance when using the individual metrics (S1 Fig). The single-modality methods ranked at the lowest end of the performance comparison, indicating that integrating multiple omics layers provides a clear advantage in terms of cluster quality and stability.

We also investigated the influence of individual modalities, the algorithm and the number of clusters on the clustering solutions. Combinations that included methylation achieved the highest overall performance, being in 9 of the top 10 results. This is confirmed when calculating the individual importance of the modalities, as combinations with methylation obtained the highest metrics (S2 Fig). Additionally, the IMSR algorithm achieved the best performance on average (S3 Fig). Overall, two-cluster solutions outperformed the alternatives with higher number of clusters (Figs 2B and S4), consistently exhibiting high values of the general performance score.

### NEMO provides the most robust performance with missing data

Since patients had missing omic modalities, the robustness of the clustering algorithms was assessed using CNA and methylation data with two clusters (the best parameters) at increasing fractions of incomplete data. Overall, NEMO exhibited the most robust performance throughout all levels of missing data, even when only 20% of samples have all modalities available (Fig 2C). Algorithm performance was consistent across missingness patterns, indicating little effect of the pattern on model performance (S5 Fig).

In terms of GPS and robustness, this approach outperformed also standard imputation-based workflows (mean, median and K-nearest neighbors) (S6 Fig), where missing modalities were imputed before applying NEMO. Overall, these findings indicate that, in this setting, NEMO’s ability to operate directly on incomplete multi-omics data provides a clear advantage over a standard imputation-based strategies, yielding both greater robustness and improved clustering performance.

### Robust final clusters formed applying the NEMO algorithm to methylation and CNA data

The final clusters (termed IMOC, for Incomplete Multi-Omics Clustering) were identified using consensus clustering with NEMO for all patients with DNA methylation and CNA data, provided that at least one omic modality was available: cluster 1 with 33 patients, and cluster 2 with 121 patients.

**Fig 3:**
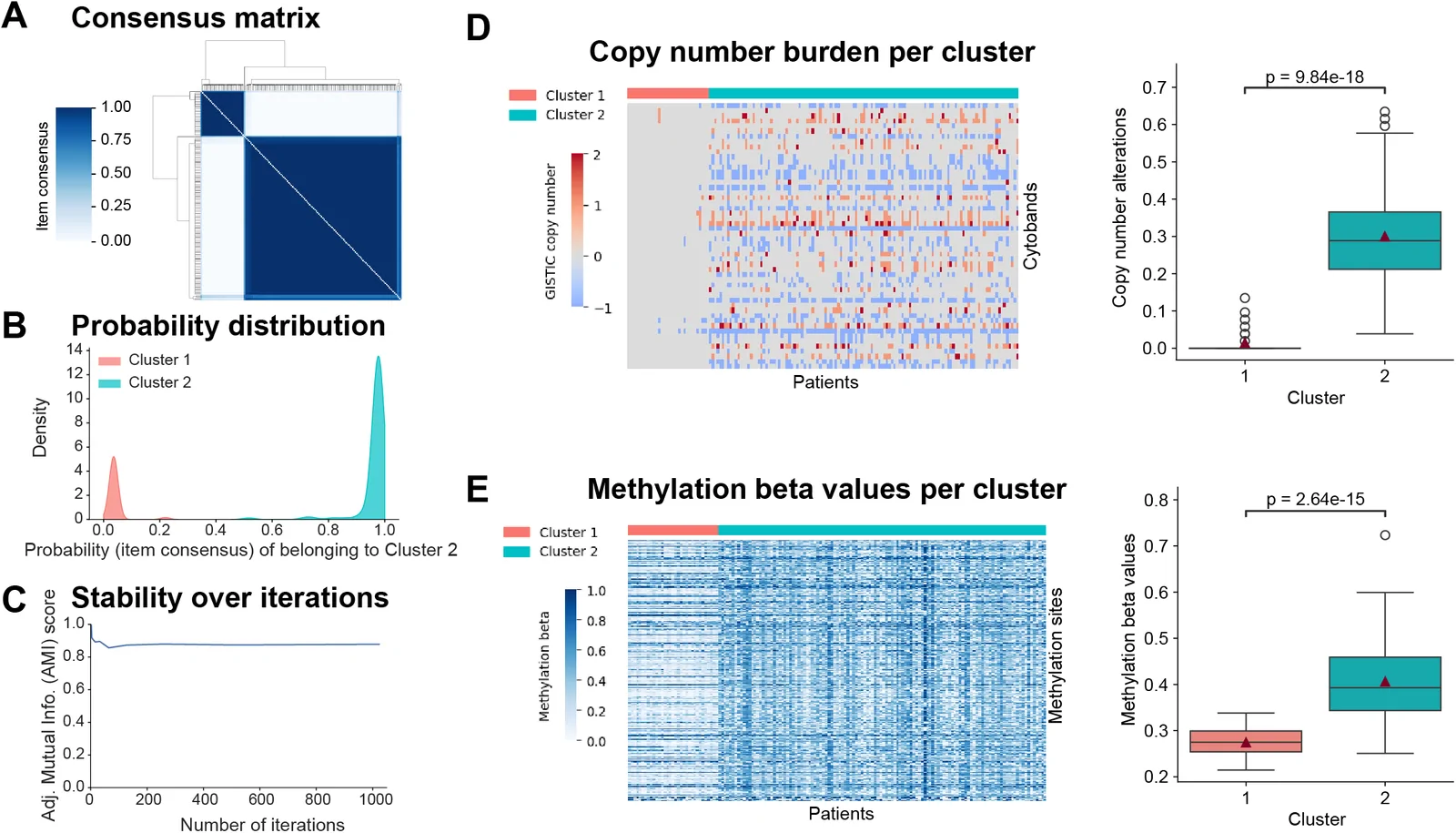
Analysis of final IMOC clusters. **(A)** The consensus matrix is nearly perfect, with almost all patients having values close to 0 or 1. A consensus value of 1 indicates patients that are always clustered together. **(B)** The pronounced peaks at the extremes for both clusters indicated high cluster agreement and stability. A kernel density estimation was applied to visualize a continuous probability density curve. **(C)** IMOC cluster stability remained high and consistent across all iteration numbers, indicating stability independent of this hyperparameter. **(D)** Patients in cluster 2 exhibited higher overall rates of copy number alterations. Differences between clusters were calculated by averaging the copy number alterations (in absolute values) per patient, and finding differences between the averages in each cluster. **(E)** Patients in cluster 2 had a predominantly hypermethylated DNA pattern. In (D) and **(E)**, each point represents a patient, computed as the average across features. Statistical significance was assessed using the Kruskal-Wallis test.

The consensus matrix showed near-perfect separation, with most values close to 0 or 1 (Fig 3A). Item consensus probabilities exhibited two distinct peaks at 0 and 1 for cluster 1 and 2, respectively, confirming clear cluster delineation (Fig 3B). Cluster stability remained high across iterations, as indicated by consistently elevated adjusted mutual information (AMI) scores (Fig 3C).

Differences in feature values between IMOC clusters were also observed, with patients in cluster 2 having overall higher rates of CNA ($p=9.84\times 10^{-18}$) and higher values for methylation beta values ($p=2.64\times 10^{-15}$) (Fig 3D–3E).

### Patients in cluster 2 show a higher tumor mutational burden

**Fig 4:**
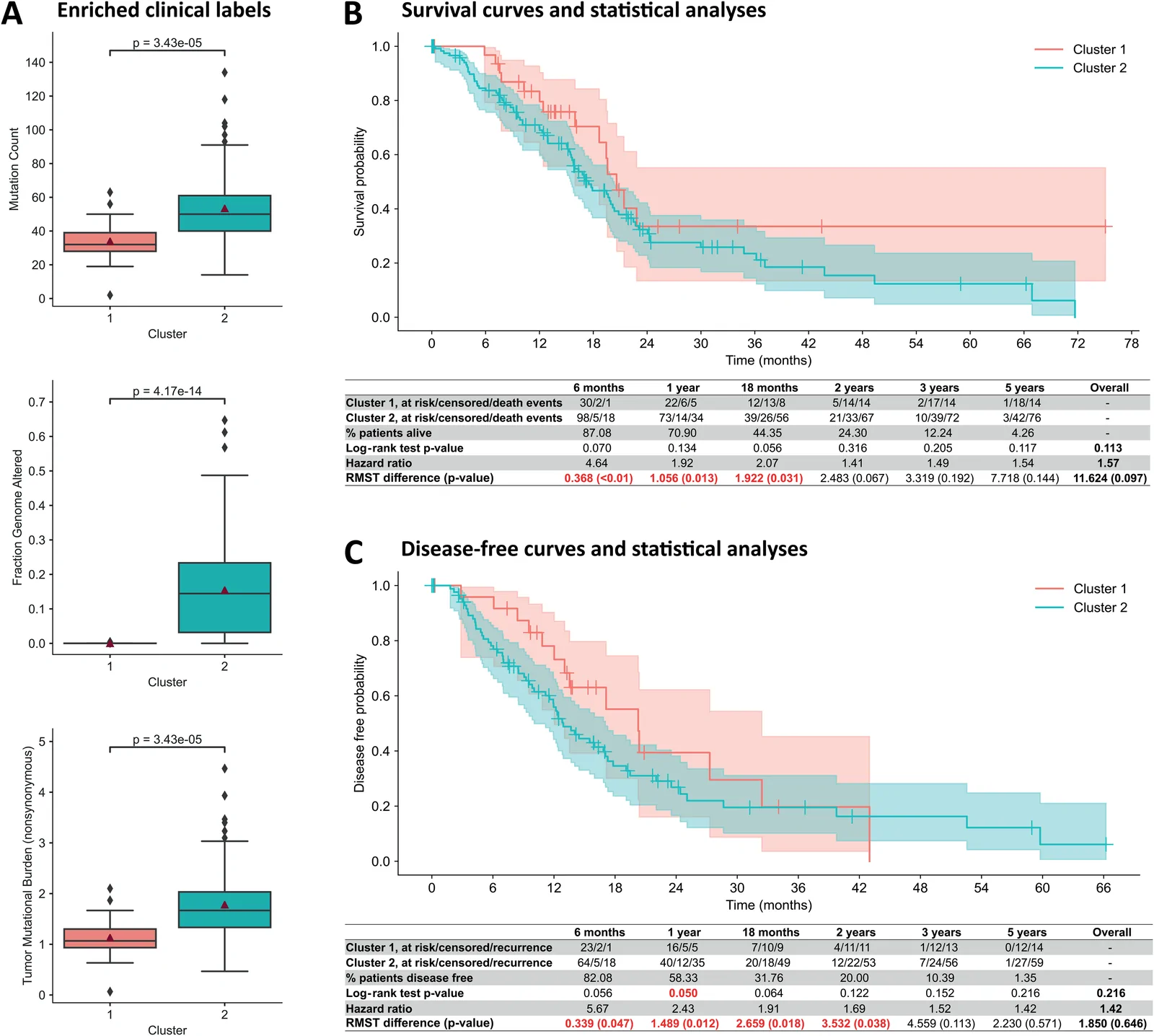
Clinical relevance of IMOC clusters. **(A)** Three clinical labels were significantly enriched in the clusters: mutation count, fraction of the genome altered and tumor mutational burden. P-values were calculated using the Kruskal-Wallis test and adjusted for multiple tests with the Benjamini-Hochberg correction. **(B, C)** Significant differences between the clusters in both, overall survival (B) and disease-free progression **(C)**, were observed during the first 18 months and two years post initial diagnosis, respectively. RMST: restricted mean survival time (difference in months in Cluster 1 respective to Cluster 2).

We next analyzed the differences in clinical variables between patients in cluster 1 and cluster 2. In the post-hoc enrichment analysis of clinical parameters (S2 Table), statistically significant differences were found in three parameters, all related to mutations: mutation count ($p=3.17\times 10^{-5}$), fraction of the genome altered ($p=3.86\times 10^{-14}$), and tumor mutational burden (TMB) ($p=3.17\times 10^{-5}$). Patients from cluster 2 had higher mutation count and TMB, as well as a larger fraction of the genome altered, indicating differences in somatic mutation profiles (Fig 4A).

### Differences in survival and disease-free progression within the first two-years post-diagnosis

Survival was recorded in the TCGA data for up to 6.5 years after initial diagnosis. For the survival data, the proportional hazards assumption was met (*p* = 0.74), whereas the random censoring assumption was not (*p* = 0.023). Across the study period, restricted mean survival time (RMST) analysis indicated that patients in cluster 1 lived, on average, one year longer than those in cluster 2 (Fig 4B). Statistically significant differences in the overall survival were found up to 18 months after the initial diagnosis, with patients in cluster 2 having lower survival probabilities. At 6 months, cluster 2 patients had over a 4.5-fold higher mortality risk than those in cluster 1, decreasing to about twice the risk by 18 months.

We next studied the tumor recurrence in all the patients that had disease-free progression information available (*n* = 113). In this case the assumption for censoring was met (*p* = 0.194), while the proportional hazards assumption was not (*p* = 0.035). Differences in disease-free progression remained statistically significant up to two years post-diagnosis, with patients in cluster 2 having higher recurrence rates (Fig 4C). At 6 months, the recurrence risk for cluster 2 patients was over 5.5 times higher, decreasing to approximately twice the risk at 2 years. RMST analysis indicated cluster 1 patients remained recurrence-free approximately 3.5 months longer at the two-year mark.

### Identification of biomarkers for patient stratification

**Fig 5:**
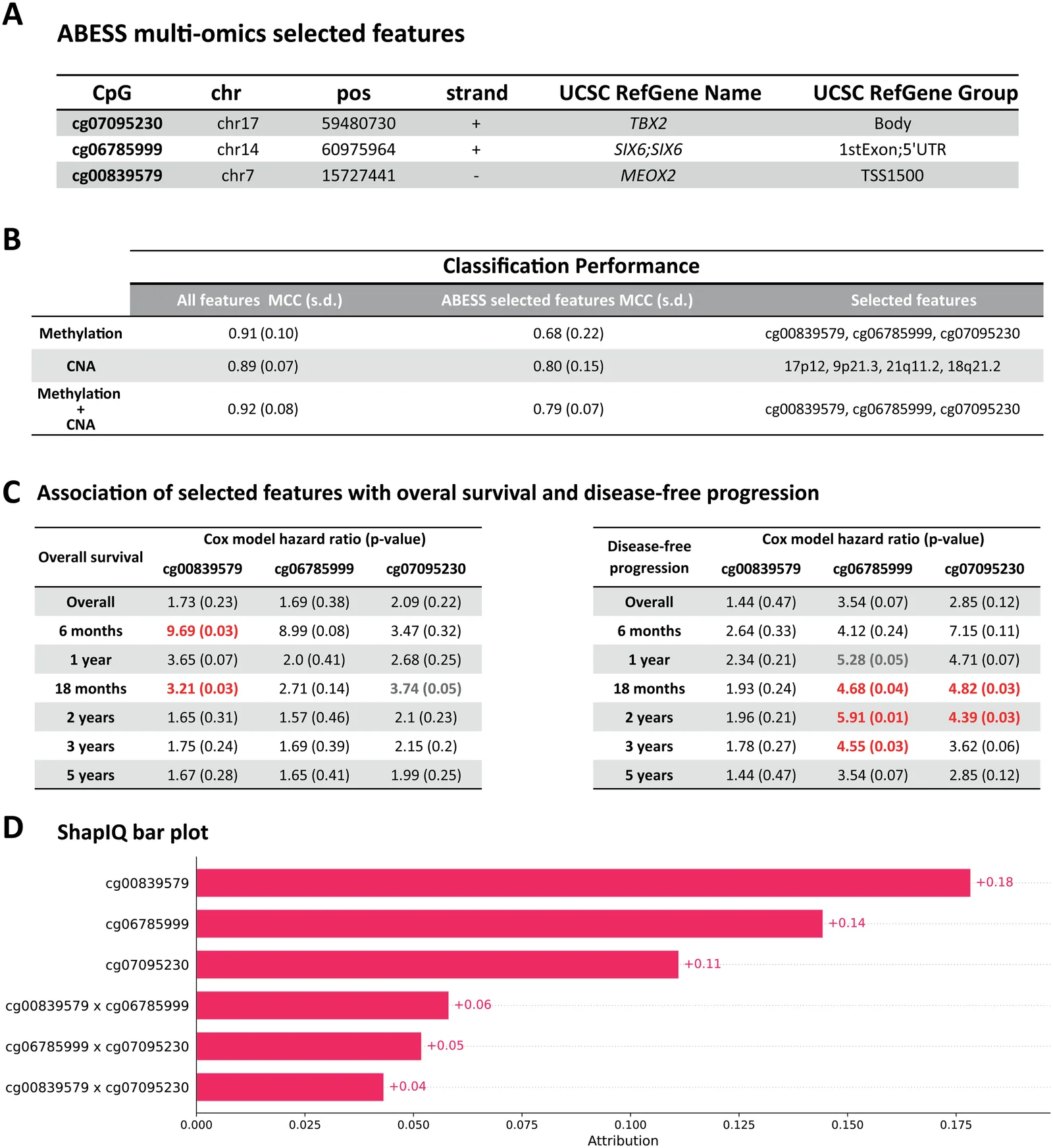
Statistical analysis of multi-modal selected features. **(A)** Feature selection was performed using a multi-modal approach, applying the ABESS algorithm with an intermediate fusion strategy. The table shows information about the selected features, all of which were methylation CpG biomarkers, including the chromosome (chr), position (pos), and the associated genes. **(B)** Classification performance (average of the 5-fold stratified cross-validation) of a random forest classifier for both, the full feature set and the selected features. The three selected methylation biomarkers were sufficient to stratify patients into the IMOC clusters with high accuracy. **(C)** The selected CpG features were associated with overall survival and disease-free progression at several time points post initial diagnosis, highlighting their clinical relevance. **(D)** ShapIQ assessment of feature and feature interaction influence. The three selected CpGs were identified as the most influential features. Feature interactions showed low importance, possibly reflecting the effectiveness of the selection, which isolated their individual effects. MCC: Matthews correlation coefficient. s.d.: standard deviation.

The multi-modal feature selection with Adaptive BEst Subset Selection (ABESS) [10] identified three methylation sites as potential biomarkers: cg00839579, cg06785999 and cg07095230, which were associated to the genes *MEOX2*, *SIX6*, and *TBX2*, respectively (Fig 5A). The features selected achieved strong classification performance, with an Matthews correlation coefficient (MCC) score of 0.79, compared to 0.92 obtained when all methylation and CNA features were used (Fig 5B). The feature selection applied individually to the CNA data resulted in the selection of four CNA corresponding to the 9p21.3, 17p12, 18q21.2, and 21q11.2 cytobands, while for the methylation data, it resulted in the same CpGs identified in the multi-modal feature selection.

The Cox model results using selected features for overall survival were inconclusive, with irregular significance across timepoints (Fig 5C and S1 Data). The disease-free analysis showed a more consistent associations with cg07095230, and especially with cg06785999, having a statistitically significant association from 1 to 3 years (Fig 5B). Within this timeframe, patients with hypermethylation of cg06785999 have around 5 times higher risk of tumor recurrence compared to those with normal methylation of this site. However, no significant association was found between the disease-free progression and any of the selected CNA (S1 Data).

Shapley Interaction Quantification (ShapIQ) identified cg00839579 as the most influential variable (Fig 5D). Interestingly, feature interactions showed low importance, possibly reflecting the effectiveness of the selection, which isolated their individual effects.

### Biological characterization of the IMOC clusters

**Fig 6:**
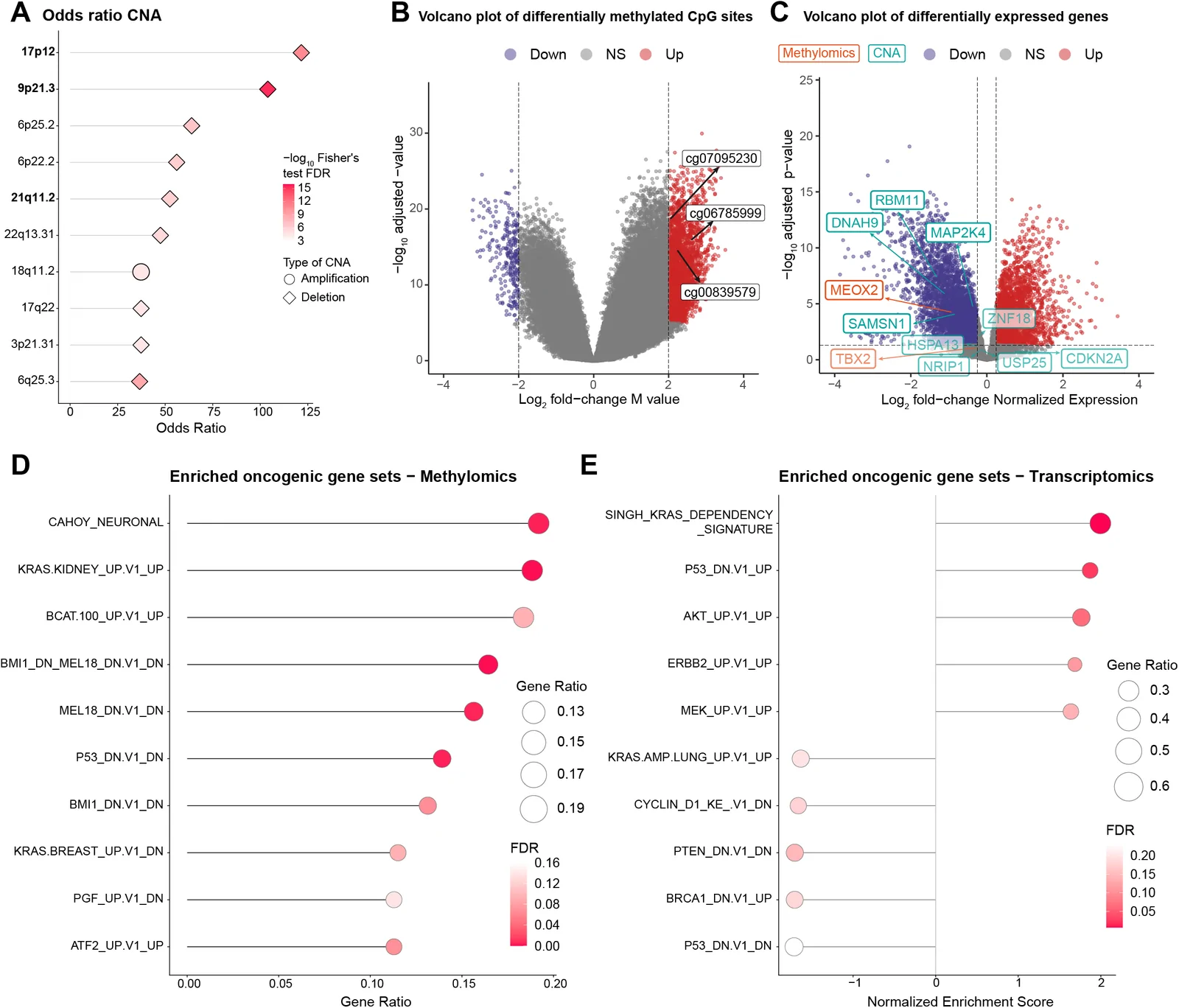
Multi-omics analysis of biological differences in cluster 2 vs cluster 1. **(A)** Three (bolded) of the four copy number features selected via unimodal feature selection were among the top 10 cytobands with the highest odds ratios for CNA events. The first two, also identified as biomarkers, showed a markedly larger difference than the others. **(B)** Volcano plot depicting the differential methylation analysis of CpG sites. Highlighted are the three CpGs identified in the biomarker selection. **(C)** Volcano plot showing the differential gene expression analysis. Indicated are the genes associated with the CNA or CpGs identified in the biomarker selection. **(D)** Dot plot showing the results from gene set enrichment analysis (GSEA) of methylomics data. The top 10 pathways with higher gene ratio (proportion of significantly enriched genes to the total number of genes in the gene set) and FDR < 0.25 are shown. **(E)** Dot plot showing the results from the GSEA of transcriptomics data. The top 10 pathways with higher absolute value of normalized enrichment score (NES) and FDR < 0.25 are shown. Key pathways in PDAC, including KRAS and P53, were among the most enriched in (D) and **(F)**. The color of the dots indicate the value of the FDR. The horizontal dashed line in (B) and (C) indicate the Benjamini-Hochberg adjusted p-value < 0.05 threshold to assess significance. NS: not significant.

After identification of the two distinct patient clusters using integrated DNA methylation and CNA data, we next aimed to characterize the biological differences between these groups of patients through comprehensive multi-omics analysis. We initially focused on DNA methylation and CNA, as these were the combined omic modalities selected for clustering. To evaluate the association strength between CNA in genomic regions (cytobands) and IMOC cluster classification, we calculated odds ratios for each cytoband (Fig 6A and S1 Appendix). The majority of CNA showing significant differential proportions between cluster 1 and cluster 2 patients were deletions (Figs 6A and S7 Fig). Notably, two of the four selected cytobands (17p12 and 9p21.3) demonstrated the strongest association with deletion events in cluster 2 patients. Differential methylation analysis comparing cluster 2 versus cluster 1 patients revealed 2773 CpG sites with significantly increased methylation in cluster 2, while 270 CpG sites were hypomethylated in cluster 2 (available in the results folder of the IMOC repository). Notably, all three CpGs identified as potential biomarkers were significantly hypermethylated in cluster 2 (Fig 6B). This hypermethylation pattern suggests distinct epigenetic regulation between the two IMOC patient clusters.

To investigate whether the observed alterations could be associated with changes in gene expression, we performed differential gene expression analysis (Fig 6C). We identified 3183 and 3966 differentially expressed genes (DEGs) with significantly increased and decreased expression in cluster 2, respectively (S2 Data). Consistent with the established inverse relationship between DNA hypermethylation and gene expression, we observed significantly decreased expression of *MEOX2*, which showed a significant negative correlation with methylation levels of its associated CpG site (cg00839579, S7 Fig). Similarly, *TBX2* expression demonstrated a significant negative correlation with its associated CpG (cg07095230) methylation (S7 Fig). While *TBX2* expression showed a trend towards reduced expression in cluster 2 patients, this difference did not reach statistical significance (Fig 6C). The gene *SIX6*, associated with the third selected CpG (cg06785999), was not detected in the gene expression dataset. Most of the CNA observed affected only one of the alleles (S7 Fig), suggesting that even when a deletion was present, the genes could still be expressed by the other allele. To assess whether the observed chromosomal deletions translate into functional consequences, we evaluated the expression levels of genes located within the four selected cytobands. Among the 95 genes located within the selected cytobands (S1 Data and S7 Fig), 9 were identified in the gene expression dataset. Of these, 4 genes showed significantly reduced expression in cluster 2 patients (Fig 6C), suggesting that the identified chromosomal deletions might have functional consequences on gene expression levels.

To gain insights into the biological consequences of altered methylation and gene expression patterns, we performed gene set enrichment analysis (GSEA) using both methylomics and transcriptomics data (Fig 6D–6E). Both omic modalities revealed significant enrichment in multiple oncogenic gene sets involving KRAS signaling pathways (S2 Data). Particularly noteworthy was the positive enrichment of genes defining the KRAS dependency signature in cluster 2 (Fig 6E), suggesting altered KRAS pathway activity in this subgroup of patients. Finally, given the importance of protein expression and phosphorylation in the final biological outcome, we performed a differential regulation analysis of the RPPA data, encompassing both protein expression and phosphorylation levels. We identified 13 proteins with increased expression and 11 proteins with reduced expression or phosphorylation in cluster 2 (S7 Fig). However, only Cyclin B1, CD49b, INPP4B, and GAPDH showed a significant statistical difference after adjusting for multiple comparisons (S2 Data). Of particular interest was the reduced phosphorylation observed in several proteins involved in the mTOR signaling pathway, including decreased phosphorylation of AKT, mTOR, and S6 ribosomal protein (S7 Fig). This pattern strongly indicates downregulation of mTOR signaling in cluster 2 patients.

Over-representation analysis of KEGG pathways using the differentially expressed and phosphorylated protein data revealed enrichment in several cancer-relevant pathways, including the HIF-1 signaling pathway, EGFR tyrosine kinase inhibitor resistance, or the p53 signaling pathway (S7 Fig and S2 Data). These findings further support the distinct molecular phenotypes of the IMOC clusters.

### IMOC clusters differs from previously reported PDAC subtypes

**Fig 7:**
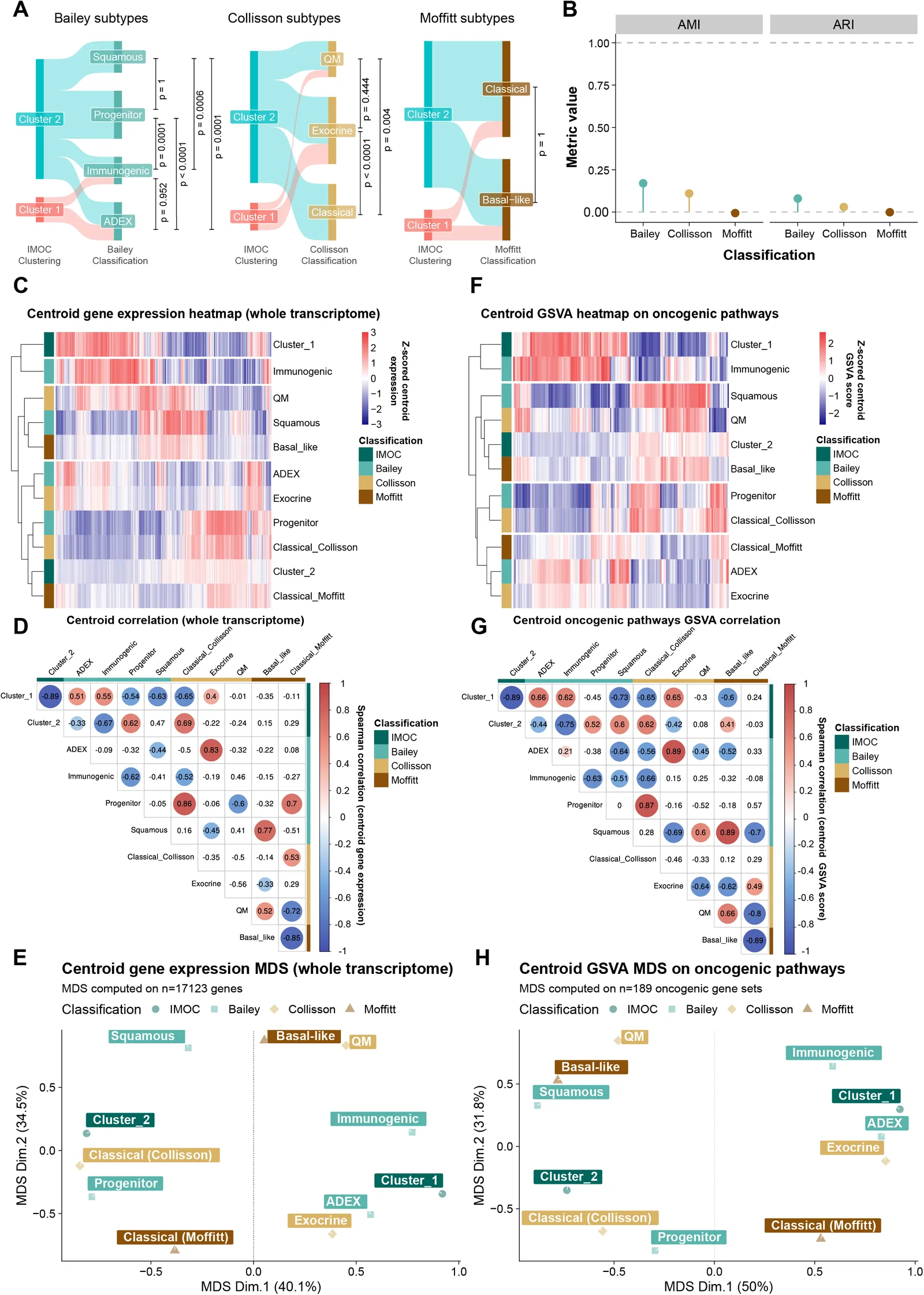
IMOC clusters show limited overlap with classic PDAC subtype labels. **(A)** Alluvial plots showing correspondence between IMOC cluster assignments and established PDAC subtypes (Bailey, Collisson, Moffitt). Pairwise differences between subgroups were calculated using Fisher’s exact test and p-values were adjusted for multiple comparison testing using the Benjamini-Hochberg correction. **(B)** Quantitative concordance between IMOC cluster labels and classic PDAC subtype taxonomies measured using adjusted mutual information (AMI) and adjusted Rand index (ARI). Both metrics are corrected for agreement expected by chance; values close to 0 indicate agreement no better than expected by chance, while values close to 1 indicate full agreement. **(C)** Heatmap of centroid gene-expression profiles computed across the whole transcriptome (*n* = 17,123 genes). Centroids were calculated as the mean normalized expression within each subtype/cluster. For visualization, expression values were z-scored per gene across centroids and hierarchically clustered using UPGMA agglomeration method. **(C)** Pairwise Spearman correlations between centroid gene-expression profiles. Dot size and color indicate the magnitude and direction of Spearman correlation; only correlations significant after multiple-testing correction (FDR < 0.05) have a dot shown. The numbers inside the cells indicate the value of the Spearman correlations. **(D)** Multidimensional scaling (MDS) of centroid gene-expression profiles in whole-transcriptome space (*n* = 17,123 genes). **(E)** Heatmap of centroid pathway activity profiles based on GSVA scores for oncogenic gene sets (MSigDB C6; *n* = 189). GSVA scores were z-scored per gene set across centroids for visualization. **(F)** Pairwise Spearman correlations between centroid GSVA profiles (oncogenic gene sets); only significant correlations after correction (FDR < 0.05) have a dot shown. The numbers inside the cells indicate the value of the Spearman correlations. **(G)** MDS of centroid GSVA profiles across oncogenic gene sets (*n* = 189).

The IMOC clusters discovered in this study were compared to the three most widely established clustering studies for PDAC patient stratification (Fig 7A–7B).

Bailey *et al.* used gene expression profiles to identify four subtypes of PDAC patients that correlate with histopathological features: aberrantly differentiated endocrine exocrine (ADEX), immunogenic, pancreatic progenitor, and squamous subtype [4]. Patients from cluster 1 were divided between the ADEX and immunogenic groups (*p* = 0.95), while patients in cluster 2 were distributed across all subtypes.

Collisson *et al.* presented a 3-subtype classification - classical, exocrine, and quasi-mesenchymal (QM) - of PDAC patients [5]. Patients belonging to cluster 1 were distributed between the exocrine and QM subtypes in Collisson’s classification (*p* = 0.44), while patients in cluster 2 were again found distributed across all subtypes.

Moffitt *et al.* defined a two-subtype classification (basal-like and classical) [6]. The patients in the IMOC clusters were evenly distributed across these two subtypes, with no enrichment between either clusters and the Moffitt’s subtypes (*p* = 1).

The alluvial comparisons indicated that neither IMOC cluster maps cleanly onto a single Bailey, Collisson, or Moffitt subtype, consistent with incomplete correspondence between the IMOC stratification and classic derived taxonomies (Fig 7A). To quantify concordance between IMOC clusters and published PDAC subtype taxonomies, we computed the AMI and ARI between labelings (Fig 7B). Concordance was low for all three taxonomies (Bailey: AMI = 0.17, ARI = 0.08; Collisson: AMI = 0.11, ARI = 0.03; Moffitt: AMI≈0, ARI≈0), indicating that IMOC clustering shows very limited agreement to previously defined subtype classifications and is not explained by a simple relabeling of existing groups.

Centroid expression heatmaps and centroid–centroid correlations computed across the whole transcriptome (*n* = 17,123 genes) revealed strong differentiation between the IMOC clusters, with cluster 1 and cluster 2 showing marked anti-correlation at the centroid level (Fig 7C–7D). In multidimensional scaling (MDS) embeddings of centroid profiles, IMOC clusters occupied distinct positions relative to classic subtype centroids, supporting that the IMOC-derived transcriptional axis is not equivalent to any single published subtype label (Fig 7E).

Pathway-centric analyses based on gene set variation analysis (GSVA) [11] of oncogenic gene sets recapitulated the separation observed in expression space. Centroid GSVA profiles and correlation structure again differentiated cluster 1 from cluster 2 and positioned IMOC centroids apart from classic subtype centroids in MDS space (Fig 7F, 7G, and 7H, and S3 Data), indicating that the IMOC clusters capture coherent differences in oncogenic pathway activity.

To further interrogate the relationship between IMOC clusters and classic subtype contrasts, we compared centroid profiles within a DEG-union feature space (*n* = 11470 genes, S2 and S4 Data). The DEG overlap structure showed that the IMOC cluster contrast contributed the largest intersection among DEG sets and included 707 DEGs unique to cluster 2 vs cluster 1 that were not shared with any classic subtype-derived DEG set (S8 Fig). Consistently, centroid correlations and MDS embeddings computed in DEG-union space emphasized strong separation of IMOC clusters relative to classic subtype centroids (S8 Fig).

We then explored whether there were differences in oncogenic gene set enrichment in the IMOC clusters versus the subtypes from other PDAC classifications. For each subtype of the classical PDAC taxonomies, we performed one-versus-rest GSEA to identify enriched oncogenic gene sets for each subtype (S5 Data). We then filtered the enriched oncogenic gene sets with FDR < 0.25 in each subtype to generate a restricted set of enriched oncogenic gene sets (*n* = 27), to directly compare the enrichment landscapes across contrasts. The IMOC cluster contrast showed the largest number of enriched oncogenic gene sets compared with any individual classic subtype contrast (S8 Fig). Importantly, there were several subtypes in Bailey, Collisson, and Moffitt classifications for which no significant enrichment at FDR < 0.25 was found (S8 Fig). Centroid GSVA profiles in this enrichment-restricted space remained strongly differentiated between the IMOC clusters (S8 Fig).

To test whether IMOC clusters capture oncogenic pathway activity beyond that explained by established PDAC subtype taxonomies, we modeled GSVA oncogenic gene-set scores as a function of cluster membership while adjusting for Bailey, Collisson, or Moffitt labels in separate limma frameworks (S9 Fig and S6 Data). Across all three adjustments, multiple gene sets remained significantly associated with IMOC cluster status, indicating that the IMOC-defined stratification reflects pathway-level biology not reducible to classic subtype labels. Notably, the KRAS dependency signature (SINGH_KRAS_DEPENDENCY_SIGNATURE) kept consistently showing a higher activity in cluster 2 relative to cluster 1 after adjustment for each taxonomy, alongside persistent differences in p53-related signaling (P53_DN.V1_UP) and AKT/MEK-linked oncogenic programs (AKT_UP.V1_UP; MEK_UP.V1_UP).

We also evaluated how the overall survival and disease-free progression differences between the IMOC clusters compared to the differences between subtypes from the other PDAC classifications. The analysis was restricted to patients with all four classifications (IMOC, Bailey, Collisson, Moﬀitt) available (n = 133 for overall survival, n = 100 for disease-free progression). The overall survival curves and disease-free progression curves showed that all four classifications produced visually relevant separation in Kaplan-Meier curves S10 Fig). Only the IMOC clusters exhibited non-intersecting overall survival trajectories. In contrast, the overall survival curves corresponding to all the other classifications crossed at multiple time points, suggesting less stable patient stratification. The curve intersections evidenced violation of the proportional hazards assumption.

Because the proportional hazards assumption is violated across all the four classifications, we computed RMST differences between the worst- and best-prognosis subgroups within each classification, at the same landmarks. RMST is robust to proportional hazard violations and reflects the area between survival curves up to a pre-specified time point τ. For multi-level taxonomies, we compared each non-extreme subtype to the worst-prognosis reference at τ=12 (Squamous for Bailey; QM for Collisson).

The RMST extreme-subtype contrasts showed that IMOC’s RMST difference is comparable in magnitude to the strongest pairwise contrasts within multi-level taxonomies (Bailey Immunogenic-vs-Squamous, Moffitt Classical-vs-Basal-like) across all time points (S11A Fig and S7 Data). To compare the precision-adjusted prognostic signal across classifications at each landmark, we computed the Wald z-statistic ($\hat{\Delta RMST}/SE(\hat{\Delta RMST})$) from RMST inference [12]. This statistic decomposes prognostic comparison into effect magnitude relative to estimation uncertainty, allowing direct comparison of binary classifiers (IMOC, Moffitt) against extreme-subtype contrasts within multi-level taxonomies (Bailey, Collisson). Importantly, the z-statistic comparison (S11B Fig) shows that IMOC achieves the highest signal-to-noise ratio for overall survival at τ=6 (z≈3.6) and τ=12 (z≈3.4), exceeding all three established taxonomies and any extreme-subtype contrast within them. This early-overall survival precision advantage reflects two structural features of IMOC. First, its binary nature, which applies the stratification to the full cohort rather than relying on extreme-subtype contrasts within larger taxonomies. And secondly, its derivation from a systematic benchmark across omics modalities, algorithms, and cluster numbers, which yielded stable cluster assignments robust to the modest event counts at early landmarks. This is especially relevant for PDAC, since most tumor recurrences and associated mortality occur during the first 2 years post-diagnosis [2].

Finally, we performed a RMST comparison stratified by subtypes, to evaluate whether IMOC adds RMST-defined prognostic separation within each of the classical subtypes. We limited this analysis to the Moffitt classification because it was the only classification in which each subtype (Classical and Basal-like) contained patients assigned to both IMOC Cluster 1 and IMOC Cluster 2 (Fig 7A). In contrast, the Bailey and Collisson classifications showed little overlap between several subtypes and the IMOC clusters, precluding meaningful within-subtype survival comparisons. The stratified RMST analysis (S12 Fig and S8 Data) revealed distinct temporal patterns of IMOC prognostic separation within each Moffit subtype. For overall survival, IMOC clusters separated significantly within Moffitt-basal-like patients at early landmarks (ΔRMST=0.5 months at τ=6, BH adjusted p-value = 0.005; ΔRMST=1.9 months at τ=12, BH adjusted p-value = 0.033), while within Moffitt-classical patients, separation became significant from τ=12 onward and remained significant through τ=36 (ΔRMST=3.9 months at τ=24, BH adjusted p-value = 0.01). For disease-free progression, IMOC produced significant separation only within Moffitt-classical patients (ΔRMST=4.8 months at τ=24, BH adjusted p-value = 0.02), with no significant separation within Moffitt-basal-like at any τ (S12 Fig). This pattern indicates that IMOC adds prognostic information beyond the binary Moffitt classification in two complementary ways: refining early overall survival stratification within Basal-like patients (where most early mortality occurs) and refining longer-term overall survival and disease-free outcomes within Classical patients.

Collectively, these analyses support that IMOC clustering captures a distinct, multi-gene transcriptional program and pathway activation pattern that is not encompassed by Bailey-, Collisson-, or Moffitt-defined subtypes. IMOC equals the global prognostic discrimination relative to established expression-based PDAC taxonomies, and it provides complementary information in specific contexts: early overall survival stratification (concentrated within Moﬀitt-Basal-like patients) and refined longer-term stratification within Moﬀitt-Classical patients. Together, these features support IMOC as a methodologically robust and clinically deployable stratification that captures complementary prognostic and molecular information alongside the established PDAC taxonomies.

### Validation using the CPTAC cohort

**Fig 8:**
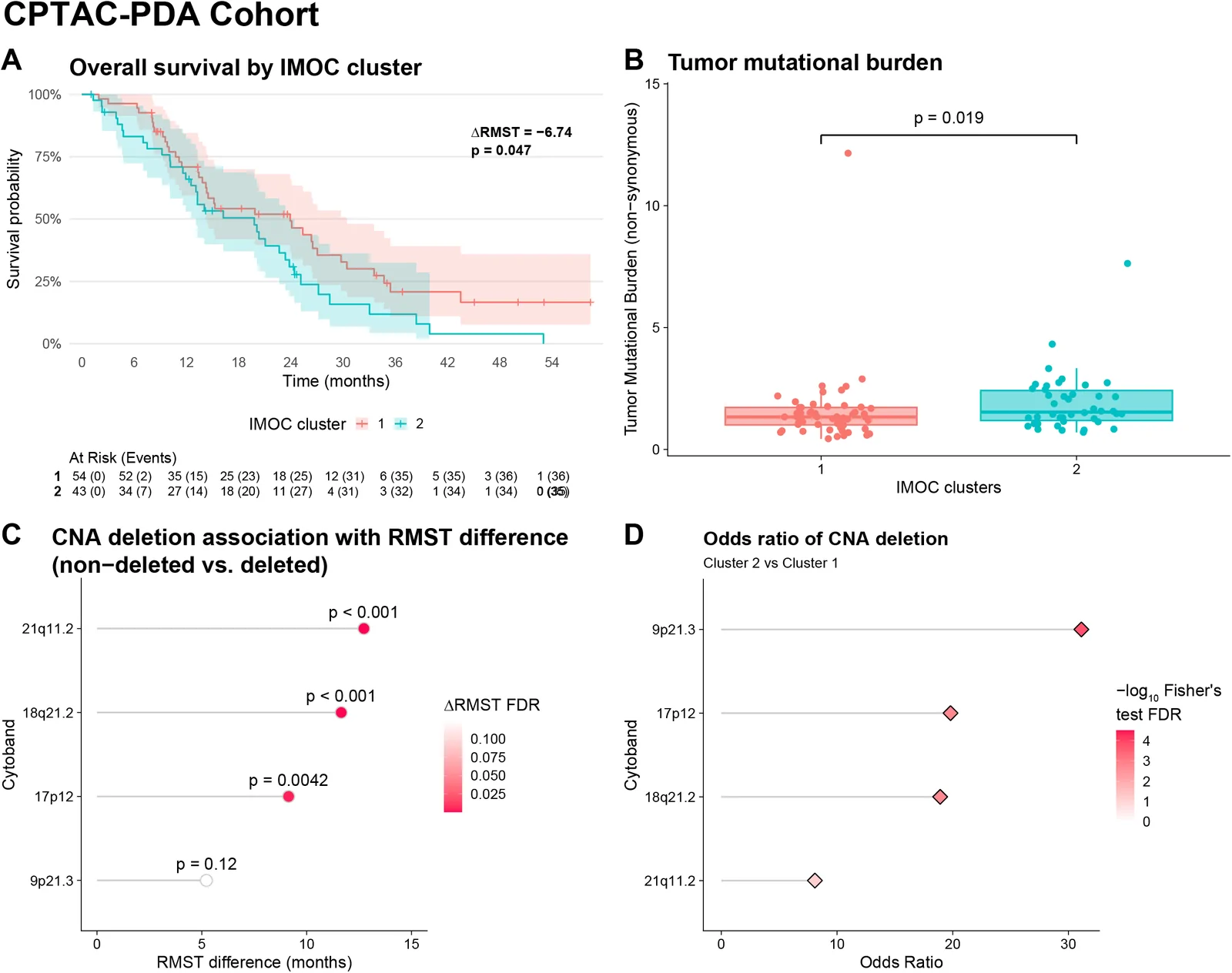
Validation of IMOC clusters and biomarkers using a CNA-based stratification model in the independent CPTAC-PDA cohort. **(A)** Cluster 2 (high-risk) exhibited significantly poorer survival compared to Cluster 1 (low-risk), with a RMST difference of 6.74 months (*p* = 0.047) **(B)** The high-risk Cluster 2 showed significantly elevated TMB compared to Cluster 1 (*p* = 0.019) **(C)** Association between specific cytoband deletions and RMST. Three of the four stratification features (18q21.2, 17p12, and 21q11.2) demonstrated significant associations with reduced survival (RMST difference ranging from 5 to 13 months; *p* < 0.05), while 9p21.3 was non-significant (*p* = 0.1245) **(D)** Odds ratio for the four copy number features used for the stratification. The high-risk phenotype is strongly associated with the presence of these chromosomal losses.

To assess the generalizability of our IMOC framework, we used the Clinical Proteomic Tumor Analysis Consortium (CPTAC-PDA) cohort for validation. We applied our CNA-based stratification model to the CPTAC data to assign samples to IMOC-like clusters. This analysis revealed significant differences in overall survival between the two groups, with Cluster 1 exhibiting a better prognosis compared to Cluster 2 (*p* = 0.047, ΔRMST = 6.74 months) (Fig 8A).

Consistent with our findings in the TCGA-PAAD cohort, the higher-risk cluster in CPTAC demonstrated significantly elevated TMB (*p* = 0.019) (Fig 8B). Furthermore, the chromosomal alterations identified as key drivers of subtype definition in our discovery cohort, specifically deletions at 9p21.3, 17p12, 18q21.2, and 21q11.2, retained their prognostic relevance in the independent CPTAC dataset. The frequency of these losses was significantly higher in the high-risk cluster, and three of the four regions (18q21.2, 17p12, and 21q11.2) showed statistically significant associations with reduced RMST ranging from 5 to 13 months difference (Fig 8C-8D).

## Discussion

This study aimed to find novel subtypes of PDAC patients using incomplete multi-omics data from TCGA. After benchmarking multiple clustering algorithms with different combinations of omics modalities, NEMO was applied on methylation and CNA data, identifying two clusters. Patients in cluster 2 exhibited statistically lower survival rates in the first 18 months after initial diagnosis and higher tumor recurrence up to 2 years after surgery, as well as higher mutational burden.

Clustering is an unsupervised task and, as is common in this domain, its evaluation is inherently challenging. Multiple scoring metrics have been proposed, each capturing a different aspect of cluster quality. Relying on a single score, however, may introduce limitations. To address this, we combined two key aspects of clustering quality, compactness and stability, using their harmonic mean. The harmonic mean has been widely applied in supervised settings, most notably in the F1-score, which integrates precision and recall. More recently, it has also been proposed as a general performance score for supervised classification, with the aim of obtaining the benefits of multiple metrics while reducing their individual limitations [13]. Inspired by this principle, we proposed a general performance score for clustering that leverages the strengths of multiple metrics while mitigating their individual limitations. The high-quality clusters obtained in our study support the applicability of this approach in future analyses.

The combination of DNA methylation and CNA data yielded the best clustering solutions across various algorithms and numbers of clusters. Among all modalities, methylation data turned out to be the most informative. This observation is consistent with several studies that have highlighted the integral role of DNA methylation in prognosis of PDAC patients [14,15]. DNA methylation is a key epigenetic mechanism regulating gene transcription and influencing fundamental biological processes, including cellular differentiation, tumorigenesis, and immune response regulation, with particularly profound implications in cancer. Aberrant promoter hypermethylation, in particular, can inactivate tumor suppressor genes, thereby contributing to tumorigenesis and disease progression in PDAC [16]. In contrast, while early studies reported no significant association between copy number alterations and PDAC tumorigenesis and progression, more recent evidence has demonstrated CNA involvement in both sporadic and familial pancreatic cancers. These findings established CNA as one of the main contributors to the aggressive biology of PDAC [17].

Several studies have researched the performance of multi-modal algorithms on multi-omics cancer data [18,19]. However, the performance of different modality combinations is rarely studied, and only a few have considered scenarios with incomplete data, despite this being the most common situation in biological research [8]. Although focused on PDAC, our study represents the most extensive comparison to date in incomplete multi-omics analysis.

When testing the algorithms on increasing levels of incomplete data, NEMO exhibited the most robust performance. As the rate of missing information increases, it is intuitive that the performance of algorithms decreases, since each modality carries less information [20]. However, it is desirable for algorithms to have high performance even with large amounts of missing data, especially in clinical settings, where a large fraction of the data collected can be incomplete. In these settings with incomplete data, NEMO has been recognized as a state-of-the-art algorithm for clustering of multi-omics data, often outperforming other algorithms [18], a conclusion corroborated by our study.

Nevertheless, a limitation of this work is its focus on missing modalities only. This reflects a broader limitation within the field, as most current approaches do not account for both feature- and modality-level missingness simultaneously. Developing robust multi-modal algorithms capable of handling both is essential, as such scenarios more accurately represent real-world data.

Our clustering analysis identified two distinct subtypes. IMOC clusters showed weak agreement with Bailey, Collisson, and Moffitt subtypes and remained distinct in centroid-based transcriptome and oncogenic pathway analyses. Moreover, key pathway differences—particularly KRAS dependency and p53/AKT–MEK programs—persisted after adjusting for each classic taxonomy, supporting that IMOC captures an orthogonal axis of PDAC heterogeneity rather than a relabeling of established subtypes. These findings suggest that the clusters identified here represent a novel stratification of PDAC patients.

Patients belonging to cluster 2 had higher TMB and lower survival rates. An elevated TMB has been established as a predictive biomarker of response to immune checkpoint inhibitors [21]. Consistently, recent studies have demonstrated that immunotherapy, an emerging treatment option for cancer, shows promising results in PDAC patients with high TMB [22]. These observations underscore the clinical relevance of our clusters.

Significant differences in both survival and disease-free progression between IMOC clusters were observed during the first 18 months and two years, respectively. These differences are likely no longer observed after the 2-year post-surgery mark due to 80% of patients developed tumor recurrence, and 76% have died, leaving only one and two patients remaining in cluster 1, respectively (Fig 4B–4C), which reduced the statistical power for detecting such differences. Additionally, the two survival curves nearly intersect at approximately 18 months in both analyses, violating the proportional hazards assumption underlying the log-rank test and Cox models [23]. To account for these deviations, differences in survival and disease-free progression should be measured at different time points, as implemented in our study [24]. While most previous studies have not accounted for this limitation and instead relied on the direct outputs of log-rank tests and Cox models, we saw that this step is essential, as it evidences the strong clinical relevance of the IMOC clusters.

The comparison of the survival and disease-free progression differences between IMOC clusters and the differences between subtypes from the other PDAC classifications showed that IMOC provided similar global prognostic discrimination relative to established expression-based PDAC taxonomies, providing complementary information in specific contexts, such as better early overall survival stratification (S11 and S12 Figs). This is consistent with IMOC’s derivation from a different molecular substrate (methylation + CNA) than the expression-based comparators, which were specifically engineered for prognostic and biological discrimination. Beyond global discrimination metrics, several features distinguish the IMOC stratification: (i) the RMST contrasts between IMOC clusters are competitive in magnitude with the strongest extreme-subtype contrasts within Bailey, Collisson, and Moffitt, while achieving the highest signal-to-noise ratio for early overall survival of any comparison tested; (ii) the IMOC clusters were derived from a systematic benchmark across six omics modalities, nine clustering algorithms, and multiple cluster numbers, with high consensus stability (Fig 3A–3C), a level of methodological scrutiny not performed by previous classifications; (iii) IMOC’s binary structure assigns every patient unambiguously to one of two clusters, in contrast to multi-level taxonomies whose strongest prognostic separations rely on extreme-subtype contrasts that exclude patients in intermediate subtypes; and (iv) the IMOC stratification can be reduced to a four-locus CNA biomarker panel that is inherently comparable across datasets and does not require preprocessing or batch correction, enabling direct application to new samples without the gene expression profiling and normalization required by Bailey, Collisson, or Moffitt classification.

Patients belonging to the poor-prognosis cluster exhibited global hypermethylation of CpG sites (p=2.64e−15) and higher overall CNA (p=9.84e−18) compared to the lower-risk group. The global hypermethylation landscape for patients in the higher-risk group aligns with the results from Fraunhoffer *et al.*, who identified global hypermethylation as a marker for poor prognosis in other PDAC patients cohorts [14]. According to Detlefsen *et al.*, a high overall copy number variation burden results in poor prognostic for surgically treated PDAC patients in a Danish cohort [25]. This aligns with the findings in this study, where patients in the high copy number burden group had lower survival expectancy as well as higher tumor recurrence rates.

We found that only three methylation biomarkers were sufficient to stratify patients into IMOC clusters. The CpG site cg07095230 has been reported as significantly differentially methylated in endometrial cancer, both in the TCGA cohort and in independent datasets [26]. Its associated gene, *TBX2*, has been linked to metastasis and poor survival in lung cancer [27]. Moreover, the antifungal piroctone olamine was identified as a *TBX2*-targeting compound in melanoma and rhabdomyosarcoma [28], and more recently shown to be effective in PDAC patient-derived organoids, highlighting its potential for repurposing in *TBX2*-dependent PDAC [29]. The CpG site cg06785999 lies within the regulatory region of the tumor suppressor gene *SIX6*. Aberrant hypermethylation of this gene has been proposed as the best epigenetic marker in lung cancer [30], and *SIX6* has also been associated with radioresistance in pancreatic cancer cell lines [31]. Importantly, hypermethylated *SIX6* has been proposed as a Universal Cancer Only Marker (UCOM) for early cancer screening, as well as for precancerous stage and metastasis emergence tracing [32]. The expression of *MEOX2* is highly regulated by methylation status in its promoter [33], where the CpG biomarker cg00839579 we found is located. This gene has been found to be very important in a side population resistant to gemcitabine in PDAC [34], representing a therapeutic target in pancreatic cancer. It has also been proposed as part of an angiogenic gene signature in PDAC [35].

The unimodal feature selection also identified four copy number deletions located in cytobands 9p21.3, 17p12, 18q21.2, and 21q11.2. Of these, 17p12 and 9p21.3 demonstrated the strongest association with deletion events in cluster 2 patients. 17p12 deletion is among the most common losses in PDAC [36], and it has been reported that genetic loss of *MAP2K4* gene located in this region occurs in 30% of PDAC cases [36]. In agreement with the increased association of 17p12 deletions with cluster 2, the expression of *MAP2K4* was also reduced in these patients (Fig 6C). *MAP2K4* encodes for MKK4 protein kinase, regulating the JNK and p38 stress activated protein kinases pathways, which have been involved in numerous types of cancer [37]. MKK4 has been proposed as a tumor suppressor protein in PDAC, and the loss of MKK4 is associated with poorer outcomes in several PDAC cohorts [38]. Notably, loss of 9p21.3 is a recurrent CNA across cancers, strongly associated with poor prognosis [39]. The genes included in the peak limits region of the 9p21.3 deletion included the *CDKN2A* gene, a central tumor suppressor gene involved in the activation of p53 tumor-supressor pathway. Consistent with previous work, deletions at 9p21.3 usually occur concurrently with *KRAS* activating mutations, another major driver of PDAC [40]. Although the overall expression of *CDKN2A* was not significantly altered in the gene expression data (Fig 6C), we found a significant enrichment in genes affected by p53 mutation and genes defining the KRAS dependency signature in cluster 2 (Fig 6E). Interestingly, all 9p21.3 CNA in our cohort were heterozygous (S7 Fig), in line with reports showing that *CDKN2A* deletions often begin as heterozygous events that later progress to loss of heterozygosity during tumor evolution [41]. Together, these findings suggest that cluster 2 patients may represent a high-risk group predisposed to complete *CDKN2A* inactivation, consistent with their poorer prognosis (Fig 4).

We have released the CNA-based PDAC patient stratification model in our repository to facilitate the stratification of new samples and clinical translation. The model uses only the four selected copy number loci, whose values are inherently comparable across datasets, allowing its straightforward application.

Our feature-selection procedure was designed to identify biomarkers that capture the underlying structure identified by the integrative clustering analysis. In this context, the goal was to determine which molecular features best represent the discovered subtypes, thereby facilitating biological interpretation and the development of simplified subtype-classification models. The clinical relevance of the identified biomarkers was shown in the independent CPTAC validation cohort, where the CNA-based classifier successfully recovered prognostically distinct patient groups (Fig 8). These results suggest that the selected biomarkers capture biologically meaningful subtype-defining signals that are associated with clinically relevant outcomes.

Although the biomarkers identified by our multi-modal feature selection have well-established connections to PDAC and cancer biology, a limitation of the present work is that our analysis does not directly address the regulatory mechanisms driving the differential expression of the identified biomarkers between IMOC clusters. Subtype-specific gene expression in cancer is increasingly understood to be shaped by variations in enhancer activity and chromatin accessibility, which in turn reflect underlying changes in epigenomic cellular state and transcription factor activity [42,43]. Recent work integrating ATAC-seq and H3K27ac ChIP-seq with transcriptomic data has demonstrated that machine learning approaches can identify modulated transcription factor activity pathways and enhancer-driven regulatory programs in gastric cancer [44,45], and that such directly causal regulators may serve as more mechanistically grounded biomarkers than gene expression measurements alone. The TCGA PDAC cohort used in this study does not include ATAC-seq or H3K27ac ChIP-seq data, precluding direct interrogation of enhancer activity and chromatin state in our IMOC clusters. Extending the IMOC framework to incorporate chromatin accessibility profiling, as such data become available in PDAC cohorts, represents a natural and important direction for future work, with the potential to identify the regulatory determinants underlying the differential methylation and copy number patterns we observed, and to refine the proposed biomarker panel toward causally upstream targets.

Although limited in coverage, the RPPA dataset provided valuable insights into molecular and pathway-level differences between clusters. We identified broad alterations in oncogenic signaling, with reduced mTOR pathway activation in cluster 2 (S7 Fig), in contrast to the hyperactivation commonly described in PDAC [46]. Since mTOR inhibition remains a therapeutic strategy for subsets of PDAC patients, this observation highlights possible treatment implications. Our results suggest that patients in cluster 1 could benefit from mTOR inhibition treatment, while the same treatment might not be effective in patients in cluster 2, given the already downregulated status of the pathway. In addition, KEGG pathways over-representation analysis revealed enrichment of signatures related to EGFR tyrosine kinase inhibitor resistance (S7 Fig), which is associated with aberrant KRAS pathway activity. Finally, the most significant differentially expressed protein identified was Cyclin B1, which showed an increased expression in cluster 2 (S7 Fig), and which has been previously described as a PDAC biomarker associated with shorter survival in PDAC patients [47], further supporting the aggressive phenotype of this group.

The molecular characterization of the IMOC clusters opens specific avenues for differential therapeutic strategies. Cluster 2 patients show enrichment of the KRAS dependency signature and reduced mTOR pathway activation, suggesting that emerging KRAS-targeted therapies may be particularly relevant for this group while mTOR inhibition, which retains therapeutic relevance for subsets of PDAC patients [46], may be more appropriate for cluster 1, where mTOR signaling remains intact. While prospective testing of these therapeutic hypotheses lies beyond the scope of the present work, the IMOC stratification provides a molecularly grounded framework within which such hypotheses can be tested in future studies.

The successful replication of survival stratification and biomarker associations in the independent CPTAC-PDA cohort underscores the robustness and clinical applicability of our IMOC framework. The observation that the high-risk cluster is characterized by both shorter survival and elevated TMB aligns with the findings from the IMOC clusters. Significantly, the preservation of the prognostic value of specific cytoband deletions (9p21.3, 17p12, 18q21.2, and 21q11.2) across two distinct cohorts suggests that these chromosomal alterations represent fundamental biological events rather than cohort-specific artifacts. The significant RMST differences and consistent TMB stratification confirm that the identified subtypes capture biologically distinct trajectories, even in the absence of strict proportional hazards. This external validation demonstrates that a CNA-driven approach can effectively stratify patients in independent datasets, supporting its potential utility as a reproducible tool for risk stratification in pancreatic ductal adenocarcinoma.

## Conclusion

Two novel subtypes of PDAC were found after exhaustive analysis of incomplete multi-omics data. The high-risk group, with higher TMB, showed lower survival and higher recurrence rates. Our integrative analysis revealed substantial molecular differences between the two IMOC clusters. The poor-prognosis group, in particular, is characterized by 17p12 and 9p21.3 deletions, higher Cyclin B1 expression, and dysregulated p53 and KRAS pathways, together with reduced mTOR activation, which could be especially relevant for treatment decision. These alterations provide a mechanistic rationale for the observed differences in survival outcomes and disease-free progression. The association of specific CNA and CpG biomarkers with these molecular phenotypes highlights their potential for risk stratification, prognosis, and treatment guidance. In conclusion, our results demonstrate that integrative multi-omics clustering yields biologically meaningful PDAC subtypes and identifies candidate biomarkers with potential utility in precision oncology.

## Materials and methods

### Dataset

Multi-omics data were downloaded from the Firehose Broad GDAC (https://gdac.broadinstitute.org/), using the R packages curatedTCGAData [48] and TCGAutils. Six types of omics data were downloaded: protein expression (Reverse Phase Protein Array, normalized expression values), micro-RNA (Illumina HiSeq, gene-level normalized RPM miRNA expression values), DNA methylation (Illumina Human Methylation 450, probe-level methylation beta values), gene expression (Illumina HiSeq, upper quartile normalized RSEM TPM gene expression values), mutations (somatic mutation calls), and somatic copy number alterations (GISTIC2 thresholded discrete copy number values in recurrent peak regions). There were a total of 154 patients, with each patient having information in at least one data type (S13 Fig). Preprocessing pipelines are described in S1 Appendix and S1 Table. Clinical data were downloaded from cBioPortal.

For the external validation, we downloaded CNA and methylation data from the CPTAC-PDA cohort using https://kb.linkedomics.org/download#PDAC [49]. Because CNA measurements in this cohort are provided at the gene level, whereas TCGA reports CNA at the chromosomal-region level, we harmonized the two datasets by aggregating gene-level measurements within each chromosomal region. This procedure showed a high concordance with the original data, with agreement ranging from 95% to 100% across the four prognostically relevant regions included in the model (0.95, 0.99, 1, 1 for 21q11.2, 17p12, 18q21.2 and 9p21.3, respectively).

### Overview of the benchmarking strategy

To identify robust multi-omics subtypes of PDAC, we implemented a three-stage benchmarking framework. First, all possible combinations of omic modalities and cluster numbers were evaluated across multiple clustering algorithms using patients with complete multi-omics profiles. Cluster quality was assessed based on both compactness and reproducibility. Second, the robustness of the clustering algorithms was benchmarked under increasing levels of simulated modality-wise missing data while keeping the previously selected modality combination and cluster number fixed. Third, the algorithm demonstrating the highest robustness was used to generate the final clustering solution in the full cohort through consensus clustering. The resulting subgroups were then compared with established PDAC molecular classification systems.

### Finding the best combination of modalities and optimal number of clusters

All combinations (57) of omic modalities were systematically evaluated, where each combination had at least two omic modalities present, plus the evaluation of single modalities (6). Only those patients containing information across all the data types (*n* = 89) were used at this first stage to reduce noise. Thus, at any degree of missing data, all experiments had the same number of samples, with the only variable being the distribution of the available data. This ensured results were not biased due to sample size for certain combinations.

Nine clustering algorithms (detailed in S1 Appendix), including two factorization-based methods [50,51], three kernel-based methods [52–54], and four graph-based methods [20,55–57] were tested for 2–5 clusters. These algorithms are available in the Python package iMML (https://github.com/ocbe-uio/imml) [58], and were selected due to their ease of use, scalability to high-dimensional data, and computational speed, reproducibility and performance in previous benchmarkings (software and pipelines are described in S1 Table). Additionally, hierarchical clustering and spectral clustering, two widely used and well-established methods, were used to provide appropriate single-modality baselines for comparison.

For our evaluation, we included methods that can handle bulk multi-omics data with an arbitrary number of modalities, do not require assumptions about specific missingness distributions, and provide a ready-to-use Python implementation, suitable for straightforward application by end users. Importantly, we did not include deep learning-based approaches since they generally require substantially larger training datasets than those usually available in survival datasets, as the ones in this study, to achieve reliable performance. The included multi-omics integration methods were therefore selected as established baselines that have been widely used in prior comparative studies, providing a meaningful reference for evaluating the proposed approaches, with MOFA [59] being the standard method for the integration of incomplete multi-omics data in recent studies [60–62].

To improve the reliability of the results, all experiments were tested with 10 independent repeats with subsets of 80% of patients (*n* = 71). To evaluate cluster quality, we assessed their compactness using the silhouette score and stability across runs through pairwise AMI (S1 Appendix). To obtain an unbiased view of both scores, we combined them using the harmonic mean, referred to as the general performance score (GPS), as was done in previous works [13]. Statistical differences in the GPS were analyzed using the Kruskal-Wallis test. All the results from the benchmarking are available in S9 Data.

### Determining the most robust clustering algorithm

The robustness, quantified as the patients assigned to the same cluster in both the complete and missing-data solutions, was measured comparing the cluster assignments from the solutions with no missing data as a reference using pairwise AMI. The clustering algorithms were tested on various simulated, modality-wise missing data patterns generated from patients with complete multi-omics profiles with 80% subsampling. Each scenario was tested at increasing levels of incomplete samples (20%, 40%, 60%, and 80%) and with 10 independent repeats. The results of the evaluation are available in S10 Data

### Obtaining the final clusters with consensus clustering

All the patients (*n* = 154) were used to identify the final clusters, provided that every patient had data from at least one of the omics from the best combination of the modalities. We employed consensus clustering to create the final clusters (S1 Appendix). The clusters identified were compared to previous works, focusing on the three most widely established clustering studies of PDAC patients: Bailey, Collisson and Moffitt subtypes [4–6]. Data was obtained from pdacR [63]. Pairwise differences between subgroups were calculated using Fisher’s exact test. The results with the consensus clustering evaluation are available in S11 Data.

### Statistical analysis of final clusters

Post-hoc statistical analyses of the clusters were performed using the patients’ clinical data. 25 clinical labels (see S2 Table) were tested for enrichment, including data about the patient and tumor, by applying the Kruskal-Wallis test for numerical variables and Chi-square test for categorical variables. P-values were adjusted for multiple tests using the Benjamini-Hochberg correction. The results of the statistical analysis of clinical labels is available in S11 Data.

Survival and disease-free progression differences between patient clusters were evaluated using Heinze’s conditional log-rank test, which provides greater robustness to group imbalance compared to traditional log-rank test [23]. Associations between cluster membership and survival outcomes were further assessed using the Cox proportional hazards model. Both approaches rely on two key assumptions: random censoring and proportional hazards. These assumptions were evaluated using a simple linear regression model and the proportional hazards test, respectively. When these assumptions were not satisfied, results from the log-rank test and Cox model were reported over time, as previously recommended [24]. Accordingly, survival metrics were assessed at 6 months, 1 year, 18 months, 2, 3 and 5 years after initial diagnosis, as well as at the end of the study. Particular emphasis was placed on the first 2 years post-diagnosis, as most tumor recurrences and associated mortality occur during this period [2].

RMST was also used to evaluate the differences between the survival and disease-free curves up to specified time points. This test is also recommended when the proportional hazards assumption is not met [24].

To compare clusters, feature values for each modality were summarized at the patient level by averaging across all features (absolute values in the case of CNA). Differences in these patient-level averages between clusters were then assessed using the Mann-Whitney U rank test.

### Biomarker identification and analysis

Biomarkers were identified using ABESS [10] with an intermediate fusion multi-modal framework, taking the cluster labels as predictive targets (S1 Appendix). For comparison, unimodal feature selection was also performed using ABESS. The MCC was calculated using 5-fold stratified cross-validation with a random forest classifier and balanced subsampling for both the full feature set and the feature subset to compare model performances. The Cox model was applied to the features selected to obtain the hazard ratio and p-value. ShapIQ was used to explain the influence of the features and quantify the synergy effect between them [64].

### Omics analysis

Differential and Gene Enrichment analyses of transcriptomics, methylomics, and proteomics data were performed using standardized workflows in R (v4.4.1) (S1 Appendix). For the evaluation of the association between CNA and the cluster groups, odds ratios were calculated from contingency tables and Fisher’s exact test was used to compare proportions in each cluster.

## Supporting information

S1 Appendix Supplementary information. Contains additional information on data preprocessing, algorithms, cluster evaluation, multi-modal feature selection and omics analyses.
(PDF)

S1 Data ABESS unimodal feature selection results for methylation and copy number. Additional information on selected CNA regions and corresponding genes, as well as results for Cox model at several time points for selected methylation and copy number, using overall survival and disease-free progression data.
(XLSX)

S2 Data Multi-omics analysis results. Additional information on CNA odds ratios for every cytoband, generalized oncogenic gene set testing results for methylation data, gene set enrichment analysis (GSEA) results for gene expression data, RPPA differential protein expression / phosphorylation analysis, and KEGG pathways enrichment analysis of differentially expressed / phosphorylated proteins.
(XLSX)

S3 Data Gene set variation analysis on oncogenic gene sets. (XLSX)

S4 Data Differential expression analysis by subtype within classification taxonomy using “One-vs-Rest”. Each tab contains the differential gene expression analysis using limma for each subtype vs the rest.
(XLSX)

S5 Data Gene set enrichment analysis (GSEA) by subtype within classification taxonomy using “One-vs-Rest”. Each tab contains the GSEA results for each subtype vs the rest.
(XLSX)

S6 Data Differential gene expression analysis (cluster 2 vs cluster 1) results after adjustment for Bailey, Collisson, or Moffitt labels. Each tab contains the limma results for the differential gene expression analysis adjusting by each classification labels.
(XLSX)

S7 Data Restricted mean survival time (RMST) differences between the worst- and best-prognosis subgroups within classifications. (XLSX)

S8 Data Restricted mean survival time (RMST) comparison between IMOC clusters stratified by Moffitt subtypes. (XLSX)

S9 Data Benchmarking of modality combinations, number of clusters, and algorithm robustness. Data for Figs 2A-2B, S1-S4.
(XLSX)

S10 Data Results from algorithm performance with increasing fractions of missing data. Data for Figs 2C, S5.
(XLSX)

S11 Data Results from IMOC clusters: consensus matrix (Fig 3A), stability results (Fig 3C), variations in copy number and methylation values between clusters (Fig 3D-3E), and enrichment of clinical labels (Fig 4A). (XLSX)

S1 Table Summary of preprocessing pipelines used for every omics modality and algorithm configurations. Table includes number of patients with available data for each modality, number of features before preprocessing (original), key preprocessing parameters, feature counts after preprocessing steps were completed, and configurations of each clustering algorithm.
(XLSX)

S2 Table Clinical variables. Data on clinical variables used for analysis, statistical tests used and adjusted p-values.
(XLSX)

S1 Fig Evaluation of clustering performance across the different combinations of omic modalities. The combination of methylation and somatic copy number alterations (CNA) was the most informative according to the silhouette score (A) and adjusted mutual information (AMI) score (B). Both metrics were normalized with respect to the combination of modalities.
(EPS)

S2 Fig Methylation was the most important modality. (A-C) Individual importance of modalities, according to the general performance score (GPS) (A), silhouette score (B), and AMI score (C). Both the silhouette and AMI scores were normalized. Each of the points in the plot indicates the score for a combination containing that modality. Modalities are ordered from highest to lowest mean score (green triangle). In all three cases, the most important modality was methylation, followed by miRNA.
(EPS)

S3 Fig Algorithm performance across combination of omic modalities. (A) General performance score (GPS). (B) Silhouette score. (C) AMI score. All metrics were normalized. Algorithms are ordered from highest to lowest mean score (green triangle). In all three cases, the algorithm with the highest overall performance was IMSR.
(EPS)

S4 Fig Clustering performance for 2–5 clusters. (B) Silhouette score. (C) AMI score. All metrics were normalized. The clustering score for each number of clusters is ordered from highest to lowest mean score (green triangle). For the silhouette score, 2-cluster classifications yielded the highest score, whilst 3-cluster solutions obtained the best results according to the AMI score. (C) Individual evaluation of optimal number of clusters for each algorithm. All algorithms performed better when the number of clusters was set to 2, except EEIMVC, which demonstrated a slight better performance for 3 clusters.
(EPS)

S5 Fig Evaluation of the performance of the models depending on the different missingness patterns used. In all cases, the performance of the models, evaluated by the (A) general performance score, (B) silhouette score, (C) AMI score, and (D) robustness, did not vary notably with missingness patterns. MCAR: missing completely at random, MEM: mutually exclusive missingness, PM: partial missingness, MNAR: missing not at random.
(EPS)

S6 Fig Comparison of imputation-based pipelines versus incomplete data handling using the methylation and copy number (CNA) modalities. The analysis employed the NEMO algorithm with two clusters. Comparison of (A) robustness and (B) general performance score across varying percentages of missing data. Robustness was quantified by comparing clusters obtained with missing data to those found in complete data using the adjusted mutual information score. Imputation-based pipelines underperformed compared to incomplete data in both metrics.
(EPS)

S7 FigExtended molecular characterization of cluster-specific alterations.(A) Dot plot showing the odds ratio for recurrent cytoband deletions associated with cluster 2. Genes located within the selected biomarker cytobands (17p12, 9p21.3, 21q11.2, and 18q21.2) are labeled. The fill color of the dots indicates the $-{\text{log}}_{10}$ fasle discovery rate (FDR) value from Fisher’s exact test. (B) and (C) Scatter plots showing the relationship between CpG methylation (B-values) and normalized gene expression for *MEOX2* (B) and *TBX2* (C) across patient samples. Kendall’s correlation τ coefficients and p-values are indicated. The best-fit line and the 95% confidence bands are shown. (D) Bar plot with the proportion of affected alleles in all analyzed cytobands (top) and in the selected (17p12, 9p21.3, 21q11.2, and 18q21.2) cytobands (bottom) within each cluster. (E) Volcano plot showing the differential protein expression/phosphorylation in cluster 2 compared to cluster 1. The horizontal dashed line in B and C indicate the p-value < 0.05 threshold to assess significance. (F) Dot plot representing the top 10 KEGG pathways with highest rich factor (proportion of significantly expressed proteins to the total number of proteins in the pathway) and FDR < 0.05. Differentially expressed/phosphorylated proteins belonging to each pathway are indicated, using their gene names for simplicity. The color of the text indicates the expression/phosphorylation log fold-change (FC) in cluster 2 versus cluster 1. The fill color of the dots indicate the value of the $-{\text{log}}_{10}$ FDR of the over-representation analysis.(EPS)

S8 Fig IMOC clustering yields a large unique DEG signature and the broadest oncogenic enrichment landscape compared with classic subtype contrasts. (A) Heatmap of centroid gene-expression profiles restricted to the union of DEGs identified across subtype/cluster contrasts (*n* = 11,470 genes). DEGs were defined as FDR < 0.05 and |logFC| < 0.25. Centroids represent mean normalized expression within each subtype/cluster; values were z-scored per gene across centroids for visualization. Hierarchical clustering of centroids was performed using UPGMA agglomeration method. (B) UpSet plot summarizing overlap among DEG sets derived from IMOC (cluster 2 vs cluster 1) and classic subtype contrasts (one-vs-rest within each taxonomy). The top 15 DEG intersections are shown; bars indicate intersection sizes and total set sizes. The IMOC cluster contrast included 707 DEGs unique to cluster 2 vs cluster 1 that were not shared with any classic subtype-derived DEG set. (C) Pairwise Spearman correlations between centroid expression profiles computed in DEG-union space; dot size/color indicate Spearman correlation. Only significant correlations after correction (FDR < 0.05) have a dot shown. The numbers inside the cells indicate the value of the Spearman correlations. (D) MDS of centroid expression profiles in DEG-union space (*n* = 11,470 genes). (E) Heatmap of centroid GSVA profiles restricted to oncogenic gene sets significantly enriched by GSEA (FDR < 0.25; *n* = 27). Scores were z-scored per gene set across centroids for visualization. Hierarchical clustering of centroids was performed using UPGMA agglomeration method. (F) Number of significantly enriched oncogenic gene sets (FDR < 0.25) per contrast, highlighting the comparatively broader enrichment observed for the IMOC cluster contrast. (G) Pairwise Spearman correlations between centroid GSVA profiles in the enrichment-restricted gene-set space. Only significant correlations after correction (FDR < 0.05) have a dot shown. The numbers inside the cells indicate the value of the Spearman correlations. (H) MDS of centroid GSVA profiles computed on the enrichment-restricted oncogenic gene-set space (*n* = 27).
(EPS)

S9 Fig Cluster-associated oncogenic pathway activity persists after adjustment for classic PDAC subtype labels. For each oncogenic gene set (MSigDB C6), we fitted limma moderated linear models to test the association between IMOC cluster membership and pathway activity while adjusting for classic PDAC subtype labels. Three separate models were run, conditioning on Bailey, Collisson, or Moffitt taxonomy (facets). Points show the limma-estimated adjusted difference in GSVA score (cluster 2 - cluster 1); horizontal bars denote 95% confidence intervals. The vertical dashed line indicates no difference (0). Gene sets are ranked by log FC within each adjusted model; the plotted set corresponds to the top 10 significant (FDR < 0.05) pathways per taxonomy (union shown). Dot color encodes the Benjamini-Hochberg FDR (darker red indicates lower FDR).
(EPS)

S10 Fig Survival and disease-free progression curves stratified by classification system. Kaplan-Meier curves for (A) overall survival and (B) disease-free progression in the cohort with all four classifications available (n = 133 for overall survival, n = 100 for disease-free progression). Each panel shows the survival curves for IMOC clustering (top left), Bailey subtypes (top right), Collisson subtypes (bottom left), and Moffitt subtypes (bottom right). Shaded regions represent 95% confidence intervals. At-risk tables below each panel show the number of patients at risk and cumulative events at 6-month intervals. Survival curves intersect across follow-up in all four classifications, indicating violation of the proportional hazards assumption.
(EPS)

S11 FigRMST extreme-subtype contrasts and signal-to-noise comparison across classifications.(A) Restricted mean survival time differences ((ΔRMST) between best- and worst-prognosis subgroups within each classification, at landmark times τ∈6,12,24,36 months, which were the time points with data available for all the comparisons. For binary classifications (IMOC, Moffitt), the contrast is between the two clusters/subtypes. For multi-level classifications (Bailey, Collisson), each non-extreme subtype is contrasted against the worst-prognosis reference at τ=12 (Squamous for Bailey, QM for Collisson). Points indicate the RMST difference estimate; error bars represent 95% confidence intervals. Labels above points show the Benjamini-Hochberg (BH)-adjusted p-value within each classification. Top: overall survival; bottom: disease-free progression. (B) Z-statistics (ΔRMST / SE(ΔRMST) for each contrast in panel A, plotted across τ. The z-statistic decomposes prognostic value into effect size relative to estimation uncertainty. Dotted horizontal lines at z=±1.96 mark the threshold for nominal significance (p ≈ 0.05, unadjusted). IMOC achieves the highest signal-to-noise ratio for overall survival at τ = 6 and τ = 12, exceeding all other classifications and extreme-subtype contrasts at these landmarks. Top: overall survival; bottom: disease-free progression. BH adjusted p-values < 0.05 are indicated in bold.(EPS)

S12 FigRMST stratified analysis: IMOC prognostic separation within Moffitt subtypes.Restricted mean survival time differences (ΔRMST) between IMOC Cluster 1 and Cluster 2 patients, computed separately within each Moffitt subtype (Basal-like, squares; Classical, diamonds), at landmark times τ∈6,12,18,24,36,60 months and at maximum follow-up per stratum. Points indicate the RMST difference (in months) estimate; error bars represent 95% confidence intervals. Point fill color indicates the Benjamini-Hochberg (BH)-adjusted p-value within each Moffitt subtype across values. Labels above points show the BH-adjusted p-value at each landmark. The Moffitt classification was selected for stratified analysis because it is the only established taxonomy in which IMOC clusters are distributed across all subtypes. For overall survival, IMOC produced significant separation within both Moffitt subtypes at distinct time horizons: Basal-like at early landmarks (τ=6, BH p-value = 0.005; τ=12, BH p-value = 0.033), and Classical across most landmarks from τ=12 onward, including ΔRMST=3.9 months at τ=24 (BH p-value = 0.01). For disease-free progression, IMOC produced significant separation only within Moffitt-Classical patients (ΔRMST=4.8 months at τ=24 (BH p-value = 0.02). Within Moffitt-basal-like, no significant separation was observed for disease-free progression at any τ. Top: overall survival; bottom: disease-free progression. BH p-values < 0.05 are indicated in bold.(EPS)

S13 Fig Upset plot for entire patient cohort used in this study. (EPS)
