# Supplementary material for: Biomarker discovery and patient stratification in pancreatic cancer using incomplete multi-omics data
Source: PLoS Comput Biol. 2026 Sep 10;22(9):e1014735. doi: 10.1371/journal.pcbi.1014735 (PMC13588524; doi:10.1371/journal.pcbi.1014735)
Supplement: Supplementary file 1 [file pcbi.1014735.s001.pdf]

Biomarker discovery and patient stratification in pancreatic cancer using incomplete multi-omics data: supporting information

S1 Data preprocessing

An independent preprocessing pipeline was built for each omic modality to reduce the dimensionality of the data:

- The Reverse-Phase Protein Array (RPPA) dataset had 195 features (proteins) measured in 108 patients. Preprocessing consisted of removing features with missing information in more than 20% of the patients, and imputation of missing values using the k-nearest neighbours algorithm from the scikit-learn library (1). The final number of features was 192.
- The micro-RNA (miRNA) dataset originally had 1046 features (miRNA) measured in 147 patients. Preprocessing consisted of removing features with zero values in more than 20% of patients, and $\log_2(x+1)$ transformation (2). The final number of features was 385.
- The methylation dataset had 485577 features (CpG sites) measured in 153 patients. Preprocessing consisted of removing features with missing data in more than 20% of the patients, keeping 1% of features with the highest median absolute deviation (MAD), and removing features with a Pearson correlation higher than 0.85 (3; 4). The final number of features was 2185.
- The gene expression (RNAseq) dataset had 20501 features (genes) measured in 147 patients. Preprocessing consisted of removing features with zero values in over 20% of the patients, keeping 10% of features with the highest MAD, removing features with a Pearson correlation higher than 0.85, and $\log 2(x+1)$ transformation (4). The final number of features was 1419.
- The somatic mutations dataset had 30357 features (genes) measured in 133 patients. Preprocessing consisted of creating a binary matrix to indicate if a patient had at least one mutation in a particular gene (1) or not (0), and removing features with zero values in over 95% of the patients (5). The final number of features was 71.
- The somatic copy number alterations dataset contained 52 features (chromosomal arm segments) measured in 153 patients. All the features were used in the clustering process.

S2 Algorithm descriptions and preprocessing

We evaluated nine clustering algorithms, selected based on the availability of open-source implementations, ease of use, computational efficiency, reproducibility, and previously reported performance. In addition, we aimed to include a diverse set of approaches, including factorization-based, kernel-based, graph-based, and subspace methods. All algorithms are available in the iMML Python package (`https://github.com/ocbe-uio/imml`).

- NEighbourhood-based Multi-Omics clustering (NEMO) creates a similarity matrix between patients for each modality. The matrices for each modality are integrated into one relative similarity matrix, which is then averaged and used for clustering (6).
- Multi-Omics Factor Analysis (MOFA) representes data from several matrices into a lower dimensional space which captures major sources of variation across modalities (3).

- Data Fusion by Matrix Factorisation (DFMF) integrates all available data, enabling low-dimensional representations of certain modalities to borrow information from related objects in other modalities and revealing hidden associations (7).
- Projective Incomplete Multi-View Clustering (PIMVC) projects all modalities into a common dimension, and applies a graph regularisation term to study the structure of the data and improve clustering performance (8).
- Efficient and Effective Incomplete Multi-View Clustering (EEIMVC) imputes incomplete base matrices generated by incomplete views with a learned consensus matrix, alternating between these two steps until both the base clustering and consensus matrices converge to optimal values (9).
- Late Fusion Incomplete Multi-View Clustering (LFIMVC) integrates incomplete clustering matrices generated by incomplete views by simultaneously learning a consensus clustering matrix, imputing each incomplete base matrix, and optimising the corresponding permutations matrices (10).
- Scalable Incomplete Multi-view Clustering with Adaptive Data Completion (SIMCADC) decomposes the data for each modality into asymmetric graphs that represent the relationship between a subset of features and all other features. This approach enables learning from incomplete information, with the graphs being iteratively updated to improve clustering performance (11).
- Self-representation Subspace Clustering for Incomplete Multi-view Data (IMSR) utilises an adapted self-representation subspace method to perform data imputation and self-representation learning in incomplete data cyclically, and obtain a low-rank regularised consensus coefficient matrix (12).
- Multiple Kernel K-Means with Incomplete Kernels (MKKMIK) integrates imputation and clustering into a single learning procedure by performing multiple-kernel clustering in incomplete data. Incomplete kernel matrices are imputed and combined iteratively until convergence in order to optimise clustering, and these are used to guide missing data imputation (13).

Although the data has been carefully preprocessed to reduce the number of features in each modality, individual algorithms required additional preprocessing steps in order to be able to perform correctly. As a first step common to all algorithms, constant features were removed. For NEMO, MOFA, DFMF, EEIMVC, LFIMVC, and MKKMIK, features were standardised by removing the mean and dividing by the standard deviation, rescaling the data to have a mean of 0 and a standard deviation of 1. For PIMVC, SIMCADC, and IMSR, patients were normalised individually to unit norm.

No algorithm-specific hyperparameter tuning was performed. All nine clustering methods were executed using their recommended default settings, thereby ensuring a consistent and unbiased comparison across algorithms.

S3 Cluster evaluation

The use of the cluster performance metrics to measure the quality of the clustering solutions, independent of any clinical labels or expected ground truths, allowed for an unbiased quantification of the results by evaluating only the intrinsic properties of the clusters. In this way, the patient groups formed were not influenced by any external factors, such as patient survival, and completely unsupervised stratification based on the molecular data of patients was obtained.

To be able to compare directly different conditions, silhouette and AMI scores were normalized by dividing each value by the highest score obtained in the experiment. Consequently, a normalized score of 1 represents the maximum observed performance.

For the pairwise calculation of AMI, we used only the patients in common in both iterations.

We removed clustering solutions with major cluster imbalance, where at least one of the clusters contained less than 10% of the patients. This is because clustering solutions with extremely small clusters are not meaningful for comparative analysis, and extrapolation of results to broader patient groups would be challenging.

For incomplete scenarios, four modality-wise missingness mechanisms were applied, where each mechanism illustrates a clinically-relevant case of incomplete data:

- Missing completely at random (MCAR): modalities are randomly missing.
- Missing not at random (MNAR): some samples have modalities with missing data due to external factors that influence how the data missingness presents itself. This depicts cases such as multi-center studies, where different subgroups of data are recorded depending on the center.
- Mutually exclusive missingness (MEM): viewed as an extreme extension on MNAR, only one modality is available for incomplete samples. All modalities have the same contribution to the dataset. This mechanism effectively assesses performance in scenarios with very limited data.
- Partial missing (PM): some modalities are present for all samples, whilst others are randomly missing. This could represent scenarios where some modalities are easy to obtain due to their widespread use and cost-effective acquisition, whilst others require specialized methods and machinery to collect the data.

S4 Consensus clustering

To minimise potential technical bias from applying an algorithm only once, consensus clustering was used to create the final clusters. First, the best performing algorithm (NEMO) was executed on 1024 resampling iterations to create the consensus matrix, where for each iteration a random subset of 80% of patients ($n = 123$) was selected.

The consensus clustering works by computing an $N \times N$ consensus matrix that indicates the proportion of times two patients are grouped together:

$$C(i, j) = \frac{\sum M(i, j)}{\sum I(i, j)}, \tag{S1}$$

where M is the connectivity matrix such that its (i, j)-th entry is equal to 1 if both patients i and j belong to the same cluster, and 0 otherwise; and I is the indicator matrix such that its (i, j)-th entry is equal to 1 if both items i and j are used in that iteration, and 0 otherwise.

Item consensus $m_i(k)$ was used to measure the average consensus between a patient e_i and all the other patients in cluster k, and can be defined as

$$m_i(k) = \frac{1}{N_k - 1\{e_i \in I_k\}} \sum_{\substack{j \in I_k \\ j \neq i}} C(i, j), \tag{S2}$$

where N_k is the number of patients in cluster k; $1\{\texttt{cond}\}$ is the indicator function equal to 1 when `cond` is true, and 0 otherwise; I_k is the set of patients belonging to cluster k; and C is the consensus matrix (14).

Then, k-means clustering was applied to the consensus matrix to obtain the final clusters. The stability was quantified using the pairwise AMI score, calculated cumulatively at checkpoints corresponding to 2^r resampling iterations, where $r = 1, 2, 3, ..., 10$.

S5 Multi-modal feature selection

Multi-modal feature selection using an intermediate fusion framework was used, with Adaptative BEst Subset Selection (ABESS) as the selection algorithm. To do so, feature selection using ABESS was performed individually in the copy number and methylation datasets in order to select a subset of predictors from both modalities. Both feature subsets were then concatenated and a final feature selection step using ABESS was performed to obtain the final subset of features. To compute the performance of these features at predicting cluster assignments, the MCC score was recorded across all features (both methylation and copy number), and after obtaining the final subset.

As an additional analysis, a unimodal feature selection using ABESS was performed individually in the copy number and methylation datasets for comparison.

Survival and disease-free statistical analyses were also performed by applying Cox's proportional hazards model to the features obtained, and recording the hazard ratio (HR) and p-value at specific time points. The results are shown in S3 Data.

S6 Omics analysis

For gene expression (RNA-seq), raw read counts were analysed using the edgeR (15) and limma (16) packages. Differential gene expression analyses were performed to derive gene sets associated with IMOC clusters (cluster 2 vs cluster 1) and with each classic subtype defined in Bailey/Collisson/Moffitt taxonomies using a one-vs-rest strategy within each taxonomy. Genes with a limma FDR < 0.05 and $|\log \text{fold-change (FC)}| > 0.25$ were considered differentially expressed.

For DNA methylation, β values were transformed into M-values ($M = log_2(\frac{B}{1-B})$) to improve statistical properties for differential analysis. CpGs with a limma FDR $<$ 1e-05 and $|\log \text{FC}| > 2$ were considered differentially methylated. Limma was employed for modelling, with contrasts defined between clusters. For RPPA data, normalized peptide or protein abundance data were similarly modelled using limma, with annotation matching performed to identify gene targets. Proteins with expression/phosphorylation with limma p-value < 0.05 were considered significant.

For methylomics data, Gene Set Enrichment Analysis (GSEA) were conducted using the missMethyl (17) R package. Significantly differentially methylated CpG sites (DCpGs) were filtered on the basis of log fold-change ($|\log FC| > 2$) and adjusted false discovery rate (FDR$< 1e{-}5$). CpG identifiers were annotated to genes using the IlluminaHumanMethylation450kanno.ilmn12.hg19 (18) package.

Gene expression normalized counts were used for GSEA using GSEA software (v4.4.0) (19; 20) and Molecular Signatures Database (MSigDB v2025.1) (21). The null distributions for GSEA were generated with 1000 permutations of phenotype samples. Gene sets with a FDR < 0.25 were considered enriched. For both, methylation and gene expression data, the gene set enrichment analysis was performed against the MSigDB oncogenic signature gene set (MSigDB C6).

GSVA (22) scores were computed for oncogenic gene sets (MSigDB C6) using normalized RNA-seq expression profiles. To evaluate whether IMOC-defined clusters captured pathway activity differences independent of established PDAC subtype classifications, we performed subtype-adjusted analyses using limma moderated linear models. For each gene set, we fitted the model:

$$\text{GSVA score} \sim \text{IMOC cluster} + \text{classic subtype label}$$

where IMOC cluster was encoded as a two-level factor (cluster 1 as reference) and the classic subtype label corresponded to Bailey, Collisson, or Moffitt taxonomy.

Because these taxonomies provide correlated but non-equivalent labeling of PDAC biology, models were fitted separately for each taxonomy rather than jointly.

For RPPA data, significant peptide targets were filtered at a cut-off of $p < 0.05$. Over-representation analysis of KEGG pathways was performed using clusterProfiler (23) package.

For the evaluation of the association between CNA and the cluster groups, contingency tables were constructed summarising the number of patients with altered and normal states within the

clusters. Odds ratios were calculated from 2x2 contingency tables of altered versus normal states across clusters, applying a 0.5 continuity correction to all counts. Genes located in each cytoband were retrieved using biomaRt R package (24) using the peak limit region coordinates. Fisher's exact test was used to compare proportions in each cluster.

References

1. Duan R, Gao L, Gao Y, Hu Y, Xu H, Huang M, et al. Evaluation and comparison of multi-omics data integration methods for cancer subtyping. PLoS Computational Biology. 2021 8;17. doi:10.1371/journal.pcbi.1009224.

2. Ju J, Wismans LV, Mustafa DAM, Reinders MJT, van Eijck CHJ, Stubbs AP, et al. Robust deep learning model for prognostic stratification of pancreatic ductal adenocarcinoma patients. iScience. 2021 12;24. doi:10.1016/j.isci.2021.103415.

3. Argelaguet R, Velten B, Arnol D, Dietrich S, Zenz T, Marioni JC, et al. Multi-Omics Factor Analysis—a framework for unsupervised integration of multi-omics data sets. Molecular Systems Biology. 2018 6;14. doi:10.15252/msb.20178124.

4. López A, Zobolas J, Lingjærde OC, Nebdal D, Fleischer T, Aittokallio T. Explainable multi-omics deep clustering model reveals an important role of DNA methylation in pancreatic ductal adenocarcinoma. Nordic Machine Intelligence. 2024 8;4:26-42. doi:10.5617/nmi.10584.

5. Crawford J, Christensen BC, Chikina M, Greene CS. Widespread redundancy in -omics profiles of cancer mutation states. Genome Biology. 2022 12;23. doi:10.1186/s13059-022-02705-y.

6. Rappoport N, Shamir R. NEMO: Cancer subtyping by integration of partial multi-omic data. Bioinformatics. 2019 9;35:3348-56. doi:10.1093/bioinformatics/btz058.

7. Žitnik M, Zupan B. Data fusion by matrix factorization. IEEE Transactions on Pattern Analysis and Machine Intelligence. 2015 1;37:41-53. doi:10.1109/TPAMI.2014.2343973.

8. Deng S, Wen J, Liu C, Yan K, Xu G, Xu Y. Projective Incomplete Multi-View Clustering. IEEE Transactions on Neural Networks and Learning Systems. 2024 8;35:10539-51. doi:10.1109/TNNLS.2023.3242473.

9. Liu X, Zhu X, Li M, Tang C, Zhu E, Yin J, et al. Efficient and Effective Incomplete Multi-View Clustering. Proceedings of the AAAI Conference on Artificial Intelligence. 2019 7;33:4392-9. doi:10.1609/aaai.v33i01.33014392.

10. Liu X, Zhu X, Li M, Wang L, Tang C, Yin J, et al. Late Fusion Incomplete Multi-View Clustering. IEEE Transactions on Pattern Analysis and Machine Intelligence. 2019;41:2410-23. doi:10.1109/TPAMI.2018.2879108.

11. He WJ, Zhang Z, Wei Y. Scalable incomplete multi-view clustering with adaptive data completion. Information Sciences. 2023 11;649:119562. doi:10.1016/j.ins.2023.119562.

12. Liu J, Liu X, Zhang Y, Zhang P, Tu W, Wang S, et al. Self-Representation Subspace Clustering for Incomplete Multi-view Data. MM 2021 - Proceedings of the 29th ACM International Conference on Multimedia. 2021 10:2726-34. doi:10.1145/3474085.3475379.

13. Liu X, Zhu X, Li M, Wang L, Zhu E, Liu T, et al. Multiple Kernel k-Means with Incomplete Kernels. IEEE transactions on pattern analysis and machine intelligence. 2020 5;42:1191-204. doi:10.1109/TPAMI.2019.2892416.

14. Monti S, Tamayo P, Mesirov J, Golub T, Sebastiani P, Kohane IS, et al. Consensus Clustering: A Resampling-Based Method for Class Discovery and Visualization of Gene Expression Microarray Data. Machine Learning. 2003;52:91-118. doi:https://doi.org/10.1023/A:1023949509487.

15. Chen Y, Chen L, Lun ATL, Baldoni P, Smyth GK. edgeR v4: powerful differential analysis of sequencing data with expanded functionality and improved support for small counts and larger datasets. Nucleic Acids Research. 2025;53(2):gkaf018. doi:10.1093/nar/gkaf018.

16. Ritchie ME, Phipson B, Wu D, Hu Y, Law CW, Shi W, et al. limma powers differential expression analyses for RNA-sequencing and microarray studies. Nucleic Acids Research. 2015;43(7):e47. doi:10.1093/nar/gkv007.

17. Phipson B, Maksimovic J, Oshlack A. missMethyl: an R package for analysing methylation data from Illuminas HumanMethylation450 platform. Bioinformatics. 2015:btv560.

18. Hansen KD. IlluminaHumanMethylation450kanno. ilmn12.hg19: Annotation for Illumina's 450k methylation arrays; 2021. R package version 0.6.1.

19. Mootha VK, Lindgren CM, Eriksson KF, Subramanian A, Sihag S, Lehar J, et al. PGC-1α-responsive genes involved in oxidative phosphorylation are coordinately downregulated in human diabetes. Nature Genetics. 2003 Jul;34(3):267-73. doi:10.1038/ng1180.

20. Subramanian A, Tamayo P, Mootha VK, Mukherjee S, Ebert BL, Gillette MA, et al. Gene set enrichment analysis: A knowledge-based approach for interpreting genome-wide expression profiles. Proceedings of the National Academy of Sciences. 2005;102(43):15545-50. doi:10.1073/pnas.0506580102.

21. Liberzon A, Subramanian A, Pinchback R, Thorvaldsdóttir H, Tamayo P, Mesirov JP. Molecular signatures database (MSigDB) 3.0. Bioinformatics. 2011 05;27(12):1739-40. doi:10.1093/bioinformatics/btr260.

22. Hänzelmann S, Castelo R, Guinney J. GSVA: gene set variation analysis for microarray and RNA-Seq data. BMC Bioinformatics. 2013;14:7. Available from: https://doi.org/10.1186/1471-2105-14-7. doi:10.1186/1471-2105-14-7.

23. Xu S, Hu E, Cai Y, Xie Z, Luo X, Zhan L, et al. Using clusterProfiler to characterize multiomics data. Nature Protocols. 2024 Nov;19(11):3292-320. doi:10.1038/s41596-024-01020-z.

24. Durinck S, Spellman PT, Birney E, Huber W. Mapping identifiers for the integration of genomic datasets with the R/Bioconductor package biomaRt. Nature Protocols. 2009;4:1184-91.
