# Supplementary material for: Biomarker discovery and patient stratification in pancreatic cancer using incomplete multi-omics data
Source: PLoS Comput Biol. 2026 Sep 10;22(9):e1014735. doi: 10.1371/journal.pcbi.1014735 (PMC13588524; doi:10.1371/journal.pcbi.1014735)
Supplement: Supplementary file 2 [file pcbi.1014735.s028.pdf]

Point-by-point responses

Editor's comments

The study addresses the urgent clinical need for better patient stratification in pancreatic ductal adenocarcinoma (PDAC) by applying unsupervised machine learning algorithms to incomplete multi-omics TCGA-PDAC data. Through a systematic benchmark of nine clustering methods across six omic layers, the authors identify DNA methylation and copy number alterations (CNA) as the most informative combination, derive two robust clusters (IMOC) with marked differences in survival and recurrence within the first 18–24 months, and propose a minimal methylation biomarker panel and CNA-based model for risk stratification. The subsequent integrative analysis highlighting differences in tumor mutational burden, chromosomal deletions (e.g., 17p12, 9p21.3), and key oncogenic pathways underscores the biological plausibility and potential clinical impact of the findings.

Multi-omics approaches and advanced computational methodologies are highly aligned with current scientific trends and the growing body of literature on integrative omics for precision oncology. The methodological framework, large-scale benchmarking effort, and emphasis on incomplete multi-omics data are very much in line with the type of research we seek to promote. These strengths were echoed by the three independent reviewers, all of whom rated the manuscript highly for novelty, rigor, and relevance, assigning a unanimous recommendation of "major revision."

However, a key limitation noted by reviewers, and central to my own assessment, is the lack of external validation, which substantially weakens the translational robustness of the proposed stratification and biomarkers. The manuscript falls squarely within my field of expertise, and I find the work both valuable and potentially impactful. To address this, I recommend inviting the authors to perform external validation (e.g., evaluating the clustering framework and biomarker performance in the publicly available CPTAC-PDA cohort, which offers reasonably comparable omics profiles to TCGA-PDAC despite its high-risk enrichment). This can provide a potential test of generalizability.

All in all, given the reviewers' constructive feedback and the study's high potential, I propose proceeding with a "major revision" decision prior to initiating the full review process, allowing the authors to strengthen their arguments.

Response. We highly appreciate the editor's very positive evaluation of our work, and the possibility to revise and improve our manuscript based on the constructive feedback of the reviewers. We have addressed each of the reviewer's comments below, particularly focusing on those regarding the external validation of our initial results. Following the editor's and the reviewers' suggestions, we have now included an external validation of the clustering framework and biomarkers in the main body of the manuscript, under new section 'Validation using the CPTAC cohort', where we demonstrate the prognosis value of our findings in an independent cohort.

Reviewer #1

Summary

The manuscript presents a benchmark of nine multi-view clustering algorithms for incomplete multi-omics data and concludes that NEMO provides the most robust performance. While this comparison is valuable within the multi-view clustering literature, the set of baseline methods considered appears incomplete and limits the strength of the conclusions.

Comments

C1. While the manuscript benchmarks nine multi-view clustering algorithms, the set of baselines is incomplete and does not fully support the claim that the proposed approach improves patient stratification. In particular:

- **C1.1**. The manuscript does not include simple single-modality clustering baselines (e.g., clustering based on methylation alone), despite the finding that methylation is the most informative modality.

  Response. We thank the reviewer for emphasizing the importance of including single-modality baseline methods to contextualize the performance of the proposed multi-modal clustering approaches. To address this point, we have conducted additional analyses using hierarchical clustering and spectral clustering, as suggested in comment below (C1.2), and which are two widely used and well-established methods that provide appropriate single-modality baselines for comparison with our multi-modal approaches. More specifically, each individual modality included in the original study (DNA methylation, RNA-seq, miRNA, RPPA, somatic mutations, and copy number alterations, CNA) was clustered independently using both of these algorithms. The experimental setup was kept identical to that used in the original benchmark.

  These additional analyses showed that single-modality clustering consistently achieved substantially lower General performance score (GPS) values than the corresponding multi-modal approaches. Considering all 63 evaluated configurations (57 multi-modal combinations and 6 individual modalities), the single-modality methods ranked at the lowest end of the performance comparison, indicating that integrating multiple omics layers provides a clear advantage in terms of cluster quality and stability (Fig 1).

  Importantly, it is also worth noting that the use of single omics force the exclusion of patients that do not have that omic data available, while our multi-omic approach allows for the inclusion of all patients, even when one of the modalities is not present.

  In our original manuscript, we indeed stated that methylation was the most informative modality. However, we have clarified in the revised version that methylation was the most informative modality when integrated with other data types, rather than when used in isolation. Biologically, DNA methylation regulates gene activity, hence it is expected to have a downstream influence on other omics

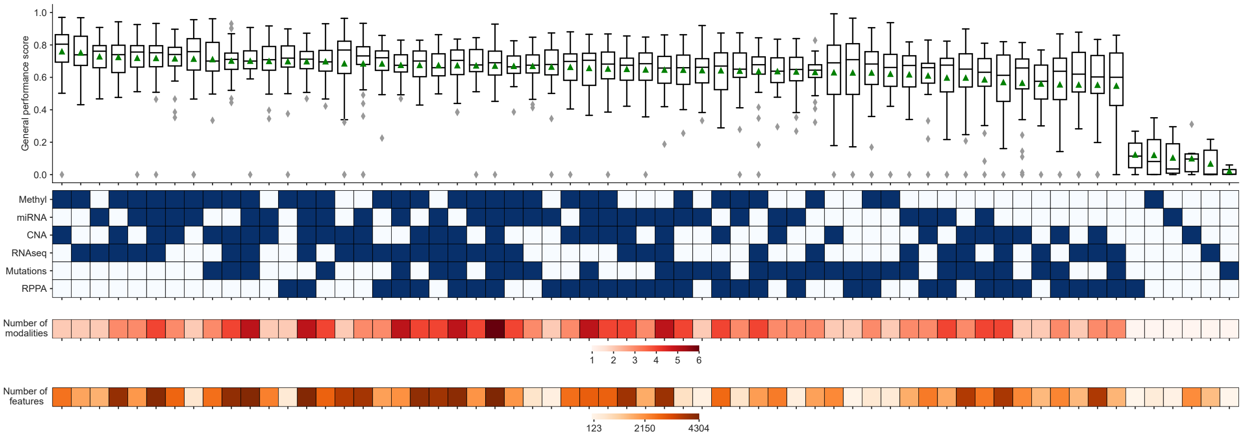

Figure 1: **Benchmarking of modality combinations.** Evaluation of the clustering performance across the different combination of omic modalities. The most informative combination was DNA methylation and copy number alterations. The general performance score was computed as the harmonic mean of silhouette and adjusted mutual information scores, reflecting cluster compactness and stability, respectively. Combinations are ordered by decreasing mean score (green triangle). First heatmap indicates modalities present in combination (blue), second heatmap indicates total number of modalities in each combination, and third heatmap shows total number of features in each combination.

layers driven by gene transcription, such as transcriptomics or proteomics. Consequently, methylation serves as a foundational layer that, when combined with downstream omics data, provides a more comprehensive and predictive model than any single modality could achieve alone.

We have updated Fig. 2A and lines 110-112 in the revised manuscript to describe theses analyses and results, and S1 Fig has been adapted to account for the single-modality baseline results.

- **C1.2**. There is no comparison with standard clustering methods such as hierarchical clustering, or spectral clustering.

  Response. We thank the reviewer for highlighting the need to compare our proposed methods against standard clustering approaches. As described above, we performed single-modality clustering using hierarchical clustering and spectral clustering, two widely used methods that serve as baseline approaches.

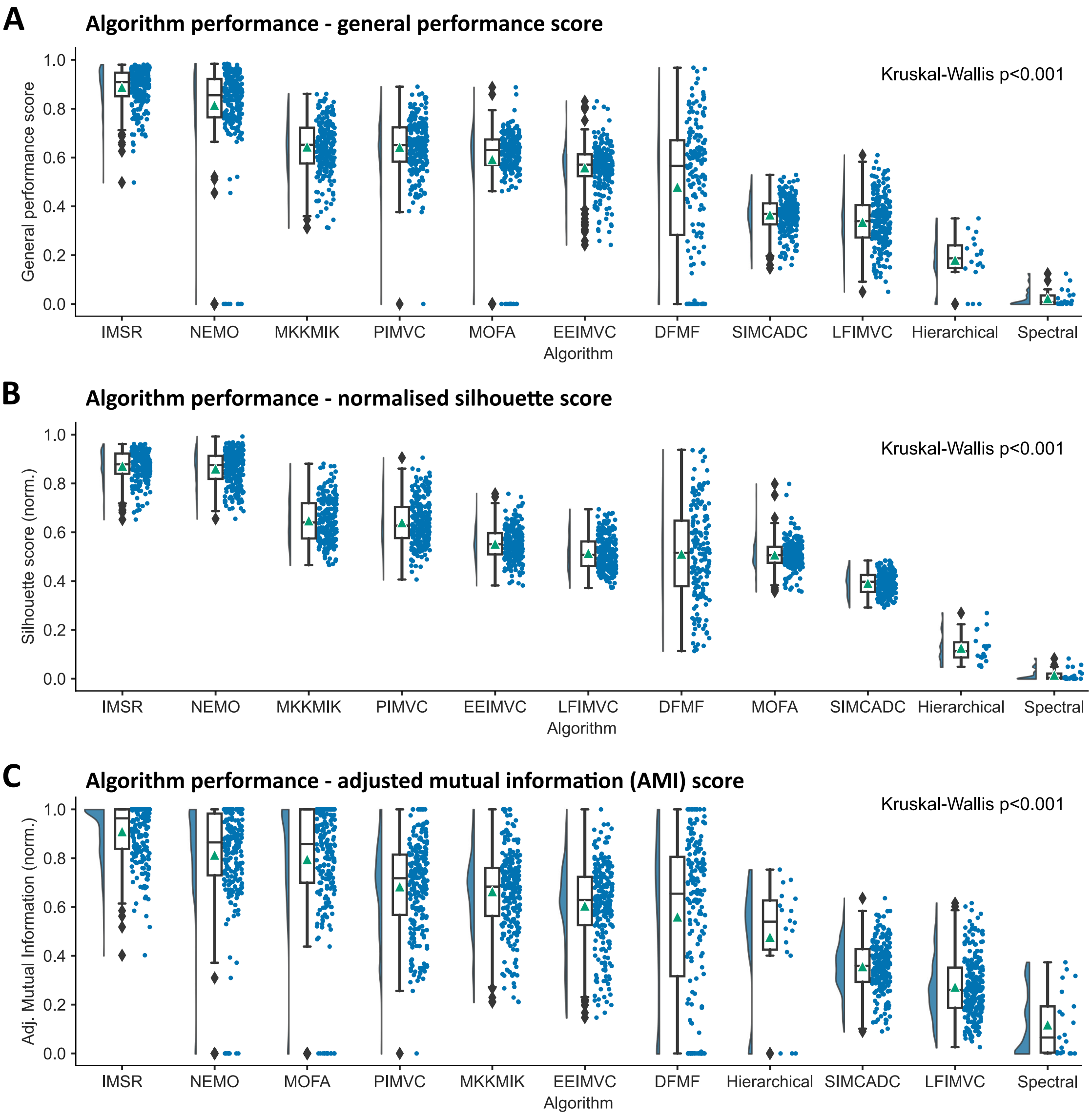

Figure 2: **Algorithm performance across combination of omic modalities.** (A) General performance score (GPS); (B) Normalized silhouette score; (C) Normalized AMI score. Hierarchical and spectral clustering of individual modalities yielded lower GPS values than the multi-modal methods in the benchmark. The algorithms are ordered by decreasing mean general performance score (green triangle).

Supplementary Figure S3 has been updated to include the results from these baseline analyses. Overall, hierarchical and spectral clustering achieved statistically decreased GPS values than the multi-modal clustering methods (Fig. 2). In particular, these single-modality approaches consistently produced the lowest silhouette scores, indicating reduced cluster compactness. Although hierarchical clustering achieved higher adjusted mutual information (AMI) values than SIMCADC and LFIMVC in some cases, its overall GPS remained lower due to its poorer silhouette performance.

Taken together, these results demonstrate that integrating complementary information across multiple omics layers generally leads to more robust and better-separated clusters than the analyses based on individual data modalities alone, further supporting the value of the proposed multi-modal framework.

- **C1.3**. The manuscript does not evaluate imputation-based pipelines, where missing modalities are first imputed and then standard integration methods are applied.

  Response. We thank the reviewer for highlighting the importance of assessing whether imputation-based approaches could provide an effective alternative for handling missing data.

  To address this comment, we compared our best-performing configuration (NEMO applied directly to incomplete methylation and CNA data with two clusters) against three standard imputation-based workflows (mean, median and K-nearest neighbors, KNN), where missing modalities were first imputed before clustering. Performance was assessed using the GPS and the robustness metric to determine whether imputation provided any advantage over NEMO's native handling of incomplete data.

  The results showed that clustering directly on incomplete data consistently outperformed the imputation-based workflows (Fig 3). In terms of robustness, all the imputation approaches remained relatively constant within the 0.6-0.85 range across all levels of missingness, whereas NEMO maintained robustness above 0.8, even when only 20% of the original data remained available (Fig 3A). Similar trends were observed for the GPS: performance of the imputation-based pipelines deteriorated rapidly as the proportion of missing data increased, while the direct incomplete-data approach exhibited a more gradual decline that closely reflected the progressive loss of information (Fig 3B). Only KNN-based imputation showed a progressive decline in the GPS, but with GPS levels below of those achieved by using NEMO directly on the data without any imputation.

  Overall, these findings indicate that, in this setting, NEMO's ability to operate directly on incomplete

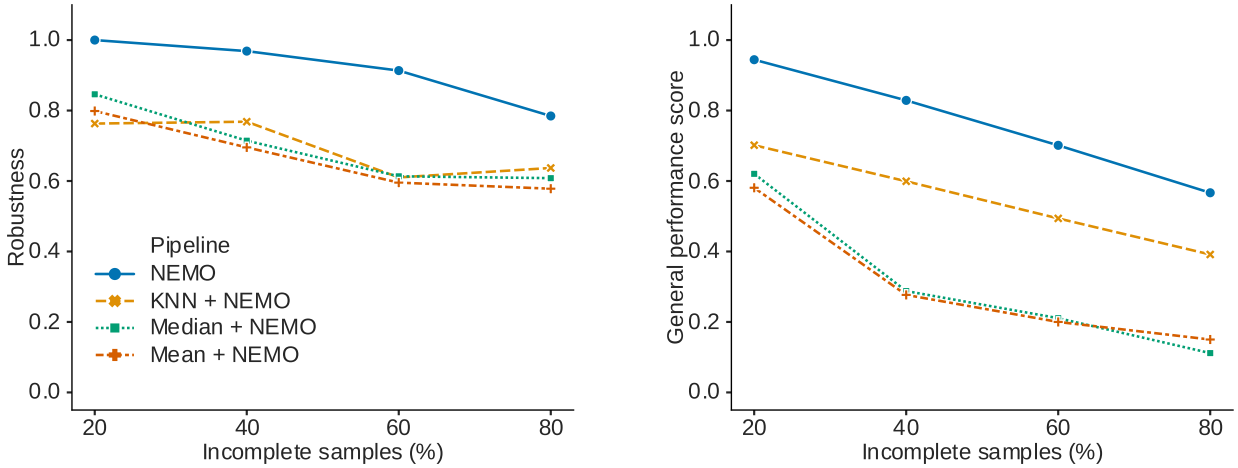

Figure 3: **Comparison of imputation-based pipelines versus incomplete data handling using the methylation and copy number (CNA) modalities.** The analysis employed the NEMO algorithm with two clusters. Comparison of (A) robustness and (B) general performance score across varying percentages of missing data. Robustness was quantified by comparing clusters obtained with missing data to those found in complete data using the adjusted mutual information score. Imputation-based pipeline underperformed compared to incomplete data handling in term of both metrics.

multi-omics data provides a clear advantage over simpler imputation strategy, yielding both greater robustness and improved clustering performance.

We have included these results in Fig. S6 and lines 130-135 of the revised manuscript.

- **C1.4**. The manuscript does not compare against clinical or survival-based models for clinical relevance. Without these comparisons, it is unclear whether the proposed framework provides a meaningful improvement over simpler or more widely used approaches.

  Response. We thank the reviewer for raising the important issue of benchmarking our approach against clinical or survival-based predictive models.

  In this context, survival outcomes were used as an external validation endpoint to assess the clinical relevance of the discovered patient clusters, rather than as a target variable during model development.

  A direct comparison of the unsupervised methods with supervised survival models presents several challenges in our dataset. First, the benchmark cohort comprises only 89 patients, meaning that splitting the data into training and testing subsets would substantially reduce the effective sample size available for model training and validation. Consequently, the resulting performance estimates would be highly variable and potentially unreliable. While resampling-based approaches such as cross-validation could be considered, the limited cohort size and high dimensionality of the multi-omics data would still make robust model evaluation challenging.

  Second, our study specifically focuses on the integration of incomplete multi-modal datasets. To the best of our knowledge, only two survival prediction frameworks have been designed to handle incomplete multi-modal data: MultiSurv [1], and the method proposed by Song *et al.* [2]. MultiSurv is a large deep learning model that was originally trained on more than 10,000 patients, and is therefore not well suited to a dataset of this size. The approach of Song *et al.* assumes that histopathology images are available for all patients, an assumption that is not satisfied in our dataset, as histopathology images were not included in the analysis and none of the available omics modalities is complete across all patients.

  For these reasons, we chose to perform unsupervised clustering using the full cohort and subsequently evaluate the resulting patient groups through survival analysis. This strategy maximizes the available statistical power, avoids introducing survival information into the clustering process, and enables an unbiased assessment of the clinical relevance of the identified subtypes.

C2. All analyses are conducted on a single dataset. As a result, the clustering solution is not validated on independent cohorts. The identified biomarkers are not tested for generalizability. The robustness of the findings to dataset-specific effects is unclear.

Response. We thank the reviewer for highlighting the importance of external validation. We fully agree that demonstrating reproducibility in an independent cohort is essential to establish the robustness and generalizability of our findings. To address this point, and as recommended by the editor, we used the CPTAC-PDA cohort for validation.

As described in the revised manuscript (lines 399-412), we developed a CNA-based predictive model capable of assigning new samples to the molecular subtypes identified originally in the TCGA-PDAC cohort. Because CNA measurements in CPTAC are provided at the gene level, whereas TCGA reports CNA at the chromosomal-region level, we harmonized the two datasets by aggregating gene-level measurements within each chromosomal region. This procedure showed a high concordance with the original data, with agreement ranging from 95% to 100% across the four prognostically relevant regions included in the model (0.95, 0.99, 1 and 1 for 21q11.2, 17p12, 18q21.2 and 9p21.3, respectively).

Application of the predictive model to the CPTAC cohort successfully reproduced the prognostic stratification results observed in TCGA. Patients assigned to the two clusters exhibited significantly different survival outcomes (ΔRMST = 6.741 months, $p = 0.047$; Fig 4A), supporting the clinical relevance of the identified patient subtypes in an independent patient population.

Importantly, key biological characteristics were also reproduced. In the TCGA cohort, the poor-prognosis subtype was characterized by elevated tumor mutational burden (TMB). Consistent with this observation, the corresponding subtype in CPTAC displayed significantly higher TMB values ($p = 0.019$) (Fig 4B). Furthermore, the chromosomal losses at 9p21.3, 17p12, 18q21.2, and 21q11.2 that contributed to subtype

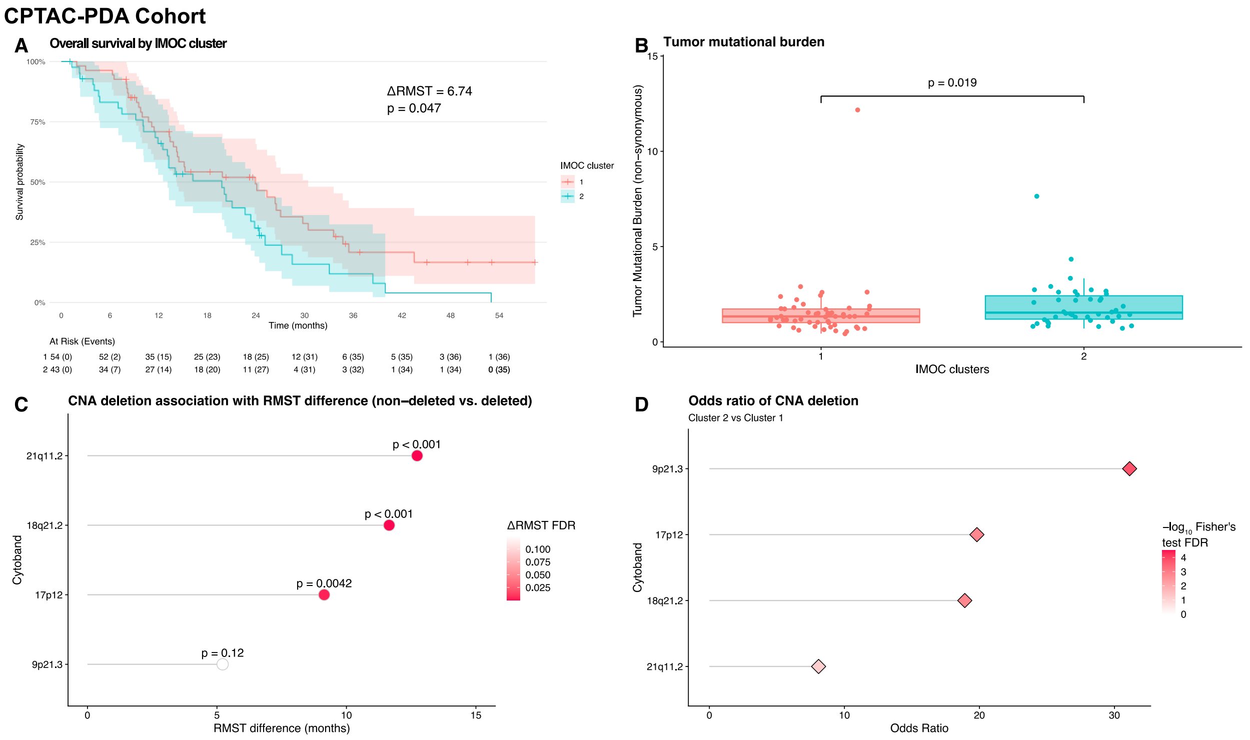

Figure 4: **Validation of IMOC clusters and biomarkers using a CNA-based stratification model in the independent CPTAC-PDA cohort.** (A) Cluster 2 (high-risk) exhibited significantly poorer survival compared to Cluster 1 (low-risk), with a RMST difference of 6.74 months ($p = 0.047$) (B) The high-risk Cluster 2 showed significantly elevated TMB compared to Cluster 1 ($p = 0.019$) (C) Association between specific cytoband deletions and RMST. Three of the four stratification features (18q21.2, 17p12, and 21q11.2) demonstrated significant associations with reduced survival (RMST difference ranging from 5 to 13 months; $p < 0.05$), while 9p21.3 was non-significant ($p = 0.1245$) (D) Odds ratio for the four copy number features used for the stratification. The high-risk phenotype is strongly associated with the presence of these chromosomal losses

definition and prognosis in TCGA were likewise associated with survival in CPTAC, with three of the four regions reaching statistical significance (Fig 4C, D). As in the discovery TCGA cohort, these alterations occurred more frequently in the poor-prognosis subtype.

We note that a direct validation of the methylation-based findings was not feasible because methylation data were at different levels of resolution in the two cohorts: gene-level in CPTAC and CpG-level in TCGA. Consequently, corresponding methylation measurements could not be extracted for external validation. Nevertheless, this limitation further highlights the important advantage of the CNA-based classifier. As we discussed in the manuscript (lines 571-574), CNA measurements are generally more robust across platforms and therefore more readily transferable to independent cohorts and potential clinical applications.

Overall, the external validation analyses demonstrate that both the prognostic value of the identified subtypes and their underlying biological characteristics are reproducible in an independent cohort. To reflect these additions, we have incorporated a new Results section entitled “Validation using the CPTAC cohort” (lines 399-412), expanded the Discussion (lines 629-641) and Methods (lines 670-677) accordingly, and added a new figure (Fig. 8 in the revised manuscript) presenting the validation results.

C3. The biomarker discovery process is performed after clustering and uses cluster labels as the target variable. I.e., features are selected to best reproduce the clustering solution. Classification performance is evaluated using these same cluster labels. This does not demonstrate that the biomarkers are predictive of clinically meaningful outcomes.

Response. We thank the reviewer for this important comment regarding the biomarker discovery strategy. We agree that selecting features based on their ability to reproduce an unsupervised clustering solution and subsequently evaluating those same features against the same clusters does not constitute an independent assessment of clinically meaningful biomarkers. Our objective, however, was not to develop a supervised predictive model, but rather to identify a minimal set of biomarkers capable of recapitulating the molecular subtypes discovered through the multi-omics integration process, enabling assignment of future samples to these subtypes.

In this context, the goal was to determine which molecular features best represent the discovered subtypes, thereby facilitating biological interpretation and the development of simplified subtype-classification models. We agree that this analysis should therefore be interpreted as a form of structural validation, rather than as evidence of clinical prediction. Nevertheless, the clinical relevance of the identified biomarkers was shown in the independent CPTAC validation cohort, as described above, where the CNA-based classifier successfully recovered prognostically distinct patient groups (Fig 9). In addition, cytoband deletions were significantly associated with reduced survival, further supporting the association between the selected biomarkers and clinically relevant outcomes.

To avoid potential misunderstanding, we have revised the manuscript to clearly distinguish between the biomarker selection for subtype representation and the evaluation of clinical relevance through external validation (lines 575-584).

Importantly, the identified biomarkers by our multi-modal feature selection have well-established connections to PDAC and cancer biology more broadly, several of which were already highlighted in the Discussion (lines 527–570), where we report supporting evidence from both animal models and pancreatic cancer patient cohorts. Briefly: The three methylation biomarkers (cg00839579, cg06785999, cg07095230) are associated with the genes *MEOX2*, *SIX6*, and *TBX2*, respectively, all of which have documented roles in cancer biology. *MEOX2* is regulated by promoter methylation and has been identified as a key gene in a gemcitabine-resistant side population in PDAC and as part of an angiogenic gene signature in pancreatic cancer (lines 541-545 of the manuscript). *SIX6* hypermethylation has been proposed as a Universal Cancer Only Marker for early cancer screening and metastasis tracking, and is associated with radioresistance in pancreatic cancer cell lines (lines 538-541 of the manuscript). *TBX2* has been linked to metastasis and poor survival in lung cancer, and was recently identified as the molecular target of piroctone olamine in PDAC patient-derived organoids — highlighting its potential as a repurposing therapeutic target in *TBX2*-dependent PDAC (lines 530-535 of the manuscript).

Among the four copy number biomarkers (9p21.3, 17p12, 18q21.2, 21q11.2), 9p21.3 and 17p12 showed the strongest association with cluster 2 patients. The 9p21.3 deletion encompasses *CDKN2A*, a central tumor suppressor in the p53 pathway whose loss is a recurrent driver across cancers [3]. Although the overall expression of CDKN2A was not significantly altered in the gene expression data (Fig. 6C in the manuscript), we found a significant enrichment in genes affected by p53 mutation and genes defining the KRAS dependency signature in cluster 2 (Fig. 6E in the manuscript). This is consistent with reports that 9p21.3 deletions co-occur with KRAS activating mutations in PDAC [4], and suggests that cluster 2 patients may represent a high-risk group with concurrent dysregulation of p53 and KRAS pathways. The molecular characterization of the IMOC clusters opens specific avenues for differential therapeutic strategies. Cluster 2 patients show enrichment of the KRAS dependency signature and reduced mTOR pathway activation, suggesting that emerging KRAS-targeted therapies may be particularly relevant for this group while mTOR inhibition, which retains therapeutic relevance for subsets of PDAC patients [5], may be more appropriate for cluster 1, where mTOR signaling remains intact. Similarly, the identification of *TBX2* as a methylation biomarker in cluster 2 highlights piroctone olamine as a candidate repurposing strategy, given its recently demonstrated efficacy in PDAC organoid models [6]. While prospective testing of these therapeutic hypotheses lies beyond the scope of the present work, the IMOC stratification provides a molecularly grounded framework within which such hypotheses can be tested in future studies.

We have modified the Discussion in the manuscript to include these aspects (lines 620-638).

C4. The manuscript reports differences in survival and disease-free progression between clusters, particularly within the first 18 months to 2 years. However, many p-values are borderline and not consistently significant across timepoints. The survival curves appear to intersect, violating proportional hazards assumptions.

Response. We thank the reviewer for highlighting the issue of intersecting survival curves and the associated violation of the proportional hazards (PH) assumption. We agree that when survival curves cross, the assumption of a constant hazard ratio over time is unlikely to hold, limiting the interpretability of standard Cox-model estimates and global log-rank tests. To account for this, we further used the restricted mean survival time (RMST) to measure differences between the survival and disease-free curves, as this test is recommended when the proportional hazards assumption is not completely met [7].

In addition, we examined RMST differences at several clinically relevant time horizons (6 months, 1 year, 18 months, 2 years, 3 years, and 5 years). Particular attention was given to the first two years after diagnosis, as this period encompasses the majority of recurrence and mortality events in PDAC and is therefore of greatest clinical relevance for patient stratification [8]. Our RMST analyses confirmed that while the hazard ratios fluctuate over time, as expected, the cumulative survival difference between clusters remains statistically significant within the early post-diagnosis period (Fig. 4 in the manuscript).

We acknowledge that the relatively small cohort size may limit statistical power, particularly at later follow-up times where fewer patients remain at risk. For this reason, we interpret the survival analyses primarily through the RMST framework, which is less sensitive to violations of the PH assumption and provides a clinically interpretable measure of cumulative survival benefit.

For completeness, we also note that non-proportional hazards were observed not only in our proposed classification but also in several previously published and widely-used PDAC subtype frameworks (Moffitt, Bailey, and Collisson). As detailed in our response to Comment 1 from Reviewer #3, we performed an in-depth survival analysis of these three existing frameworks and found that their survival curves also intersect, evidencing a similar violation of the proportional hazards assumption, suggesting that time-varying risk patterns may be a common feature of PDAC patient stratification.

By acknowledging this limitation and utilizing RMST alongside time-stratified log-rank tests, we ensure that our reported survival differences are robust and biologically meaningful, even in the absence of a constant hazard ratio.

C5. For the benchmarking study, how hyperparameters selected for each algorithm? Were all methods tuned comparably or run with default settings?

Response. We thank the reviewer for this important question regarding hyperparameter selection.

No algorithm-specific hyperparameter tuning was performed. While we acknowledge that dedicated hyperparameter optimization may further improve the performance of individual methods, we adopted a more conservative evaluation strategy given the limited sample size and the associated risk of overfitting that would arise from splitting the data into separate training, validation, and test sets. All nine clustering methods were therefore executed using their recommended default settings, thereby ensuring a consistent and unbiased comparison across algorithms. This evaluation protocol is commonly adopted in unsupervised clustering studies [9].

The only parameter varied throughout the benchmark was the number of clusters, which was evaluated uniformly across all methods for values ranging from 2 to 5. Importantly, this was not treated as an algorithm-specific tuning procedure. Rather, the number of clusters was considered a common experimental variable applied equally to all methods in order to identify the clustering resolution that best captured the underlying structure of the cohort while remaining consistent with previously reported PDAC subtype frameworks.

We have clarified this rationale in the revised manuscript (lines 73-75 in the Appendix). We have now expanded the Methods section and supplementary material (Table S1) to provide a detailed description of the parameters and settings used for all compared methods. Specifically, we have added a table summarizing the software implementation, version, key algorithm parameters, and settings used for each of the nine clustering methods evaluated in this study. We have also updated our repository with the parameters used at each stage in our experiments for transparency and to ensure that anyone can reproduce our results.

C6. The manuscript focuses on missing modalities but does not address the mechanism of the missingness.

Response. We thank the reviewer for highlighting the importance of the missingness mechanism when evaluating methods designed for incomplete multi-omics data.

Given the large number of heterogeneous modality combinations in our analysis, we particularly chose clustering algorithms that do not require any particular missingness distribution.

Furthermore, to make sure our benchmarking was robust, we evaluated the clustering methods with

increasing fractions of missing data under four distinct modality-level missingness mechanisms, designed to represent a range of plausible scenarios encountered in multi-omics studies (described in Section S3 of the S1 Appendix):

- *Missing completely at random (MCAR): modalities are randomly missing.*
- *Missing not at random (MNAR): some samples have modalities with missing data due to external factors that influence how the data missingness presents itself. This depicts cases such as multi-center studies, where different subgroups of data are recorded depending on the center.*
- *Mutually exclusive missingness (MEM): viewed as an extreme extension on MNAR, only one modality is available for incomplete samples. All modalities have the same contribution to the dataset. This mechanism effectively assesses performance in scenarios with very limited data.*
- *Partial missing (PM): some modalities are present for all samples, whilst others are randomly missing. This could represent scenarios where some modalities are easy to obtain due to their widespread use and cost-effective acquisition, whilst others require specialized methods and machinery to collect the data.*

For the primary TCGA-PAAD analysis, no artificial missingness mechanism was imposed. Instead, we analyzed the naturally occurring missing modalities present in the original dataset. As the reasons for missing data are not explicitly recorded in TCGA, and most other large-scale studies, the true missingness mechanism cannot be determined with certainty. For this reason, we considered multiple simulated missingness scenarios in our benchmarking analyses, rather than assuming any single mechanism. The final clustering results for methylation and copy number data were derived directly from the subset of patients, where these specific modalities were available, without imputation or artificial amputation.

Reviewer #2

Summary

The authors adopted the nine state-of-the-art unsupervised clustering methods capable of dealing with missing modalities were applied to incomplete multi-omics data from PDAC patients to identify clinically meaningful patient subgroups. And the identified groups are different from previous PDAC classifications, both in their patient composition and in the oncogenic gene pathway profiles exhibited. The whole structure of the paper is reasonable and logical. However, we have some modification suggestions to help the authors further improve the quality of the manuscript.

Comments

C1. The authors compared nine methods, including seven general multi-view clustering methods and two multi-omics integration methods. However, as these two integration methods were published in 2018 and 2019 respectively, the authors should consider including integration methods published in more recent years for comparison.

Response. We thank the reviewer for the opportunity to clarify the selection of algorithms included in our benchmarking.

To the best of our knowledge, there are not any recent integrative methods that can be applied straightforwardly to our setting. There are certainly newer multi-omics integration methods, but they all come with important limitations, for example: a) they cannot handle missing modalities; 2) they are designed to integrate exactly two modalities; or 3) they assume that one modality is available for all patients. Therefore, other integrative methods could not be fairly evaluated in our setting. Instead, our selection was driven by a deliberate focus on algorithmic diversity, reproducibility, and practical applicability in clinical settings.

For our evaluation, we selected methods that (i) handle bulk multi-omics data with an arbitrary number of modalities, (ii) do not require assumptions about specific missingness distributions, and (iii) provide a

ready-to-use Python implementation suitable for straightforward application by end users. Additionally, we did not include deep learning-based approaches because they generally require substantially larger training datasets than those available in our study to achieve reliable performance. Given the relatively small size of our cohort, deep learning methods are prone to overfitting and would therefore not provide a fair comparison with the selected approaches.

Although MOFA was published in 2020 [10], it remains the standard method for the integration of incomplete multi-omics data in recent studies [11, 12, 13].

Given these considerations, we prioritized methods with robust implementations and demonstrated applicability to small-to-moderate sample sizes, which aligns with the characteristics of our study cohort. The included multi-omics integration methods were therefore selected as established baselines that have been widely used in prior comparative studies and provide a meaningful reference for evaluating the proposed approaches.

We have clarified this in lines 708-718 in the revised manuscript.

C2. For a review article, the reproducibility of experimental comparisons is crucial. Therefore, the authors are advised to provide the key parameters of the compared methods in the manuscript.

Response.

We thank the reviewer for highlighting the importance of reproducibility in comparative benchmarking studies.

To address this concern, we have expanded the Methods section (line 705) and supplementary material to provide a detailed description of the parameters and settings used for all compared methods. Specifically, we have added new Table S1 that summarize the software implementation, version, key algorithm parameters, and settings used for each of the clustering methods evaluated in this study. In our analysis, all algorithms were executed using their recommended default settings.

The benchmark consisted of two stages. First, we systematically evaluated all possible combinations of the six TCGA-PAAD data modalities (RNA-seq, RPPA, miRNA, methylation, somatic mutations, and CNA), together with cluster numbers ranging from 2 to 5. This exhaustive evaluation was used to identify the modality combination and clustering resolution that yielded the highest overall performance.

Second, using the optimal modality combination (methylation and CNA) and the selected number of clusters ($k = 2$), we evaluated algorithm performance under increasing levels of missing data (20%, 40%, 60%, and 80%), generated using the missingness mechanisms described in the Appendix (Section S3).

In addition, we have updated our public repository to include the complete parameter settings and code required to reproduce all analyses. We believe these additions substantially improve the transparency and reproducibility of the study.

C3. Since each clustering algorithm corresponds to an optimal number of clusters, the authors should clearly describe how comparisons of experimental results were conducted under varying cluster numbers.

Response. We thank the reviewer for highlighting the need to clarify how clustering results were compared across different numbers of clusters.

In our benchmarking framework, each clustering method was evaluated using ($k = 2, 3, 4, 5$) clusters across all modality combinations. For every configuration, the clustering performance was quantified using the General performance score (GPS), allowing direct comparison of results obtained under different clustering resolutions.

The initial benchmark therefore consisted of an exhaustive evaluation of all algorithms, modality combinations, and cluster numbers. Based on these analyses, two clusters yielded the highest overall performance across the benchmark and were selected as the common clustering resolution for the subsequent missing-data analyses (Fig 2B in the manuscript). Additionally, when examining the optimal number of clusters for each algorithm individually, most methods selected $k=2$, with the exception of EEIMVC, which showed a slight preference for $k=3$ (Fig 5, added to the revised manuscript in Fig S4C).

To ensure a fair comparison in the second stage of the study, all algorithms were evaluated using this optimal cluster number (2 clusters). This strategy avoids reporting confounding algorithm performance, where differences arise solely from the choice of clustering resolution, and therefore facilitates a direct comparison of each method's ability to integrate incomplete multi-omics data.

We have clarified this benchmarking strategy in the revised manuscript (lines 679-689).

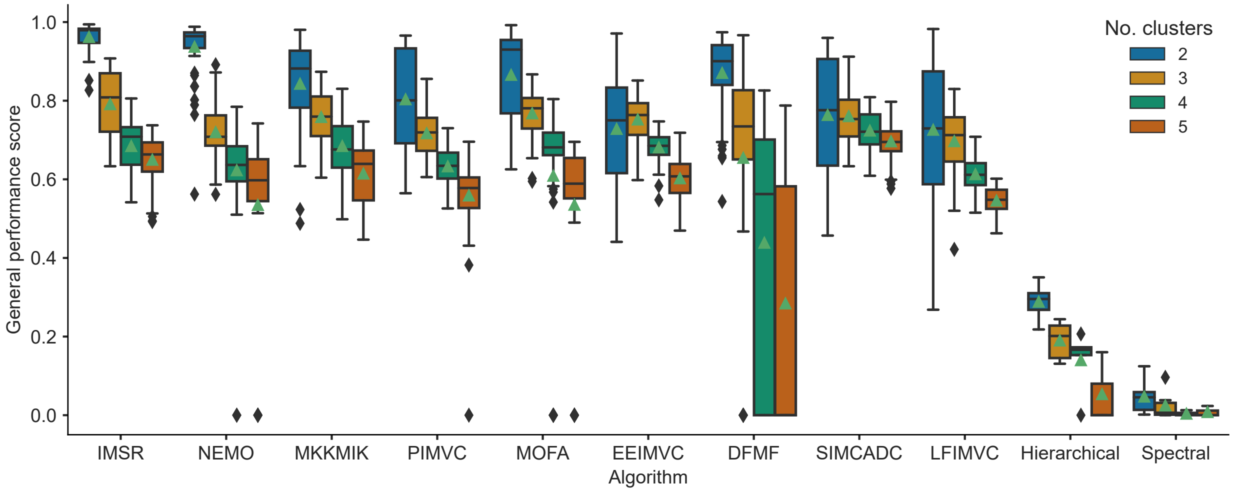

Figure 5: **Comparison of cluster performance across all algorithms.** All algorithms performed better when the number of clusters was set to 2, except EEIMVC, which demonstrated a slight better performance for three clusters.

Reviewer #3

Summary

This paper presents a machine learning method to predict patient subtypes in pancreatic cancer using publicly available multi-omics data (mRNA expression, miRNA expression, mutations, Copy Number Alterations, DNA methylation, and protein expression (RPPA, Reverse Phase Protein Array) from TCGA datasets.

Comments

C1. The results are compared with 3 previously published subtype clustering methods and distinctions are found, with improved survival and recurrence prediction. It was not clear if the support for these predictions has statistically significantly more support than the alternative 3 published subtype clusterings on the same datasets.

Response. We thank the reviewer for raising this important point. First of all, we would like to clarify that our intention was not to state a significantly improved survival and recurrence prediction of the IMOC clustering, in comparison with the three previously established PDAC subtype classifications (Bailey, Collisson, and Moffitt). Instead, our main objective was to make an unbiased clustering of the patients in TCGA-PDAC cohort using a minimal set of multi-omic biomarkers, and then investigating whether the clusters identified had differences in survival and disease recurrence. Subsequently, we demonstrated that the IMOC classification was statistically distinct from previously established subtype classifications, indicating that our clustering solution captured an orthogonal axis of PDAC heterogeneity, rather than simply relabeling of established subtypes. However, the reviewer raises an important concern, since our patient stratification could perform worse than the previously established PDAC classifications, which, while using more molecular features, could have better prognostic discrimination.

To address the reviewer's concern, we conducted a comprehensive set of comparison of overall survival and disease-free progression, restricted to patients with all four classifications (IMOC, Bailey, Collisson, Moffitt) available (n = 133 for overall survival, n = 100 for disease-free progression).

The overall survival curves and disease-free progression curves showed that all four classifications produced visually relevant separation in Kaplan-Meier curves (Fig 6A, B). Only the IMOC clusters exhibited non-intersecting overall survival trajectories. In contrast, the overall survival curves corresponding to the other classifications crossed at multiple time points, suggesting less stable patient stratification. The curve intersections evidence the violation of the proportional hazards assumption.

Because the proportional hazards assumption is violated across all the four classifications, we computed restricted mean survival time (RMST) differences between the worst- and best-prognosis subgroups within

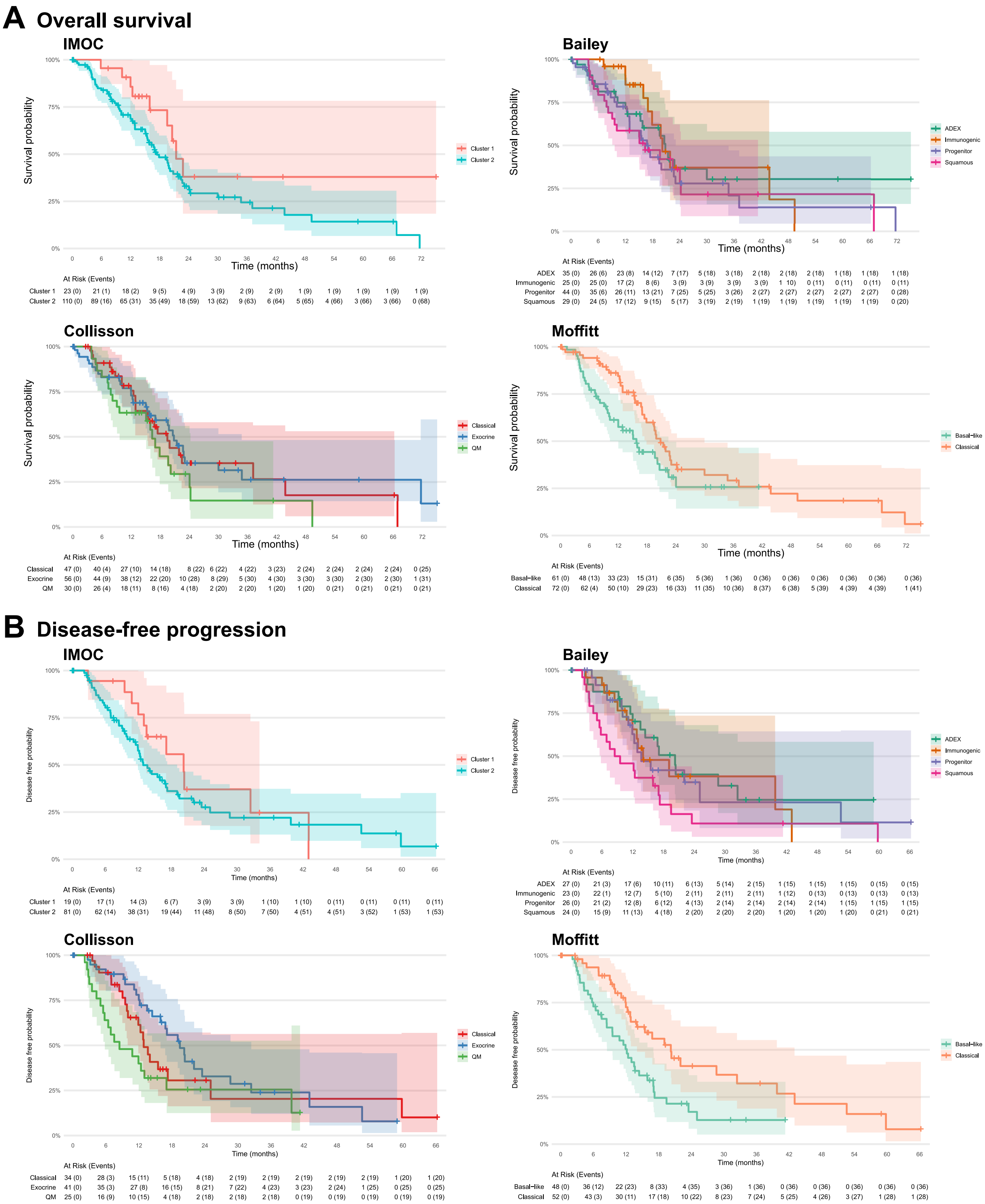

Figure 6: **Survival and disease-free progression curves stratified by PDAC classification approach.** Kaplan-Meier curves for (A) overall survival and (B) disease-free progression in the TCGA-PDAC subcohort with all four classifications available (n = 133 for overall survival, n = 100 for disease-free progression). Each panel shows the survival curves for IMOC clustering (top left), Bailey subtypes (top right), Collisson subtypes (bottom left), and Moffitt subtypes (bottom right). Shaded regions represent 95% confidence intervals. At-risk tables below each panel show the number of patients at risk and cumulative events at 6-month intervals. Survival curves intersect across follow-up in all four classifications, indicating violation of the proportional hazards assumption.

each classification, at the same landmarks. RMST is robust to proportional hazard violations and reflects the area between survival curves up to a pre-specified time point τ. For multi-level taxonomies, we also compared each non-extreme subtype to the worst-prognosis reference at τ=12 (Squamous for Bailey; QM for Collisson). The RMST extreme-subtype contrasts showed that IMOC's RMST difference is comparable in magnitude to the strongest pairwise contrasts within multi-level taxonomies (Bailey Immunogenic-vs-Squamous, Moffitt Classical-vs-Basal-like) across all time points (Fig 7A). To compare the precision-adjusted prognostic signal across classifications at each landmark, we computed the Wald z-statistic ($\Delta\widehat{RMST}/SE(\Delta\widehat{RMST})$) from RMST inference [14]. This statistic decomposes prognostic comparison into effect magnitude relative to estimation uncertainty, allowing direct comparison of binary classifiers (IMOC, Moffitt) against extreme-subtype contrasts within multi-level taxonomies (Bailey, Collisson). Importantly, the z-statistic comparison (Fig 7B) shows that IMOC achieves the highest signal-to-noise ratio for overall survival at $\tau = 6$ ($z \approx 3.6$) and $\tau = 12$ ($z \approx 3.4$), exceeding all three established taxonomies and any extreme-subtype contrast within them. This early-overall survival precision advantage reflects two structural features of IMOC: (a) its binary nature, which applies the stratification to the full cohort rather than relying on extreme-subtype contrasts within larger taxonomies, and (b) its derivation from a systematic benchmark across omics modalities, algorithms, and cluster numbers, which yielded stable cluster assignments robust to the modest event counts at early landmarks. This is clinically relevant for PDAC, since most tumor recurrences and associated mortality occur during the first 2 years post-diagnosis [8].

Finally, we performed an RMST analysis stratified by subtype to evaluate whether IMOC provides additional prognostic discrimination within existing molecular classifications. This analysis was limited to the Moffitt classification because it was the only classification in which each subtype (Classical and Basal-like) contained patients assigned to both IMOC Cluster 1 and IMOC Cluster 2 (Fig. 7A in the manuscript). In contrast, the Bailey and Collisson classifications showed little overlap between several subtypes and the IMOC clusters, precluding meaningful within-subtype survival comparisons.

The stratified RMST analysis (Fig 8) revealed distinct temporal patterns of IMOC prognostic separation within each Moffit subtype. For overall survival, IMOC clusters separated significantly within Moffitt-basal-like patients at early landmarks ($\Delta RMST = 0.5$ months at $\tau = 6$, BH adjusted p-value=0.005; $\Delta RMST = 1.9$ months at $\tau = 12$, BH adjusted p=0.033), while within Moffitt-classical patients, separation became significant from $\tau = 12$ onward and remained significant through $\tau = 36$ ($\Delta RMST = 3.9$ months at $\tau = 24$, BH adjusted p = 0.01). For disease-free progression, IMOC produced significant separation only within Moffitt-classical patients ($\Delta RMST = 4.8$ months at $\tau = 24$, BH adjusted p = 0.02), with no significant separation within Moffitt-basal-like at any τ (Fig 8). This pattern indicates that IMOC adds prognostic information beyond the binary Moffitt classification in two complementary ways: refining early overall survival stratification within Basal-like patients (where most early mortality occurs) and refining longer-term overall survival and disease-free outcomes within Classical patients.

Considered together, these analyses confirm that IMOC does not provide superior global prognostic discrimination relative to established expression-based PDAC taxonomies, but it provides complementary information in specific contexts — early overall survival stratification (concentrated within Moffitt-Basal-like patients) and refined longer-term stratification within Moffitt-Classical patients. This is consistent with IMOC's derivation from a different molecular substrate (methylation + CNA) than the expression-based comparators, which were specifically engineered for prognostic and biological discrimination. Beyond global discrimination metrics, several features distinguish the IMOC stratification: (i) the RMST contrasts between IMOC clusters are competitive in magnitude with the strongest extreme-subtype contrasts within

Bailey, Collisson, and Moffitt, while achieving the highest signal-to-noise ratio for early overall survival of any comparison tested; (ii) the IMOC clusters were derived from a systematic benchmark across six omics modalities, nine clustering algorithms, and multiple cluster numbers, with high consensus stability (Fig. 3A–C in the manuscript) — a level of methodological scrutiny not undertaken by previous classifications; (iii) IMOC's binary structure assigns every patient unambiguously to one of two clusters, in contrast to multi-level taxonomies whose strongest prognostic separations rely on extreme-subtype contrasts that exclude patients in

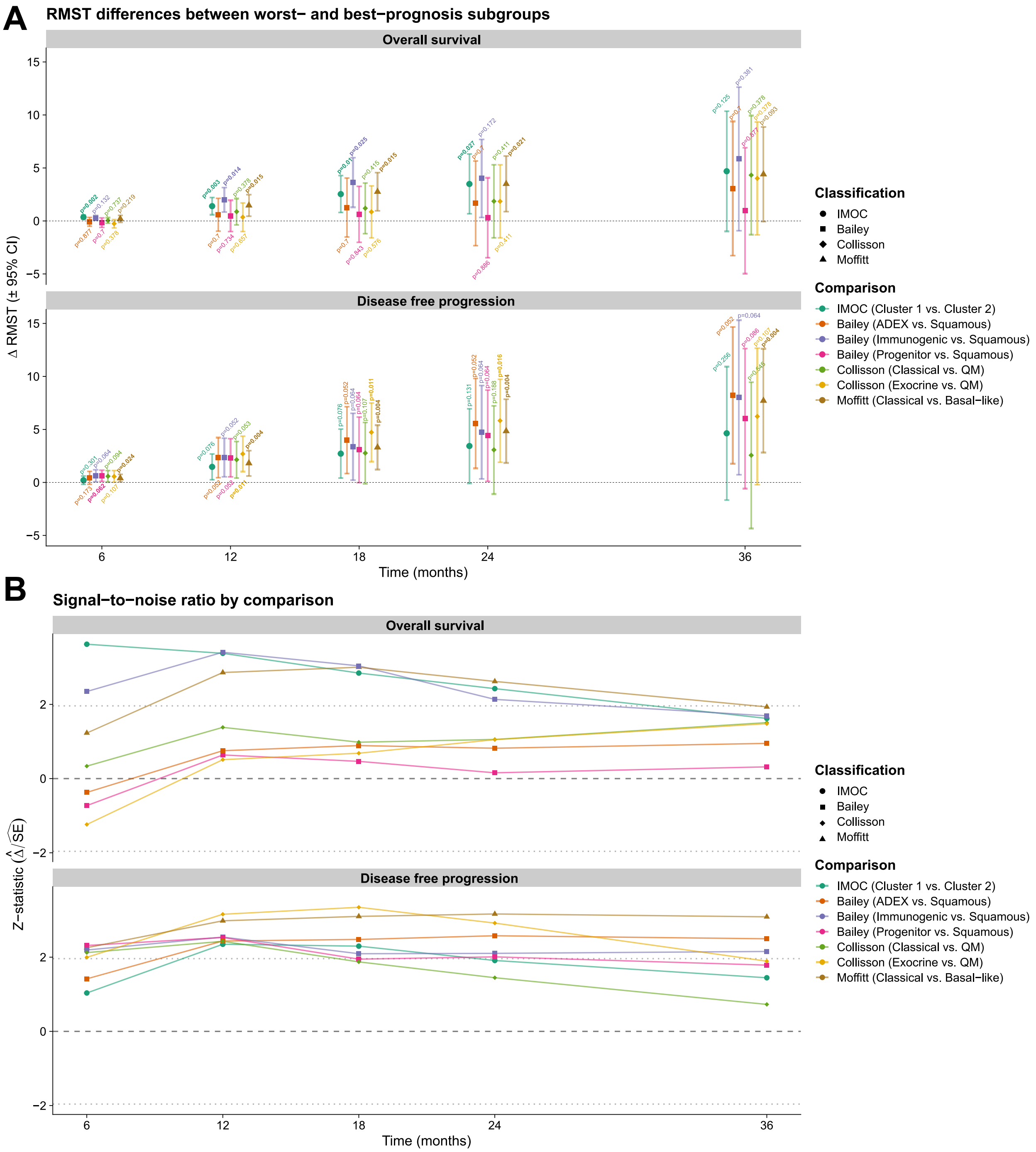

Dotted lines at z = ± 1.96 mark the threshold for nominal significance (p ≈ 0.05).

Figure 7: **RMST extreme-subtype contrasts and signal-to-noise comparison across PDAC classifications.** (A) Restricted mean survival time differences between worst- and best-prognosis subgroups within each classification, at landmark times $\tau \in 6, 12, 24, 36$ months, which were the time points with data available for all the comparisons. For binary classifications (IMOC, Moffitt), the contrast is between the two clusters/subtypes. For multi-level classifications (Bailey, Collisson), each non-extreme subtype is contrasted against the worst-prognosis reference at τ=12 (Squamous for Bailey, QM for Collisson). Points indicate the RMST difference estimate; error bars represent 95% confidence intervals. Labels above points show the Benjamini-Hochberg (BH)-adjusted p-value within each classification. Top: overall survival; bottom: disease-free progression. (B) Z-statistics (ΔRMST / SE(ΔRMST) for each contrast in panel A, plotted across τ levels. The z-statistic decomposes prognostic value into effect size relative to estimation uncertainty. Dotted horizontal lines at $z = \pm 1.96$ mark the threshold for nominal significance (p $\approx$ 0.05, unadjusted). IMOC achieves the highest signal-to-noise ratio for overall survival at $\tau = 6$ and $\tau = 12$, exceeding all other classifications and extreme-subtype contrasts at these landmarks. Top: overall survival; bottom: disease-free progression. BH adjusted p $<$ 0.05 are indicated in bold.

intermediate subtypes; and (iv) the IMOC stratification can be reduced to a four-locus CNA biomarker panel that is inherently comparable across datasets and does not require preprocessing or batch correction, enabling direct application to new samples without the gene expression profiling and normalization required by Bailey, Collisson, or Moffitt classification. Together, these features support IMOC as a methodologically robust and clinically deployable stratification that captures complementary prognostic and molecular information alongside the established PDAC taxonomies.

Additionally, the molecular orthogonality findings reported in the original submission — including the persistence of KRAS dependency pathway differences after adjustment for each established taxonomy in the limma framework, and the centroid-level separation of IMOC clusters in transcriptome and oncogenic pathway space — remain valid and unaffected by these comparisons. These analyses establish that IMOC captures a molecularly distinct axis of PDAC heterogeneity, separate from the question of whether it is the strongest single prognostic predictor.

We have included these new analyses about the overall survival and disease-free progression in the revised manuscript: Results (lines 330-397, Figs. S10-12, Supplementary Data S7, S8), and in the Discussion (lines 495-517).

C2. The authors then identify specific differentially expressed biomarker genes from within the clusters for diagnosis. It is not clear how these biomarker predictions can be further validated, so it is not clear what the disease or biological significance of the work is.

Response. We agree with the reviewer that this is an important point to address. Following the reviewer's comment (and in line with Comment 2 from Reviewer #2, and the Editor's suggestions), we used the CPTAC-PDA cohort for validation.

As described in the revised manuscript (lines 394-408), we developed a CNA-based predictive model capable of assigning new samples to the molecular subtypes identified originally in the TCGA-PDAC cohort. Because CNA measurements in CPTAC are provided at the gene level, whereas TCGA reports CNA at the chromosomal-region level, we harmonized the two datasets by aggregating gene-level measurements within each chromosomal region. This procedure showed a high concordance with the original data, with agreement ranging from 95% to 100% across the four prognostically relevant regions included in the model (0.95, 0.99, 1, 1 for 21q11.2, 17p12, 18q21.2 and 9p21.3, respectively).

Application of the predictive model to the CPTAC cohort successfully reproduced the prognostic stratification results observed in TCGA. Patients assigned to the two clusters exhibited significantly different survival outcomes (ΔRMST = 6.741 months, p = 0.047; Fig 9A), supporting the clinical relevance of the identified patient subtypes in an independent patient population.

Importantly, key biological characteristics were also reproduced. In the TCGA cohort, the poor-prognosis subtype was characterized by elevated tumor mutational burden (TMB). Consistent with this observation, the corresponding subtype in CPTAC displayed significantly higher TMB values (p = 0.019) (Fig 9B). Furthermore, the chromosomal losses at 9p21.3, 17p12, 18q21.2, and 21q11.2 that contributed to subtype

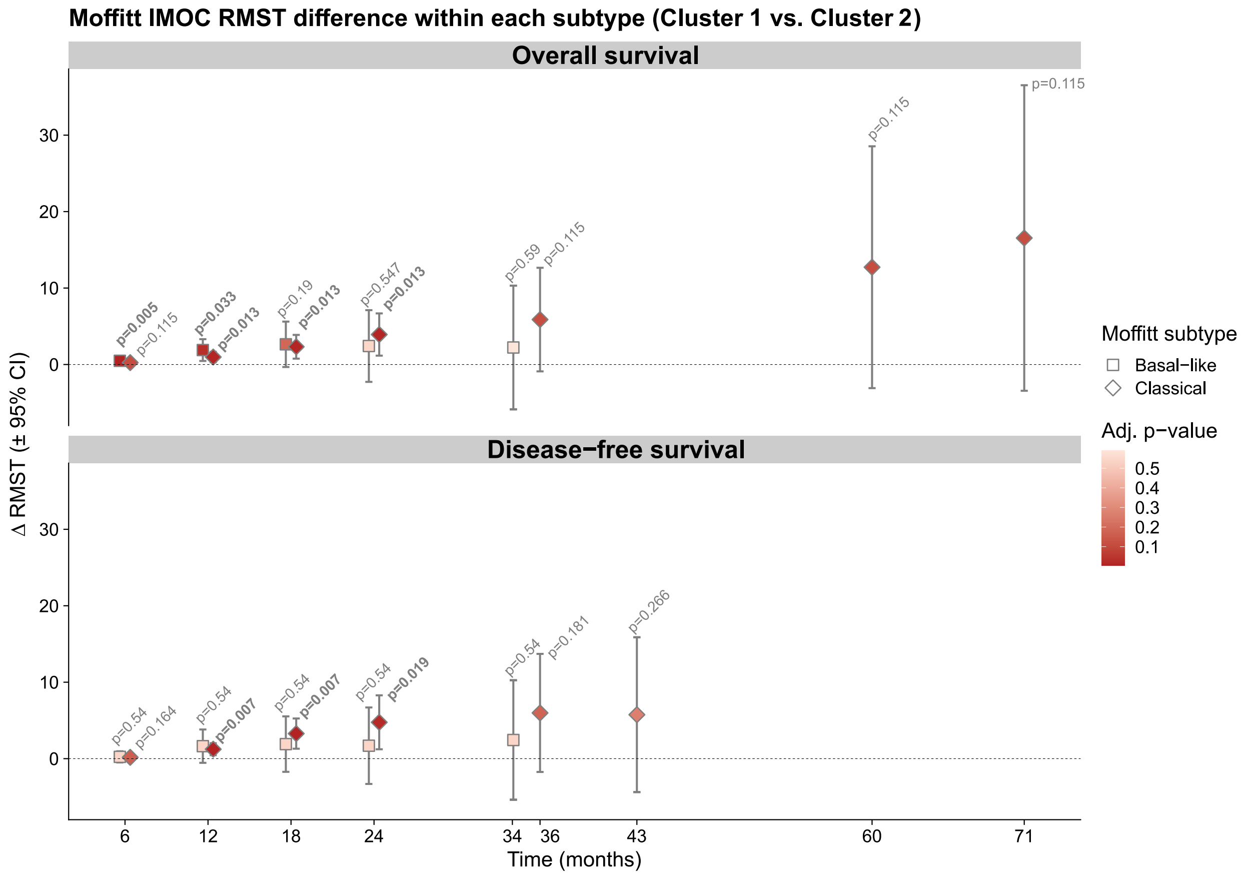

Figure 8: **RMST stratified analysis: IMOC prognostic separation within Moffitt subtypes.** Restricted mean survival time differences between IMOC Cluster 2 and Cluster 1 patients, computed separately within each Moffitt subtype (Basal-like, squares; Classical, diamonds), at landmark times $\tau \in 6, 12, 18, 24, 36, 60$ months and at maximum follow-up per stratum. Points indicate the RMST difference estimate; error bars represent 95% confidence intervals. Point fill color indicates the Benjamini-Hochberg (BH)-adjusted p-value within each Moffitt subtype across values. Labels above points show the BH-adjusted p-value at each landmark. The Moffitt classification was selected for stratified analysis because it is the only established taxonomy in which IMOC clusters are distributed across all subtypes. For overall survival, IMOC produced significant separation within both Moffitt subtypes at distinct time horizons: Basal-like at early landmarks ($\tau = 6$, BH p $= 0.005$; $\tau = 12$, BH p $= 0.033$), and Classical across most landmarks from $\tau = 12$ onward, including $\Delta RMST = 3.9$ months at $\tau = 24$ (BH p $= 0.01$). For disease-free progression, IMOC produced significant separation only within Moffitt-Classical patients ($\Delta RMST = 4.8$ months at $\tau = 24$ (BH p $= 0.02$). Within Moffitt-basal-like, no significant separation was observed for disease-free progression at any τ. Top: overall survival; bottom: disease-free progression. BH p < 0.05 are indicated in bold.

definition and prognosis in TCGA were likewise associated with survival in CPTAC, with three of the four regions reaching statistical significance (Fig 9C, D). As in the discovery TCGA cohort, these alterations occurred more frequently in the poor-prognosis subtype.

We note that a direct validation of the methylation-based findings was not feasible because methylation data were at different levels of resolution in the two cohorts: gene-level in CPTAC and CpG-level in TCGA. Consequently, corresponding methylation measurements could not be extracted for external validation. Nevertheless, this limitation further highlights the important advantage of the CNA-based classifier. As we discussed in the paper (lines 566-570), CNA measurements are generally more robust across platforms and therefore more readily transferable to independent cohorts and potential clinical applications.

Overall, the external validation analyses demonstrate that both the prognostic value of the identified sub-

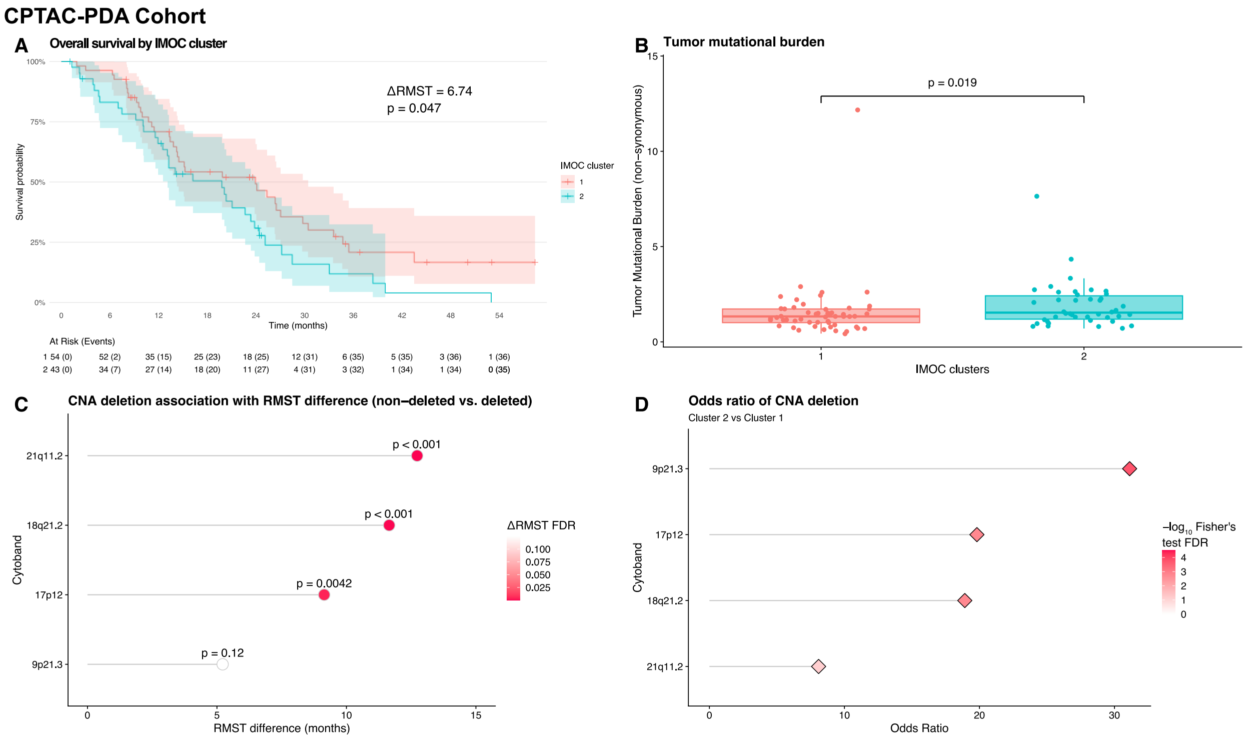

Figure 9: **Validation of IMOC clusters and biomarkers using a CNA-based stratification model in the independent CPTAC-PDA cohort.** (A) Cluster 2 (high-risk) exhibited significantly poorer survival compared to Cluster 1 (low-risk), with a RMST difference of 6.74 months ($p = 0.047$) (B) The high-risk Cluster 2 showed significantly elevated TMB compared to Cluster 1 ($p = 0.019$) (C) Association between specific cytoband deletions and RMST. Three of the four stratification features (18q21.2, 17p12, and 21q11.2) demonstrated significant associations with reduced survival (RMST difference ranging from 5 to 13 months; $p < 0.05$), while 9p21.3 was non-significant ($p = 0.1245$) (D) Odds ratio for the four copy number features used for the stratification. The high-risk phenotype is strongly associated with the presence of these chromosomal losses

types and their underlying biological characteristics are reproducible in an independent cohort. To reflect these additions, we have incorporated a new Results section entitled "Validation using the CPTAC cohort" (lines 389-405), expanded the Discussion (lines 612-624) accordingly, and added a new figure (Fig. 8 in the manuscript) presenting the validation results.

Regarding the biological significance, the biomarkers identified by our multi-modal feature selection have well-established connections to PDAC and cancer biology more broadly, several of which were already highlighted in the Discussion of the original manuscript. Briefly: The three methylation biomarkers (cg00839579, cg06785999, cg07095230) are associated with the genes *MEOX2*, *SIX6*, and *TBX2*, respectively, all of which have documented roles in cancer biology. *MEOX2* is regulated by promoter methylation and has been identified as a key gene in a gemcitabine-resistant side population in PDAC and as part of an angiogenic gene signature in pancreatic cancer (lines 541-545 of the manuscript). *SIX6* hypermethylation has been proposed as a Universal Cancer Only Marker for early cancer screening and metastasis tracking, and is associated with radioresistance in pancreatic cancer cell lines (lines 538-541 of the manuscript). *TBX2* has been linked to metastasis and poor survival in lung cancer, and was recently identified as the molecular target of piroctone olamine in PDAC patient-derived organoids — highlighting its potential as a repurposing therapeutic target in *TBX2*-dependent PDAC (lines 530-535 of the manuscript).

Among the four copy number biomarkers (9p21.3, 17p12, 18q21.2, 21q11.2), 9p21.3 and 17p12 showed the strongest association with cluster 2 patients. The 9p21.3 deletion encompasses *CDKN2A*, a central

tumor suppressor in the p53 pathway whose loss is a recurrent driver across cancers [3]. Although the overall expression of CDKN2A was not significantly altered in the gene expression data (Fig. 6C in the manuscript), we found a significant enrichment in genes affected by p53 mutation and genes defining the KRAS dependency signature in cluster 2 (Fig. 6E in the manuscript). This is consistent with reports that 9p21.3 deletions co-occur with KRAS activating mutations in PDAC [4], and suggests that cluster 2 patients may represent a high-risk group with concurrent dysregulation of p53 and KRAS pathways. The molecular characterization of the IMOC clusters opens specific avenues for differential therapeutic strategies. Cluster 2 patients show enrichment of the KRAS dependency signature and reduced mTOR pathway activation, suggesting that emerging KRAS-targeted therapies may be particularly relevant for this group while mTOR inhibition, which retains therapeutic relevance for subsets of PDAC patients [5], may be more appropriate for cluster 1, where mTOR signaling remains intact. Similarly, the identification of *TBX2* as a methylation biomarker in cluster 2 highlights piroctone olamine as a candidate repurposing strategy, given its recently demonstrated efficacy in PDAC organoid models [6]. While prospective testing of these therapeutic hypotheses lies beyond the scope of the present work, the IMOC stratification provides a molecularly grounded framework within which such hypotheses can be tested in future studies.

We have modified the Discussion in the manuscript to include these aspects (lines 629-637).

C3. An additional conceptual concern is that these datasets do not directly address the molecular mechanisms which drive the differential expression of these biomarkers. The subtype specific expression of the genes (including the nominated biomarker genes) is likely driven by variations in enhancer activity which reflect changes in underlying epigenomic cellular state or TF activity. Much recent work has been devoted to connecting changes in epigenomic state, as measured by ATAC-seq, or H3K27ac ChIP-seq, to changes in gene expression profiles, but ATAC-seq and H3K27ac is not included in the analysis of the present paper. It should be acknowledged that there is much recent promising work in this area, e.g. in gastric cancer and other cell lines (PMID: 38777607, 35817555, 34635154) and that machine learning can identify the modulated TF activity pathways from ATAC-seq (PMID: 38777607). These directly causal TFs may be better biomarkers. Recent additional work shows that modulation of enhancer activity can affect biomarker gene expression through cell state transitions (PMID: 37488417). While the current analysis probably has some modest clinical utility, the paper would have more value if placed in the context of the aforementioned work which focuses more on the direct enhancer mechanisms driving altered expression of the biomarker genes.

Response. We thank the reviewer for highlighting this important point. We fully agree that the molecular mechanisms driving the differential expression of the identified biomarkers (particularly, variations in enhancer activity, chromatin accessibility, and transcription factor activity) are not directly addressed by our current analysis. This was not the intention of the work. The TCGA dataset used in this study does not include ATAC-seq or H3K27ac ChIP-seq data for the PDAC cohort, which precludes us from evaluating these mechanistic dimensions in the present work. We acknowledge that connecting differential biomarker expression to its underlying regulatory determinants would substantially deepen the mechanistic interpretation of the IMOC stratification and that recent work, including the references kindly suggested by the reviewer, has demonstrated the value of integrating chromatin accessibility and enhancer activity data with transcriptomic profiles to identify causally modulated transcription factor pathways and enhancer-driven cell state transitions in cancer.

We have addressed this in the Discussion, explicitly acknowledging this limitation, situating our work within the broader context of enhancer-mediated regulation of cancer biomarker expression (lines 585-604).

References

[1] Luís A Vale-Silva and Karl Rohr. Long-term cancer survival prediction using multimodal deep learning. *Scientific Reports*, 11(1):13505, 2021.

[2] Yiran Song, Zaifu Zhan, Feng Xie, Nian Wang, Yifan Peng, Rui Zhang, and Mingquan Lin. A cancer-type-aware framework for robust multimodal survival prediction under missing modalities. *Briefings in Bioinformatics*, 27(2):bbag124, 2026.

[3] Francisco M Barriga, Kaloyan M Tsanov, Yu-Jui Ho, Noor Sohail, Amy Zhang, Timour Baslan, Alexandra N Wuest, Isabella Del Priore, Brigita Meškauskaitė, Geulah Livshits, et al. Machete identifies interferon-encompassing chromosome 9p21. 3 deletions as mediators of immune evasion and metastasis. *Nature Cancer*, 3(11):1367–1385, 2022.

[4] Sebastian Mueller, Thomas Engleitner, Roman Maresch, Magdalena Zukowska, Sebastian Lange, Thorsten Kaltenbacher, Björn Konukiewitz, Rupert Öllinger, Maximilian Zwiebel, Alex Strong, Hsi-Yu Yen, Ruby Banerjee, Sandra Louzada, Beiyuan Fu, Barbara Seidler, Juliana Götzfried, Kathleen Schuck, Zonera Hassan, Andreas Arbeiter, Nina Schönhuber, Sabine Klein, Christian Veltkamp, Mathias Friedrich, Lena Rad, Maxim Barenboim, Christoph Ziegenhain, Julia Hess, Oliver M. Dovey, Stefan Eser, Swati Parekh, Fernando Constantino-Casas, Jorge De La Rosa, Marta I. Sierra, Mario Fraga, Julia Mayerle, Günter Klöppel, Juan Cadiñanos, Pentao Liu, George Vassiliou, Wilko Weichert, Katja Steiger, Wolfgang Enard, Roland M. Schmid, Fengtang Yang, Kristian Unger, Günter Schneider, Ignacio Varela, Allan Bradley, Dieter Saur, and Roland Rad. Evolutionary routes and KRAS dosage define pancreatic cancer phenotypes. *Nature*, 554(7690):62–68, February 2018.

[5] Sentia Iriana, Shahzad Ahmed, Jun Gong, Alagappan Anand Annamalai, Richard Tuli, and Andrew Eugene Hendifar. Targeting mTOR in Pancreatic Ductal Adenocarcinoma. *Frontiers in Oncology*, 6, April 2016.

[6] Karabo Serala, Sanele Mdletshe, Jinming Bai, Amaal Abrahams, Odile Gayet, Loic Moubri, Nelson Dusetti, and Sharon Prince. The t-box transcription factors tbx2 and tbx3 are molecular targets of piroctone olamine in the treatment of pancreatic cancer. *Journal of Cellular and Molecular Medicine*, 29(14):e70736, 2025.

[7] Mats J. Stensrud and Miguel A. Hernán. Why Test for Proportional Hazards? *JAMA - Journal of the American Medical Association*, 323:1401–1402, 4 2020.

[8] Christopher J. Halbrook, Costas A. Lyssiotis, Marina Pasca di Magliano, and Anirban Maitra. Pancreatic cancer: Advances and challenges. *Cell*, 186:1729–1754, 4 2023.

[9] Shihao Ma, Andy G.X. Zeng, Benjamin Haibe-Kains, Anna Goldenberg, John E. Dick, and Bo Wang. Moving towards genome-wide data integration for patient stratification with Integrate Any Omics. *Nature Machine Intelligence*, 7:29–42, 1 2025.

[10] Ricard Argelaguet, Damien Arnol, Danila Bredikhin, Yonatan Deloro, Britta Velten, John C Marioni, and Oliver Stegle. Mofa+: a statistical framework for comprehensive integration of multi-modal single-cell data. *Genome biology*, 21(1):111, 2020.

[11] Qian Ye, Xiaofei Lai, Yuliang Liu, Zhengtao Zhang, Yueqiang Fu, Jie Luo, Chengjun Liu, Jun Duan, Hao Ding, Yuhan Liu, et al. Single-cell multi-omic landscape reveals anatomical-specific immune features in adult and pediatric sepsis. *Nature Immunology*, 27(1):150–165, 2026.

[12] Isabel Reinisch, Adhideb Ghosh, Falko Noé, Wenfei Sun, Hua Dong, Peter Leary, Arne Dietrich, Anne Hoffmann, Matthias Blüher, and Christian Wolfrum. Unveiling adipose populations linked to metabolic health in obesity. *Cell metabolism*, 37(3):640–655, 2025.

[13] Aanya Gupta, Koji Abe, and Holden T Maecker. Comprehensive analysis of multi-omics vaccine response data using mofa and stabl algorithms. *Frontiers in Bioinformatics*, 5:1636240, 2025.

[14] Hajime Uno, Brian Claggett, Lu Tian, Eisuke Inoue, Paul Gallo, Toshio Miyata, Deborah Schrag, Masahiro Takeuchi, Yoshiaki Uyama, Lihui Zhao, Hicham Skali, Scott Solomon, Susanna Jacobus, Michael Hughes, Milton Packer, and Lee-Jen Wei. Moving beyond the hazard ratio in quantifying the between-group difference in survival analysis. *Journal of Clinical Oncology*, 32(22):2380–2385, 2014. PMID: 24982461.
