# Supplementary material for: Biomarker discovery and patient stratification in pancreatic cancer using incomplete multi-omics data
Source: PLoS Comput Biol. 2026 Sep 10;22(9):e1014735. doi: 10.1371/journal.pcbi.1014735 (PMC13588524; doi:10.1371/journal.pcbi.1014735)
Supplement: Peer review history

# Peer review: Decision Letter 0

**DOI:** 10.1371/journal.pcbi.1014735.r001

**Authors:** Can Yang, Gian Maria Zaccaria

21 Apr 2026

Biomarker discovery and patient stratification in pancreatic cancer using incomplete multi-omics data

PLOS Computational Biology

Dear Dr. López,

Thank you for submitting your manuscript to PLOS Computational Biology. After careful consideration, we feel that it has merit but does not fully meet PLOS Computational Biology's publication criteria as it currently stands. Therefore, we invite you to submit a revised version of the manuscript that addresses the points raised during the review process.

Please submit your revised manuscript by Jun 21 2026 11:59PM. If you will need more time than this to complete your revisions, please reply to this message or contact the journal office at ploscompbiol@plos.org. When you're ready to submit your revision, log on to https://www.editorialmanager.com/pcompbiol/ and select the 'Submissions Needing Revision' folder to locate your manuscript file.

Please include the following items when submitting your revised manuscript:

* A letter that responds to each point raised by the editor and reviewer(s). You should upload this letter as a separate file labeled 'Response to Reviewers'. This file does not need to include responses to formatting updates and technical items listed in the 'Journal Requirements' section below.

* A marked-up copy of your manuscript that highlights changes made to the original version. You should upload this as a separate file labeled 'Revised Manuscript with Track Changes'.

* An unmarked version of your revised paper without tracked changes. You should upload this as a separate file labeled 'Manuscript'.

If you would like to make changes to your financial disclosure, competing interests statement, or data availability statement, please make these updates within the submission form at the time of resubmission. Guidelines for resubmitting your figure files are available below the reviewer comments at the end of this letter

We look forward to receiving your revised manuscript.

Kind regards,

Gian Maria Zaccaria

Guest Editor

PLOS Computational Biology

Can Yang

Section Editor

PLOS Computational Biology

**Additional Editor Comments:**

The study addresses the urgent clinical need for better patient stratification in pancreatic ductal adenocarcinoma (PDAC) by applying unsupervised machine learning algorithms to incomplete multi-omics TCGA-PDAC data. Through a systematic benchmark of nine clustering methods across six omic layers, the authors identify DNA methylation and copy number alterations (CNA) as the most informative combination, derive two robust clusters (IMOC) with marked differences in survival and recurrence within the first 18–24 months, and propose a minimal methylation biomarker panel and CNA-based model for risk stratification. The subsequent integrative analysis highlighting differences in tumor mutational burden, chromosomal deletions (e.g., 17p12, 9p21.3), and key oncogenic pathways underscores the biological plausibility and potential clinical impact of the findings.

Multi-omics approaches and advanced computational methodologies are highly aligned with current scientific trends and the growing body of literature on integrative omics for precision oncology. The methodological framework, large-scale benchmarking effort, and emphasis on incomplete multi-omics data are very much in line with the type of research we seek to promote. These strengths were echoed by the three independent reviewers, all of whom rated the manuscript highly for novelty, rigor, and relevance, assigning a unanimous recommendation of “major revision.”

However, a key limitation noted by reviewers, and central to my own assessment, is the lack of external validation, which substantially weakens the translational robustness of the proposed stratification and biomarkers. The manuscript falls squarely within my field of expertise, and I find the work both valuable and potentially impactful. To address this, I recommend inviting the authors to perform external validation (e.g., evaluating the clustering framework and biomarker performance in the publicly available CPTAC-PDA cohort, which offers reasonably comparable omics profiles to TCGA-PDAC despite its high-risk enrichment). This can provide a potential test of generalizability.

All in all, given the reviewers’ constructive feedback and the study’s high potential, I propose proceeding with a “major revision” decision prior to initiating the full review process, allowing the authors to strengthen their arguments.

**Journal Requirements:**

If the reviewer comments include a recommendation to cite specific previously published works, please review and evaluate these publications to determine whether they are relevant and should be cited. There is no requirement to cite these works unless the editor has indicated otherwise.

1) Some material included in your submission may be copyrighted. According to PLOS’s copyright policy, authors who use figures or other material (e.g., graphics, clipart, maps) from another author or copyright holder must demonstrate or obtain permission to publish this material under the Creative Commons Attribution 4.0 International (CC BY 4.0) License used by PLOS journals. Please closely review the details of PLOS’s copyright requirements here: PLOS Licenses and Copyright. If you need to request permissions from a copyright holder, you may use PLOS's Copyright Content Permission form.

Please respond directly to this email and provide any known details concerning your material's license terms and permissions required for reuse, even if you have not yet obtained copyright permissions or are unsure of your material's copyright compatibility. Once you have responded and addressed all other outstanding technical requirements, you may resubmit your manuscript within Editorial Manager.

Potential Copyright Issues:

i) Figure 1A: Please confirm whether you drew the images / clip-art within the figure panels by hand. If you did not draw the images, please provide (a) a link to the source of the images or icons and their license / terms of use; or (b) written permission from the copyright holder to publish the images or icons under our CC BY 4.0 license. Alternatively, you may replace the images with open source alternatives. See these open source resources you may use to replace images / clip-art:

- https://commons.wikimedia.org

- https://openclipart.org/

2)  Please ensure that the files are uploaded in the online submission form in a correct numerical order.

3) Please ensure files are unzipped prior to upload.

**Reviewers' comments:**

Reviewer's Responses to Questions

**Comments to the Authors:**

**Please note here if the review is uploaded as an attachment.**

Reviewer #1: The manuscript presents a benchmark of nine multi-view clustering algorithms for incomplete multi-omics data and concludes that NEMO provides the most robust performance. While this comparison is valuable within the multi-view clustering literature, the set of baseline methods considered appears incomplete and limits the strength of the conclusions.

1. While the manuscript benchmarks nine multi-view clustering algorithms, the set of baselines is incomplete and does not fully support the claim that the proposed approach improves patient stratification. In particular: (1) The manuscript does not include simple single-modality clustering baselines (e.g., clustering based on methylation alone), despite the finding that methylation is the most informative modality. (2) There is no comparison with standard clustering methods such as hierarchical clustering, or spectral clustering. (3) The manuscript does not evaluate imputation-based pipelines, where missing modalities are first imputed and then standard integration methods are applied. (4) The manuscript does not compare against clinical or survival-based models for clinical relevance. Without these comparisons, it is unclear whether the proposed framework provides a meaningful improvement over simpler or more widely used approaches.

2. All analyses are conducted on a single dataset. As a result, the clustering solution is not validated on independent cohorts. The identified biomarkers are not tested for generalizability. The robustness of the findings to dataset-specific effects is unclear.

3. The biomarker discovery process is performed after clustering and uses cluster labels as the target variable. I.e., features are selected to best reproduce the clustering solution. Classification performance is evaluated using these same cluster labels. This does not demonstrate that the biomarkers are predictive of clinically meaningful outcomes.

4. The manuscript reports differences in survival and disease-free progression between clusters, particularly within the first 18 months to 2 years. However, many p-values are borderline and not consistently significant across timepoints. The survival curves appear to intersect, violating proportional hazards assumptions.

5. For the benchmarking study, how hyperparameters selected for each algorithm? Were all methods tuned comparably or run with default settings?

6. The manuscript focuses on missing modalities but does not address the mechanism of the missingness

Reviewer #2: The authors adopted the nine state-of-the-art unsupervised clustering methods capable of dealing with missing modalities were applied to incomplete multi-omics data from PDAC patients to identify clinically meaningful patient subgroups. And the identified groups are different from previous PDAC classifications, both in their patient composition and in the oncogenic gene pathway profiles exhibited. The whole structure of the paper is reasonable and logical. However, we have some modification suggestions to help the authors further improve the quality of the manuscript:

1. The authors compared nine methods, including seven general multi-view clustering methods and two multi-omics integration methods. However, as these two integration methods were published in 2018 and 2019 respectively, the authors should consider including integration methods published in more recent years for comparison.

2. For a review article, the reproducibility of experimental comparisons is crucial. Therefore, the authors are advised to provide the key parameters of the compared methods in the manuscript.

3. Since each clustering algorithm corresponds to an optimal number of clusters, the authors should clearly describe how comparisons of experimental results were conducted under varying cluster numbers.

Reviewer #3: Review of “Biomarker discovery and patient stratification in pancreatic cancer using incomplete multi-omics data,“ by Paja-Garcia et al.

This paper presents a machine learning method to predict patient subtypes in pancreatic cancer using publicly available multi-omics data (mRNA expression, miRNA expression, mutations, Copy Number Alterations, DNA methylation, and protein expression (RPPA, Reverse Phase Protein Array) from TCGA datasets. The results are compared with 3 previously published subtype clustering methods and distinctions are found, with improved survival and recurrence prediction. It was not clear if the support for these predictions has statistically significantly more support than the alternative 3 published subtype clusterings on the same datasets. The authors then identify specific differentially expressed biomarker genes from within the clusters for diagnosis. It is not clear how these biomarker predictions can be further validated, so it is not clear what the disease or biological significance of the work is. An additional conceptual concern is that these datasets do not directly address the molecular mechanisms which drive the differential expression of these biomarkers. The subtype specific expression of the genes (including the nominated biomarker genes) is likely driven by variations in enhancer activity which reflect changes in underlying epigenomic cellular state or TF activity. Much recent work has been devoted to connecting changes in epigenomic state, as measured by ATAC-seq, or H3K27ac ChIP-seq, to changes in gene expression profiles, but ATAC-seq and H3K27ac is not included in the analysis of the present paper. It should be acknowledged that there is much recent promising work in this area, e.g. in gastric cancer and other cell lines (PMID: 38777607, 35817555, 34635154) and that machine learning can identify the modulated TF activity pathways from ATAC-seq (PMID: 38777607). These directly causal TFs may be better biomarkers. Recent additional work shows that modulation of enhancer activity can affect biomarker gene expression through cell state transitions (PMID: 37488417). While the current analysis probably has some modest clinical utility, the paper would have more value if placed in the context of the aforementioned work which focuses more on the direct enhancer mechanisms driving altered expression of the biomarker genes.

**********

**Have the authors made all data and (if applicable) computational code underlying the findings in their manuscript fully available?**

The PLOS Data policy requires authors to make all data and code underlying the findings described in their manuscript fully available without restriction, with rare exception (please refer to the Data Availability Statement in the manuscript PDF file). The data and code should be provided as part of the manuscript or its supporting information, or deposited to a public repository. For example, in addition to summary statistics, the data points behind means, medians and variance measures should be available. If there are restrictions on publicly sharing data or code —e.g. participant privacy or use of data from a third party—those must be specified.

Reviewer #1: Yes

Reviewer #2: Yes

Reviewer #3: None

**********

PLOS authors have the option to publish the peer review history of their article (what does this mean?). If published, this will include your full peer review and any attached files.

If you choose “no”, your identity will remain anonymous but your review may still be made public.

**Do you want your identity to be public for this peer review?**  For information about this choice, including consent withdrawal, please see our Privacy Policy.

Reviewer #1: No

Reviewer #2: No

Reviewer #3: No

**Figure resubmission:**

After uploading your figures to PLOS’s NAAS tool - https://ngplosjournals.pagemajik.ai/artanalysis, NAAS will process the files provided and display the results in the "Uploaded Files" section of the page as the processing is complete. If the uploaded figures meet our requirements (or NAAS is able to fix the files to meet our requirements), the figure will be marked as "fixed" above. If NAAS is unable to fix the files, a red "failed" label will appear above. When NAAS has confirmed that the figure files meet our requirements, please download the file via the download option, and include these NAAS processed figure files when submitting your revised manuscript.

**Reproducibility:**

To enhance the reproducibility of your results, we recommend that authors of applicable studies deposit laboratory protocols in protocols.io, where a protocol can be assigned its own identifier (DOI) such that it can be cited independently in the future. Additionally, PLOS ONE offers an option to publish peer-reviewed clinical study protocols. Read more information on sharing protocols at https://plos.org/protocols?utm_medium=editorial-email&utm_source=authorletters&utm_campaign=protocols

# Author comment: Author response to Decision Letter 1

**DOI:** 10.1371/journal.pcbi.1014735.r002

18 Jul 2026

Attachment Submitted filename: response_letter.pdf

# Peer review: Decision Letter 1

**DOI:** 10.1371/journal.pcbi.1014735.r003

**Authors:** Can Yang, Gian Maria Zaccaria

17 Aug 2026

Dear Mr. López,

We are pleased to inform you that your manuscript 'Biomarker discovery and patient stratification in pancreatic cancer using incomplete multi-omics data' has been provisionally accepted for publication in PLOS Computational Biology.

Before your manuscript can be formally accepted you will need to complete some formatting changes, which you will receive in a follow up email. A member of our team will be in touch with a set of requests.

Please note that your manuscript will not be scheduled for publication until you have made the required changes, so a swift response is appreciated.

IMPORTANT: The editorial review process is now complete. PLOS will only permit corrections to spelling, formatting or significant scientific errors from this point onwards. Requests for major changes, or any which affect the scientific understanding of your work, will cause delays to the publication date of your manuscript.

Should you, your institution's press office or the journal office choose to press release your paper, you will automatically be opted out of early publication. We ask that you notify us now if you or your institution is planning to press release the article. All press must be co-ordinated with PLOS.

Thank you again for supporting Open Access publishing; we are looking forward to publishing your work in PLOS Computational Biology.

Best regards,

Gian Maria Zaccaria

Guest Editor

PLOS Computational Biology

Can Yang

Section Editor

PLOS Computational Biology

***********************************************************

The Authors have adequately addressed all the concerns raised by the Reviewers. My final recommendation is therefore to accept the manuscript in its current form. Best regards.

Reviewer's Responses to Questions

**Comments to the Authors:**

**Please note here if the review is uploaded as an attachment.**

Reviewer #1: The revision has addressed my concerns

Reviewer #2: The author has already answered all the questions I care about.

Reviewer #3: Although the p-value for the CPTAC-PDA cohort validation is marginal, the authors have thoroughly addressed all of my concerns.

**********

**Have the authors made all data and (if applicable) computational code underlying the findings in their manuscript fully available?**

The PLOS Data policy requires authors to make all data and code underlying the findings described in their manuscript fully available without restriction, with rare exception (please refer to the Data Availability Statement in the manuscript PDF file). The data and code should be provided as part of the manuscript or its supporting information, or deposited to a public repository. For example, in addition to summary statistics, the data points behind means, medians and variance measures should be available. If there are restrictions on publicly sharing data or code —e.g. participant privacy or use of data from a third party—those must be specified.

Reviewer #1: Yes

Reviewer #2: Yes

Reviewer #3: None

**********

PLOS authors have the option to publish the peer review history of their article (what does this mean?). If published, this will include your full peer review and any attached files.

If you choose “no”, your identity will remain anonymous but your review may still be made public.

**Do you want your identity to be public for this peer review?**  For information about this choice, including consent withdrawal, please see our Privacy Policy.

Reviewer #1: No

Reviewer #2: No

Reviewer #3: No

# Editor report: Acceptance letter

**DOI:** 10.1371/journal.pcbi.1014735.r004

**Authors:** Can Yang, Gian Maria Zaccaria

PCOMPBIOL-D-26-00385R1

Biomarker discovery and patient stratification in pancreatic cancer using incomplete multi-omics data

Dear Dr López,

I am pleased to inform you that your manuscript has been formally accepted for publication in PLOS Computational Biology. Your manuscript is now with our production department and you will be notified of the publication date in due course.

The corresponding author will soon be receiving a typeset proof for review, to ensure errors have not been introduced during production. Please review the PDF proof of your manuscript carefully, as this is the last chance to correct any errors. Please note that major changes, or those which affect the scientific understanding of the work, will likely cause delays to the publication date of your manuscript.

Soon after your final files are uploaded, unless you have opted out, the early version of your manuscript will be published online. The date of the early version will be your article's publication date. The final article will be published to the same URL, and all versions of the paper will be accessible to readers.

For Research, Software, and Methods articles, you will receive an invoice from PLOS for your publication fee after your manuscript has reached the completed accept phase. If you receive an email requesting payment before acceptance or for any other service, this may be a phishing scheme. Learn how to identify phishing emails and protect your accounts at https://explore.plos.org/phishing.

Thank you again for supporting PLOS Computational Biology and open-access publishing. We are looking forward to publishing your work!

With kind regards,

Sharmila Kamatchi

PLOS Computational Biology | Carlyle House, Carlyle Road, Cambridge CB4 3DN | United Kingdom ploscompbiol@plos.org | Phone +44 (0) 1223-442824 | ploscompbiol.org | @PLOSCompBiol
